# Socio-economic and demographic disparities in ownership and use of insecticide-treated bed nets for preventing malaria among rural reproductive-aged women in northern Ghana

**DOI:** 10.1371/journal.pone.0211365

**Published:** 2019-01-29

**Authors:** Edmund Wedam Kanmiki, John Koku Awoonor-Williams, James F. Phillips, Stephen Patrick Kachur, Sabastian F. Achana, James Akazili, Ayaga A. Bawah

**Affiliations:** 1 Regional Institute for Population Studies, University of Ghana, Accra, Ghana; 2 Policy, Planning, Monitoring and Evaluation Division, Ghana Health Service, Accra, Ghana; 3 Department of Population and Family Health, Mailman School of Public Health, Columbia University, New York, United States of America; 4 Navrongo Health Research Center, Ghana Health Service, Upper East Region, Navrongo, Ghana; Universidade Nova de Lisboa Instituto de Higiene e Medicina Tropical, PORTUGAL

## Abstract

**Background:**

Malaria continues to be a leading cause of morbidity and mortality in most countries in Sub-Saharan Africa. Insecticide-treated bed nets (ITNs) is one of the cost-effective interventions for preventing malaria in endemic settings. Ghana has made tremendous efforts to ensure widespread ownership and use of ITNs. However, national coverage statistics can mask important inequities that demand targeted attention. This study assesses the disparities in ownership and utilization of ITNs among reproductive-aged women in a rural impoverished setting of Ghana.

**Methods:**

Population-based cross-sectional data of 3,993 women between the age of 15 and 49 years were collected in seven districts of the Upper East region of Ghana using a two-stage cluster sampling approach. Bivariate and multivariate regression models were used to assess the social, economic and demographic disparities in ownership and utilization of ITN and to compare utilization rates among women in households owning at least one ITN.

**Results:**

As high as 79% of respondents were found to own ITN while 62% of ITN owners used them the night preceding the survey. We identified disparities in both ownership and utilization of ITNs in wealth index, occupational status, religion, and district of residence. Respondents in the relative richest wealth quintile were 74% more likely to own ITNs compared to those in the poorest quintile (p-value< 0.001, CI = 1.29–2.34) however, they were 33% less likely to use ITNs compared to the poorest (p-value = 0.01, CI = 0.50–0.91).

**Conclusion:**

Interventions aimed at preventing and controlling malaria through the use of bed nets in rural Ghana and other similar settings should give more attention to disadvantage populations such as the poor and unemployed. Tailored massages and educational campaigns are required to ensure consistent use of treated bed nets.

## Introduction

In spite of global efforts aimed at controlling and preventing malaria, it is still the leading cause of ill health, death, poverty and low productivity in most developing countries [1, 2]. The World Health Organization (WHO) estimates that in the year 2016 alone, 216 million clinical cases of malaria were recorded while 445,000 deaths occurred globally due to malaria infection [1]. Sub-Saharan Africa alone accounted for 90% of all malaria cases and 91% of deaths due to malaria infection according to the 2017 World Malaria Report [1].

In Ghana, malaria remains highly endemic, even after considerable progress has been achieved in delivering effective prevention and treatment interventions. Malaria accounts for about 38.1% of all Out-Patient Department (OPD) cases and 50% of under-five child admissions to hospitals in Ghana. Also as high as 48.4% of all under-five deaths in Ghana are attributable to malaria infection [3]. Malaria is thus ranked among the top ten causes of morbidity and mortality in Ghana [4]. The disease affects people of all ages but children under-five years of age and pregnant women are the most vulnerable groups [5].

The use of Insecticide Treated bed-Nets (ITNs) is one of the effective strategies recommended by World Health Organization (WHO) for preventing malaria infection and its consequences during pregnancy, such as maternal anemia, stillbirths and intrauterine growth restriction in malaria-endemic settings [6, 7]. ITNs have proven to be a cost-effective method of protection against malaria. It is effective in reducing approximately 50% of malaria episodes among children under-five years of age and a 17% reduction in all-cause mortality [8, 9]. In view of this, the WHO recommends the supply of treated bed-nets free of charge or at a highly subsidized fee in malaria-endemic places using a variety of approaches including mass campaigns and routine distribution channels in order to achieve greater equity of coverage [6, 10].

To promote the use of treated bed nets in Ghana, the Ministry of Health (MoH) freely distributes nets in schools, antenatal clinics and child welfare clinics [11]. Also from 2002, the government of Ghana waived taxes on the importation of treated bed-nets in an effort to make them accessible and affordable [12]. However, despite these efforts, key targets of Ghana’s National Malaria Strategic Plan (2008–2015) on treated bed-nets were not fully achieved [13]. Recent surveys in Ghana have shown that significant proportion of households who have treated nets do not actually use them [12, 14]. The most recent malaria indicator survey reveals that just about 51% of the households have a treated bed-net for every two people in the household [14]. Also, there exist rural and urban disparities in the utilization of treated bed-nets in Ghana [5, 14]. In addition, the proportion of household population who sleep under bed-nets is found to decrease with increasing wealth [12, 14].

Thus, Ghana is yet to reach universal coverage of ITNs (defined as use by 80% or more of a population in an endemic area in order to have the optimum protection [15]). There is, therefore, the need for continuous monitoring and assessment of ownership and utilization of bed-nets, especially among critical sections of the population so as to inform policy and practice in the area of malaria prevention.

Although some studies have examined treated bed-nets ownership and use in Ghana, none has focused on a predominantly rural and deprive setting [5, 14, 16, 17]. This paper, aims to identify and highlight the social, economic and demographic disparities in ownership and utilization of insecticide-treated bed-nets among reproductive-aged women in a predominantly rural setting of Ghana.

## Materials and methods

### Study setting

**Fig 1:**
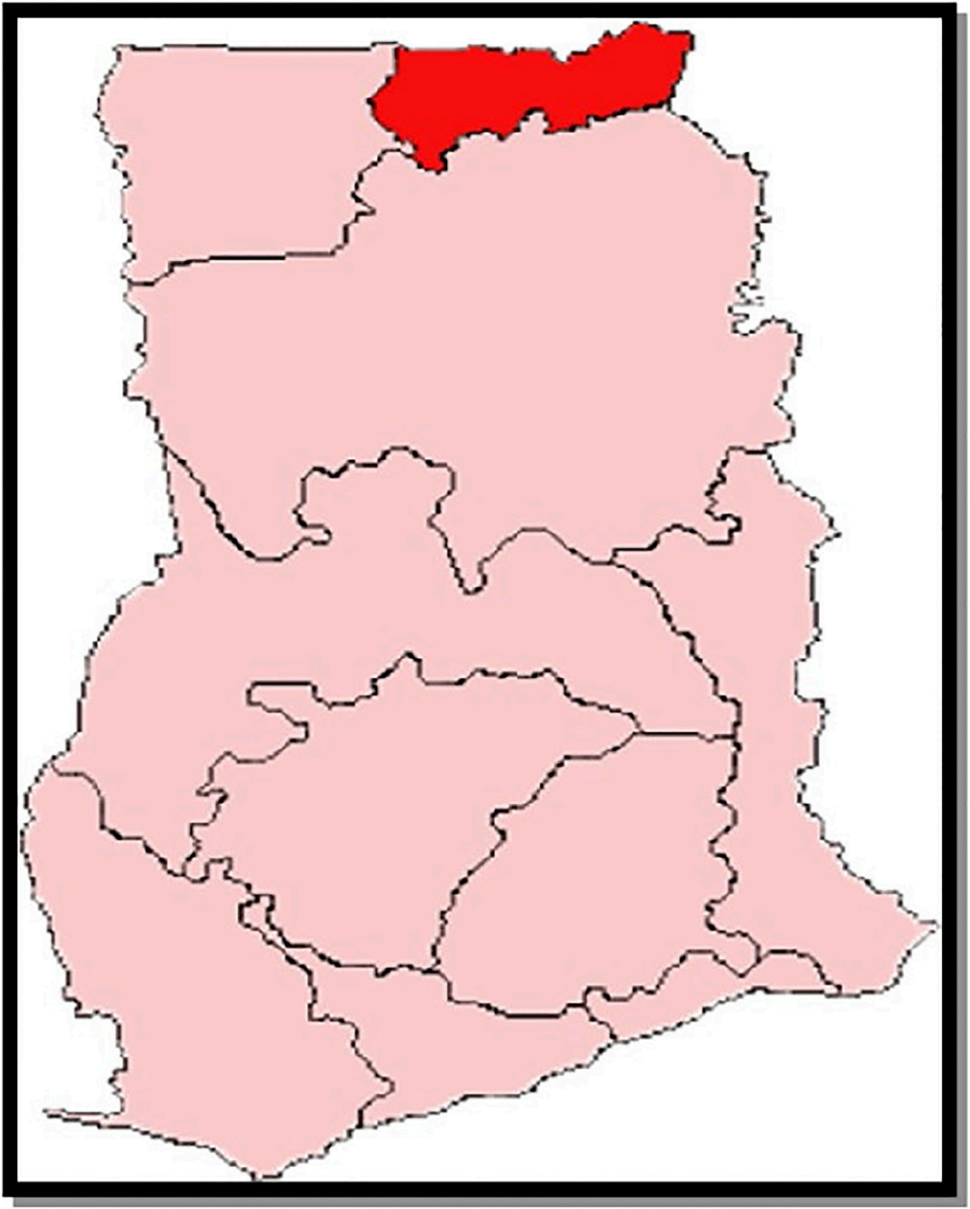
Map of Ghana showing Upper East region in red.

The data for this analysis were collected in seven districts of the Upper East region located in the extreme north-eastern part of Ghana. The Upper East region is one of the three poorest regions in the country and has a population of about 1,188,800 people [18]. It has a total land area of 8,842km with a savanna grassland vegetation [19]. It is inhabited by seven major ethnic groups and currently has 15 administrative districts. Subsistence farming is the main economic activity of people in the region [20]. Christianity, Islam and African traditional religion are the major religions of the people in the region [19]. This area is typical of most rural Sahelian African settings. Findings from this region would therefore have relevance for most rural settings in Sub-Saharan Africa. Fig 1 shows the map of Ghana indicating Upper East region.

### Source of data

Data came from an independent cross-sectional survey that collected information from women between 15 and 49 years of age in seven districts: Bolgatanga, Bongo, Builsa, Garu/Tempane, Bawku West, Talensi/Nabdam and Bawku East. The purpose was to provide end-of-project data for the evaluation of a health systems plausibility trial that was implemented by the Ghana Essential Health Intervention Project (GEHIP) to improve maternal and child survival. GEHIP was a five-year health system strengthening and research program implemented in the Upper East region from 2010 to 2015. The GEHIP approach involved strengthening the capacity of the health system around six WHO health system building blocks and improving the effectiveness of Ghana’s comprehensive Community-based Health Planning and Service (CHPS) program. Details of the GEHIP program are described elsewhere [20, 21].

### Data collection

A two-stage sampling approach was used in the data collection process. First, the Ghana Statistical Service (GSS) sampled and provided the research team a total of 66 predominantly rural Enumeration Areas (EAs) based on the 2010 Population and Housing Census. Guided by this sampling frame, physical identification of EAs was done and a household listing of all members of households in the sampled EAs was carried out. The second stage involved the sampling of households proportional to the population size of each EA. Within sampled households, all females between the ages of 15 and 49 years of age were eligible to be interviewed. The paperless “Open Data Kit” (ODK) software was used to collect the data. This technique, first developed at the University of Washington, permits instantaneous data entry, editing and correction at the time of interviews [22]. Data collection started on the 2^nd^ of October 2014 and ended on the 31^st^ of January 2015.

The survey collected data on maternal and child health indicators, fertility, family planning, universal health coverage among others. During the survey, two questions that relate to Insecticide Treated bed-net (ITNs) were; *“Does your household have an insecticide-treated bed net*?*”* and *“Did you sleep under an insecticide-treated bed-net last night”*. This analysis relies on these two questions to explore the disparities in ownership and utilization of ITNs among this cohort of the reproductive-aged women.

### Data analysis

STATA 14 software was used in analyzing the data. Basic descriptive statistics was used to describe the composition of variables while bivariate analysis was done using chi-square test of association to identify variables associated with household ownership of ITNs. Furthermore, multivariate analysis using binary logistic regression models are used to explore the disparities in ownership and utilization of ITNs. Utilisation of ITNs was examined only among respondents who reported having ITNs within their household. The variable for wealth index was generated using Principal Component Analysis (PCA) where household assets were used as a proxy for wealth. Assets used to generate wealth index were common household assets used in the study area, they include: radio, television, computer, clock, mobile phone, refrigerator, video deck, decoder, freezer, DVD/VCD, bicycle, motorcycle, motor king, animal drawn cart, car/truck, fun and decoder. Our application of PCA to generate wealth index using these household possessions as a proxy measure of wealth is consistent with previous studies conducted in this setting [19, 20, 23].

Ten independent variables were included in the analysis. Independent variables were first tested for multi co-linearity using the Variance Inflation Factor (VIF) before they were included in the logistic regression models. However, this was found not to be a problem since a mean VIF of 1.32 was found (VIF more than 20 indicates multi co-linearity). We applied sample weighting in our regressing analysis to ensure that findings are representative of the study area. In this analysis, p-values of 0.05 or below were regarded as showing significant relationship while p-values above 0.05 were regarded as not showing a significant association. Both p-values and confidence intervals have been reported in the regression models.

### Ethical considerations

The data used in this paper emanates from the Ghana Essential Health Intervention Project (GEHIP). Ethical approval was obtained from the Ethical Review Committee of the Ghana Health Service and Institutional Review Board (IRB) of the Navrongo Health Research Centre prior to the conduct of this study. Inform consent was administered to participants. Data collectors read a written informed consent form/note to participants in their preferred language and explained its content before participants who agreed to participate endorsed two copies of the form and one copy was given to the participant. This procedure was sanctioned by both ethics committees that approved of the study to be conducted. All protocols were followed to ensure confidentiality during data collection, analysis and reporting of findings.

## Results

Data from a total of 3,993 women were used in this analysis; about 79% of them reported having at least one ITN in their household. Table 1 shows the background characteristics of respondents and the results of chi-square test of association with ITN ownership within the household. Variables that were significantly associated with ownership of bed nets at bivariate level are age, level of education, functional literacy, occupation, religious affiliation, place of residence, district of residence and wealth index.

**Table 1:** Background characteristics of respondents.

Variable	Categories	Don’t have Bed Net		Have Bed Net		P-value	Total
		Freq.	%	Freq.	%		
**Age Group**	15–19	38	24.05	120	75.95	<0.001	158
	20–34	378	18.54	1661	81.46		2,039
	35–49	427	23.78	1369	76.22		1,796
**Level of Education**	None	661	22.94	2220	77.06	<0.001	2,881
	Primary/Junior High School	157	17.80	725	82.20		882
	Secondary/ Tertiary	23	10.50	196	89.50		219
	Other	2	18.18	9	81.82		11
**Functional Literacy(Ability to read and understand)**	Yes	106.00	15.77	566	84.23	<0.001	672
	No	737.00	22.19	2584	77.81		3321
**Marital Status**	Not Married yet (single)	31	24.03	98	75.97	0.396	129
	Married	743	20.85	2820	79.15		3,563
	Widowed	55	21.65	199	78.35		254
	Devoiced/Separated	14	29.79	33	70.21		47
**Marriage Type**	Polygamy	263	21.24	975	78.76	0.874	1,238
	Monogamy	474	20.62	1825	79.38		2,299
	Not specified	6	23.08	20	76.92		26
**Occupation**	Farming	395	23.54	1,283	76.46	<0.001	1,678
	Trading	156	18.01	710	81.99		866
	Artisan	99	19.04	421	80.96		520
	No occupation/housewife	161	24.54	495	75.46		656
	Civil Servant	6	9.38	58	90.63		64
	Student	10	16.13	52	83.87		62
	Other	16	10.88	131	89.12		147
**Religion**	Christianity	407	18.11	1,840	81.89	<0.001	2,247
	Traditional	112	22.18	393	77.82		505
	Islam	303	26.51	840	73.49		1,143
	No Religion	21	21.43	77	78.57		98
**Location of Residence**	Urban	74	21.76	266	78.24	0.031	340
	Semi-Urban	148	25.08	442	74.92		590
	Rural	621	20.27	2,442	79.73		3,063
**District of Residence**	Bolga M.	60	16.57	302	83.4	<0.001	362
	Bongo	76	16.03	398	84.0		474
	Builsa	94	13.39	608	86.6		702
	Garu/Tempani	265	29.94	620	70.1		885
	Bawku West	74	18.09	335	81.9		409
	Talensi/Nabdam	117	18.72	508	81.3		625
	Bawku East	149	29.27	360	70.7		509
**Wealth Index**	Quintile1 (Poorest)	232	25.89	664	74.11	<0.001	896
	Quintile2	299	22.48	1,031	77.52		1,330
	Quintile3	54	19.42	224	80.58		278
	Quintile4	155	19.23	651	80.77		806
	Quintile5 (Richest)	103	15.08	580	84.92		683

### Ownership of ITNs

Table 2 shows the multivariate analysis of ITNs ownership. Age, level of education and functional literacy were not significantly associated with ownership of ITN in the multivariate analysis.

**Table 2:** Multivariate analysis of ITNs ownership; logistic regression model.

Determinants	Odds Ratio	95% Conf. Interval		P>z
**Age group (Compared to 15–19)**				
20–34	1.46	0.97	2.21	0.070
35–49	1.08	0.70	1.66	0.731
**Level of Education (Compared with No education)**				
Primary/Junior High School	1.12	0.86	1.46	0.404
Secondary/Tertiary	1.80	1.00	3.24	0.051
Other	1.08	0.21	5.43	0.928
**Functional Literacy(Compared with Yes)**				
**No**	1.14	0.81	1.60	0.458
**Occupation (Compared with Farming)**				
Trading	1.12	0.89	1.40	0.356
Artisan	0.92	0.69	1.22	0.550
No occupation/housewife	0.71	0.55	0.90	0.005
Civil Servant	1.00	0.38	2.63	0.998
Student	0.81	0.37	1.75	0.586
Other	1.70	0.95	3.04	0.071
**Religion (Compared with Christianity)**				
Traditional religion	0.73	0.56	0.95	0.021
Islam	0.96	0.77	1.19	0.699
No religion	0.87	0.51	1.49	0.618
**Location of Residence(Compared with Urban)**				
Semi-Urban	1.39	0.96	2.00	0.078
Rural	1.87	1.35	2.60	<0.001
**District of Residence(Compared with Bolgatanga)**				
Bongo	1.56	1.05	2.33	0.028
Builsa	2.00	1.35	2.96	0.001
Garu/Tempani	0.57	0.40	0.82	0.003
Bawku West	1.11	0.74	1.66	0.613
Talensi/Nabdam	1.20	0.83	1.75	0.336
Bawku East	0.60	0.40	0.89	0.012
**Wealth Index (Compared with Quintile1;Poorest)**				
Quintile2	1.18	0.95	1.46	0.139
Quintile3	1.43	1.01	2.04	0.046
Quintile4	1.48	1.15	1.89	0.002
Quintile5 (Richest)	1.74	1.29	2.34	<0.001
Constant	1.35	0.67	2.73	0.399

On occupational status, women who were housewives or had no occupation were 29% less likely to own ITNs compared to those engaged in farming (p-value = 0.005, OR = 0. 71). For religious affiliation, respondents who were affiliated with African traditional religion were 27% less likely to own ITNs compared to respondents affiliated to Christianity (p-value = 0.021, OR = 0.73).

Place of residence was found to have a statistically significant association with ownership of ITNs. Respondents resident in rural settings were 87% more likely to own an ITN compared to residents in urban settings (p-value<0.001, OR = 1.87). District of residence was also statistically associated with ownership of ITNs; women resident in Bongo and Builsa districts were 1.56 and 2.00 times respectively more likely to have ITN compared to residents of Bolgatanga the regional capital (p-value = 0.028 and <0.001, OR = 1.56 and 2.00). However, respondents of Garu-Tempane and Bawku East districts were 43% and 40% less likely to own an ITNs compared to those of Bolgatanga (p-value = 0.003 and 0.012, OR = 0.57 and 0.60).

With regards to wealth index, it is apparent from Table 2 that the likelihood of ownership of ITN increases with higher wealth index. While those of quintile3 were 43% more likely to have ITN (p-value = 0.046, OR = 1.43), those belonging to quintiles 4 and 5 (next richest and richest categories) were 48% and 74% more likely to own ITNs compared to those in quintile1; the poorest category (p-values = 0.002 and <0.001, OR = 1.48 and 1.74 respectively).

### Utilization of ITNs

Table 3 presents multivariate analysis of utilization of ITNs the night before the survey. As shown in the table, age, level of education, functional literacy and location of residence were not significantly associated with utilization of ITN. However, occupation, religion, district of residence and wealth index were significantly associated with utilization of ITNs.

**Table 3:** Multivariate analysis of ITNs utilization; logistic regression model.

Determinants	Odds Ratio	95% Conf. Interval		P>z
**Age group (Compared to 15–19)**				
20–34	0.97	0.57	1.65	0.919
35–49	0.66	0.38	1.14	0.137
**Level of Education (Compared with No education)**				
Primary/Junior High School	0.94	0.70	1.26	0.692
Secondary/Tertiary	0.85	0.50	1.44	0.547
Other	1.12	0.22	5.75	0.890
**Functional Literacy(Compared with Yes)**				
**No**	1.05	0.74	1.50	0.772
**Occupation (Compared with Farming)**				
Trading	0.88	0.68	1.12	0.295
Artisan	1.05	0.75	1.45	0.791
No Occupation/housewife	0.90	0.67	1.21	0.480
Civil Servant	0.62	0.32	1.19	0.149
Student	0.48	0.24	0.94	0.033
Other	1.14	0.70	1.85	0.604
**Religion (Compared with Christianity)**				
Traditional Religion	1.00	0.74	1.35	0.980
Islam	1.40	1.05	1.87	0.023
No Religion	0.83	0.47	1.44	0.499
**Location of Residence(Compared with Urban)**				
Semi-Urban	1.04	0.69	1.55	0.87
Rural	0.99	0.69	1.40	0.93
**District of Residence(Compared with Bolgatanga)**				
Bongo	1.15	0.79	1.67	0.467
Builsa	1.40	0.97	2.01	0.074
Garu/Tempani	1.33	0.90	1.97	0.147
Bawku West	4.10	2.49	6.76	<0.001
Talensi/Nabdam	1.15	0.81	1.64	0.439
Bawku East	1.14	0.72	1.80	0.577
**Wealth Index (Compared with Quintile1;Poorest)**				
Quintile2	1.18	0.91	1.53	0.223
Quintile3	1.48	0.94	2.33	0.088
Quintile4	1.19	0.88	1.61	0.253
Quintile5 (Richest)	0.67	0.50	0.91	0.010
Constant	3.27	1.51	7.11	0.003

Respondents who reported that they were students were 52% less likely to used ITN compared to those engaged in farming. Also, those affiliated with Islamic religion were 40% more likely to use ITN compared with their Christian counterparts. Residents of the Bawku West district were more than four times more likely to used ITN compared to residents of Bolgatanga, the regional capital (p-value<0.001, OR = 4.10). Women in quintile 5 (richest category) were 33% less likely to use ITNs compared with those in quintile 1 (the poorest category) (p-value = 0.010, OR = 0.67).

## Discussion

Results reported in this paper show that efforts of the malaria control program in improving ownership and use of ITNs in the Upper East region is almost near the attainment of the universal coverage mark which is pegged at 80% and above. However, there are significant disparities in the ownership and use of ITNs by socio-economic and demographic factors in this rural setting. Occupation, religion, district of residence and wealth index were found to influence both ownership and utilization of ITN among reproductive-aged women in the region. Location of residence was associated with ownership of ITN but had no influence on utilization, while educational level, functional literacy and marital status was neither associated with ownership nor utilization of ITNs. Previous studies in other settings have recorded varying outcomes with regards to the association of these variables with ITN ownership. We did not find significant disparities in ownership and utilization by educational status; this is consistent with a previous study conducted in Nigeria [24]. However, some other studies found disparities in ownership and use of ITN by educational status [6, 16, 25]. According to the 2015 Ghana national demographic and health survey, 49.6% of uneducated pregnant women used ITN the night before their survey compared to 43.5% of those educated up to middle school level [5]. We are however unable to determine the statistical significance of the national survey.

Marital status was not significantly associated with ownership and use of ITNs in this analysis. A study conducted in southwestern part of Ethiopia found marital status of household head to be associated with utilization [26]. Many other studies reviewed, however, did not examine the association of marital status with ownership and utilization of ITN [16, 25, 27, 28]. This study has revealed that women without occupation were 30% less likely to own ITNs. However, it was not so when it came to utilization. Most studies reviewed did not consider the occupational status of the respondent in relation to ownership and utilization of ITN [6, 16, 25, 27]. One study from Ethiopia that examined occupation status of household head did not find any significant disparities in ITN ownership nor utilization [26].

This study has revealed that practitioners of African traditional religion were less likely to own an ITN compared to those of Christian religion. This calls for a further investigation as to why religious affiliation is associated with ownership of ITNs in the study setting. Previous studies have not examined this either. Residents of rural areas were two times more likely to possess ITN compared to residents of urban settings but there was no significant association with utilization of ITNs. Other studies have highlighted an association of rural/urban residents with ownership and use of ITNs at the household level; however, there seems to be a mix in the direction of association perhaps due to the targeting strategies employed in different settings. For instance, in a study involving two states of Nigeria, urban households were more likely to own ITN compared to their rural counterparts [29]. However, another study in the same country that assessed ownership and use among pregnant women found those who reside in urban settings to be less likely to own ITN compared to those in rural areas [24]. It however, found women living in urban areas to be almost twice more likely to use ITN compared to rural women [24]. A study in Equatorial Guinea also revealed higher utilization rates for urban dwellers than rural dwellers [25]. However, we believe that high ownership of ITN in rural northern Ghana is perhaps due to the fact that the malaria control efforts and free distribution of ITNs largely targets rural settings.

Findings of this study also reveal disparities in ITNs ownership and use among the districts in the Upper East region of northern Ghana. While two districts (Bongo and Builsa districts) were significantly more likely to ownITNs compared to Bolgatanga (the regional capital), another two (Garu-Tempane and Bawku East districts) were significantly less likely to own ITN compared to Bolgatanga. Although the reason for low ITN ownership in these two districts may not be readily known, it is on record that around 2013/2014 there was a ban on the use of motorbikes in Bawku Municipality due to an inter-tribal conflict in the area and this affected health care delivery services in Bawku Municipality. Garu-Tempane district is geographically located next to Bawku Municipal and therefore might have also suffered from accessibility challenges emanating from the Bawku conflict. The findings of this study, therefore, could be a pointer to the negative effect of conflicts on the delivery of health services.

Socio-economic status (wealth index) is a very important variable to consider when assessing the effect of health interventions or programs. It is important to know if interventions are reaching the poor as much as the relatively well off in society. In this study, we found an increase in ownership of ITN with increasing wealth. In contrast to ownership, we found that the richest category was 33% less likely to use ITN compared to the poorest category. Although only the fifth quintile was statistically significant, Our findings support those of an early study in Ghana by the Ghana Statistical Service [5]. Previous studies have also documented significant association of ITNs ownership and utilization with wealth index [6, 16, 26, 29]. However, some studies did not find any association of wealth index with ITNs ownership or use [25, 28, 30]. Among the studies that wealth index was significantly associated with ITN utilization, while most studies found high wealth index to be associated with high utilization of ITN and low wealth index to be associated with low ownership and use [6, 26, 29], one study found lower wealth index to rather be associated with high utilization [16]. This implies that based on the peculiarities of each setting and strategies used in deploying ITNs, the poor and the relatively well-off could benefit disproportionately. It should also be noted that people of high socio-economic status often have access to other methods for preventing man-vector contact and may therefore not use ITN even if they have them in their households. A study in Gabon observed that relatively wealthier people who live in houses with door and window screens often believe they are sufficiently protected from mosquito bites and therefore do not make use of ITNs even if they have them in their households [31]. This phenomenon may merit further investigation to ascertain if the use of door and window screens offers adequate protection as that of treated bed nets.

### Study limitations

As a cross-sectional quantitative study, this study is limited in understanding some of the contextual factors influencing ownership and use of ITN. Also findings of this study should be interpreted carefully to avoid over generalization. This notwithstanding, the study provides useful information to guide malaria control intervention activities in Ghana and similar settings.

## Conclusion

The high ownership and use of treated bed-nets in the Upper East Region might be due to various interventions that have been implemented in the region in recent years. In addition to UNICEF supported interventions embarked upon by the Ghana Health Service and the National Malaria Control Program, the Upper East Regional Health Administration led the implementation of intensive health systems interventions between 2010 and 2015 and it is possible these interventions contributed significantly to the results we obtained in this analysis. However, despite the relatively high percentage of ownership and use of bed nets, the study has revealed disparities by socio-economic status such as, wealth index, occupation, district of residence, location of residence and religious affiliation. It can be inferred from the review of literature that the determinants of ITNs ownership and utilization are not static. Indeed, they are context and time-specific, a system of continuous monitoring and evaluation is therefore required so that disadvantaged sections of the society can often be targeted. Interventions aimed at mitigating inequalities in distribution and ensuring consistent use of ITNs in rural Ghana and other similar settings should give more attention to disadvantage populations such as the poor and unemployed. Tailored massages and educational campaigns are required to ensure consistent use of treated bed nets.

## Supporting information

S1 File De-Identified minimal dataset. (DTA)

S1 Text Study questionnaire. (PDF)
