# Supplementary material for: Socio-economic and demographic disparities in ownership and use of insecticide-treated bed nets for preventing malaria among rural reproductive-aged women in northern Ghana
Source: PLoS One. 2019 Jan 29;14(1):e0211365. doi: 10.1371/journal.pone.0211365 (PMC6350974; doi:10.1371/journal.pone.0211365)
Supplement: S1 Text — (PDF) [file pone.0211365.s002.pdf]

UPLOAD8716276124481710292

Field	Question	Answer
Identification		
fwname *(required)*	Fieldworker, please enter your name	
geopoint	Collect the GPS coordinates of this household.<br>*GPS coordinates can only be collected when outside.*	
todaymo	This month:	1 January<br>2 February<br>3 March<br>4 April<br>5 May<br>6 June<br>7 July<br>8 August<br>9 September<br>10 October<br>11 November<br>12 December
todayyr	This year:	2013 2013<br>2014 2014<br>2015 2015
region	REGION:<br>*Response constrained to: .<3*	1 UPPER EAST<br>2 GREATER ACCRA
district *(required)*	TYPE OF LOCATION/SETTLEMENT<br>*Response constrained to: .<4*	1 Urban<br>2 Semi-urban<br>3 Rural
accessibility	LEVEL OF ACCESSBILITY<br>*Response constrained to: .<3*	1 Easy to reach<br>2 Hard to reach
community	COMMUNITY /SECTION	
ea *(required)*	EA CODE	
compound *(required)*	COMPOUND NAME	
HHnum *(required)*	HOUSEHOLD NUMBER<br>*Response constrained to: .<1000*	
peopleinHH	NUMBER OF PEOPLE IN THE HOUSEHOLD<br>*Response constrained to: .<100*	
INFORMED CONSENT		
HHconsent	"Good morning/afternoon/evening. My name is ________. I am representing the GHANA ESSENTIAL HEALTH INTERVENTION PROJECT. We are conducting a survey to assess maternal and child health and we are collecting information on households in this, and other similar communities in Ghana. Your household has been selected to participate in this survey by means of a random or chance selection process, much like taking a cup of beans from a bowl. You have been selected for interview because you your household has been selected. I would like to ask you a few questions about you and your child if I may, but you can refuse to answer any question I ask. Your participation in this survey is completely voluntary. You may terminate the interview any time. You can also refuse to participate in the study entirely. The interview will last approximately 1 hour. The information we collect from you will not be shown to anyone outside of this project. If you have any question about this study, you can contact Dr James Akazili on phone number 0244834435 or Mr. Fabian Sebastian Achana on phone number 0243213284	
HHconsented *(required)*	Do you agree to be interviewed?<br>*Response constrained to: .=1*	1 Yes<br>2 No
Household Information		
water *(required)*	What is the main source of drinking water for members of your household?	1 Piped into dwelling<br>2 Piped into yard or plot<br>3 Public tap/standpipe<br>4 Borehole<br>5 Protected well<br>6 Unprotected well<br>7 Spring<br>8 Rainwater collection<br>9 Tanker-truck<br>10 Cart with small tank/drum<br>11 River/stream

Field	Question	Answer
		12 Dam/lake/pond/canal /irrigation channel<br>13 Sachet water<br>14 Bottled water<br>other Other
water_other	Specify other.<br>*Question relevant when: selected(${water}, 'other')*	
toilet *(required)*	What kind of toilet facility do members of your household usually use?<br>*IF "FLUSH" OR "POUR FLUSH", PROBE: Where does it flush to? IF NECESSARY, ASK PERMISSION TO OBSERVE THE FACILITY.*	1 No facilities (bush/beach, etc)<br>2 Flush to piped sewer system<br>3 Flush to septic tank<br>4 Flush to pit (latrine)<br>5 Ventilated Improved Pit latrine (VIP)<br>6 Pit latrine with slab<br>7 Pit latrine without slab/open pit<br>8 Bucket<br>other Other
toilet_other	Specify other.<br>*Question relevant when: selected(${toilet}, 'other')*	
toiletshare *(required)*	Do you share this facility with other households?<br>*Question relevant when: ${toilet} >1 and ${toilet} <9*	1 Yes<br>2 No
solidwaste *(required)*	How does your household dispose refuse (solid waste)?	1 Collected<br>2 Dump into public container<br>3 compound refuse dump<br>4 Public/community dump<br>5 Dump elsewhere<br>6 Burned by household<br>7 Buried by household<br>other Other
solidwaste_other	Specify other.<br>*Question relevant when: selected(${solidwaste}, 'other')*	
liquidwaste *(required)*	How does your household dispose liquid waste?	1 Through the sewerage system<br>2 Thrown into gutter<br>3 Thrown onto compound<br>4 Thrown onto outside compound<br>5 Compound gutter<br>other Other
liquidwaste_other	Specify other.<br>*Question relevant when: selected(${liquidwaste}, 'other')*	
fuelsource *(required)*	What is the main fuel used for cooking in this household?	1 Electricity or Gas<br>2 Kerosine on stove<br>3 charcoal<br>4 firewood<br>5 millet stock<br>6 cow dung<br>7 other
fuelsource_other	Please specify<br>*Question relevant when: ${fuelsource} =7*	
Household Information > HHassets		
generated_table_list_label_37	Does your household have a:	
reserved_name_for_field_list_labels_38		1 Yes<br>2 No
HHradio *(required)*	Radio?	1 Yes<br>2 No

Field	Question	Answer
HHtv *(required)*	Television?	1 Yes<br>2 No
HHcomp *(required)*	Computer?	1 Yes<br>2 No
HHclock *(required)*	Clock?	1 Yes<br>2 No
HHmobile *(required)*	Mobile telephone?	1 Yes<br>2 No
HHfridge *(required)*	Refrigerator?	1 Yes<br>2 No
HHvideo *(required)*	Video deck?	1 Yes<br>2 No
HHfreezer *(required)*	Freezer?	1 Yes<br>2 No
HHdvd *(required)*	DVD/VCD player?	1 Yes<br>2 No
HHbike *(required)*	Bicycle?	1 Yes<br>2 No
HHmotorbike *(required)*	Motorcycle or scooter?	1 Yes<br>2 No

Field	Question	Answer
Hhmotoking *(required)*	Motorkingg?	1 Yes<br>2 No
HHcart *(required)*	Animal-drawn cart?	1 Yes<br>2 No
HHcar *(required)*	Car or truck?	1 Yes<br>2 No
Hhfan *(required)*	Fan?	1 Yes<br>2 No
Hhdecoder *(required)*	Decorder, ie MultiTV or Strong?	1 Yes<br>2 No
Household Information > Hhlivestock		
generated_table_list_label_56	How many of each of the following livestock does this household currently own?	
Hhcows *(required)*	Cattle<br>*None=0*	
Hhdogs *(required)*	Dogs<br>*None=0*	
Hhpigs *(required)*	Pigs<br>*None=0*	
Hhchickens *(required)*	fowls<br>*None=0*	

Field	Question	Answer
Hhguineafowl *(required)*	Guinea fowls<br>*None=0*	
Hhsheep *(required)*	Sheep<br>*None=0*	
Hhgoats *(required)*	Goats<br>*None=0*	
Hhdonkeys *(required)*	Donkeys<br>*None=0*	
Hhducks *(required)*	Ducks<br>*None=0*	
Woman of the Household (1)		(Repeated group)
Woman of the Household (1) > INFORMED CONSENT		
WOMconsent	"Good morning/afternoon/evening. My name is ________. I am representing the GHANA ESSENTIAL HEALTH INTERVENTION PROJECT. We are conducting a survey to assess maternal and child health and we are collecting information on households in this, and other similar communities in Ghana. Your household has been selected to participate in this survey by means of a random or chance selection process, much like taking a cup of beans from a bowl. You have been selected for interview because your household has been selected. I would like to ask you a few questions about you and your child if I may, but you can refuse to answer any question I ask. Your participation in this survey is completely voluntary. You may terminate the interview any time. You can also refuse to participate in the study entirely. The interview will last approximately 1 hour. The information we collect from you will not be shown to anyone outside of this project. If you have any question about this study, you can contact Dr James Akazili on phone number 0244834435 or Mr. Fabian Sebastian Achana on phone number 0243213284<br>*For the first woman you interview IN THIS HOUSEHOLD, you do not need to read the consent form again.*	
WOMconsented *(required)*	Do you agree to be interviewed?<br>*Response constrained to: .=1*	1 Yes<br>2 No
Woman of the Household (1) > SECTION 1: RESPONDENT'S BACKGROUND		
linumber *(required)*	Input number 1 for first woman of this household, 2 for 2nd woman, 3 for 3rd woman, etc<br>*Response constrained to: .<11*	
Woman of the Household (1) > SECTION 1: RESPONDENT'S BACKGROUND > In what month and year were you born?		

Field	Question	Answer
birthmonth	In what month and year were you born?	1 January<br>2 February<br>3 March<br>4 April<br>5 May<br>6 June<br>7 July<br>8 August<br>9 September<br>10 October<br>11 November<br>12 December
birthyear *(required)*	Year<br>*YYYY*<br>*Response constrained to: .>1964 and .<2000*	
edu *(required)*	What is the highest level of formal school you attended?	1 None<br>2 Primary<br>3 JSS<br>4 Middle school<br>5 SSS<br>6 Tertiary<br>other Other
edu_other	Specify other.<br>*Question relevant when: selected(${edu}, 'other')*	
Woman of the Household (1) > SECTION 1: RESPONDENT'S BACKGROUND > In which month and year did you complete that class?		
edu_month *(required)*	In which month and year did you complete that class?<br>*Question relevant when: ${edu} !=1*	1 January<br>2 February<br>3 March<br>4 April<br>5 May<br>6 June<br>7 July<br>8 August<br>9 September<br>10 October<br>11 November<br>12 December
edu_year *(required)*	Year<br>*YYYY*<br>*Question relevant when: ${edu} !=1*<br>*Response constrained to: .>(${birthyear} +5)*	

Field	Question	Answer
literacy *(required)*	Can you read and understand a letter or newspaper in any language?	1 Yes<br>2 No
language	Which language?<br>*Question relevant when: ${literacy} =1*	1 English<br>other Other
language_other	Specify other.<br>*Question relevant when: selected(${language}, 'other')*	
litlevel	Easily or with difficulty?<br>*Question relevant when: ${literacy} =1*	1 Easily<br>2 With difficulty
Woman of the Household (1) > SECTION 1: RESPONDENT'S BACKGROUND > Ethnicity / Religion		
ethnic *(required)*	What is your ethnic group	1 Kassena<br>2 Nankana<br>3 Akan<br>4 Brong<br>5 Nzema<br>6 Ga / Dangme<br>7 Ewe<br>8 Guan<br>9 Buli<br>10 Mamprusi<br>11 Frafra (Grusi, Talensi, Nabdam)<br>12 Kusasi<br>13 Dagbani<br>14 Wali/Dagari<br>15 Sissala<br>other Other
ethnic_other	Specify other.<br>*Question relevant when: selected(${ethnic}, 'other')*	
religion *(required)*	What is your current religion?	1 Christianity<br>2 Traditional religion<br>3 Islam<br>4 No religion<br>other Other

Field	Question	Answer
religion_other	Specify other.<br>*Question relevant when: selected(${religion}, 'other')*	

Woman of the Household (1) > SECTION 1: RESPONDENT'S BACKGROUND > Marital Status

Field	Question	Answer
evermarr *(required)*	Have you ever been married or lived with a man as if married?	1 Yes<br>2 No
marrtimes *(required)*	How many times have you been married or lived with a man as if married?<br>*PROBE FOR NUMBER OF TIMES MARRIED*<br>*Question relevant when: ${evermarr} =1*	
agemarr *(required)*	How old were you when you started living with your FIRST husband/partner?<br>*Question relevant when: ${evermarr} =1*<br>*Response constrained to: .>9 and .<50*	
currmarrstat *(required)*	What is your marital status now?<br>*Question relevant when: ${evermarr} =1*	1 Married<br>2 Widowed<br>3 Divorced<br>4 Separated<br>5 Cohabiting/living together

Woman of the Household (1) > SECTION 1: RESPONDENT'S BACKGROUND > Marital Status > In what month and year did your most recent marriage end?

Field	Question	Answer
marrend_month	In what month and year did your most recent marriage end?<br>*Question relevant when: ${currmarrstat} =2 or ${currmarrstat} =3*	1 January<br>2 February<br>3 March<br>4 April<br>5 May<br>6 June<br>7 July<br>8 August<br>9 September<br>10 October<br>11 November<br>12 December
marrend_year	Year<br>*YYYY*<br>*Question relevant when: ${currmarrstat} =2 or ${currmarrstat} =3*<br>*Response constrained to: .>(${birthyear} +10)*	
spousewithyou	Is your current husband/partner staying with you in this compound?<br>*Question relevant when: ${currmarrstat} =1 or ${currmarrstat} =5*	1 On this compound<br>2 Elsewhere
otherwives *(required)*	Does your current husband/partner have any other wives besides yourself?<br>*Question relevant when: ${currmarrstat} =4 or ${currmarrstat} =1 or ${currmarrstat} =5*	1 Yes<br>2 No<br>3 Don't Know
numotherwives *(required)*	How many other wives does he have?<br>*Question relevant when: ${otherwives} =1*	
wiferank	Are you the first wife, second wife, etc.?<br>*Rank as a number. 1 = first, 2 = second, 3= third, etc.*<br>*Question relevant when: ${otherwives} =1 and ${numotherwives} >1*<br>*Response constrained to: .< ${numotherwives} +2*	

Woman of the Household (1) > SECTION 1: RESPONDENT'S BACKGROUND > Occupation

Field	Question	Answer
occ	What is your occupation, that is, what kind of work do you mainly do?	1 Farming<br>2 Trading/Selling<br>3 Hairdressing/Dressmaking

Field	Question	Answer
		4 Housewife<br>5 Craftmanship<br>6 Construction work<br>7 Civil/Public Servant<br>8 Student<br>9 No occupation<br>other Other
occ_other	Specify other.<br>*Question relevant when: selected(${occ}, 'other')*	
workfreq	Do you usually work throughout the year, or do you work seasonally, or only once in a while?<br>*Question relevant when: ${occ} !=4 and ${occ} !=7 and ${occ} !=8 and ${occ} !=9*	1 Seasonally/Part of the year<br>2 Throughout the year<br>3 Once a while<br>8 NA
worktype	In your current work, do you work for a member of your family, for someone else, or are you self employed?<br>*Question relevant when: ${occ} !=4 and ${occ} !=7 and ${occ} !=8 and ${occ} !=9*	1 For a family member<br>2 For someone else<br>3 Self-Employment
HHpurch *(required)*	Who usually makes decisions about making major household purchases?	1 Respondent<br>2 Husband/partner<br>3 Jointly<br>other Other
HHpurch_other	Specify other.<br>*Question relevant when: selected(${HHpurch}, 'other')*	

Field	Question	Answer
freetovisit *(required)*	Can you visit families and friends without seeking permission?<br>*Question relevant when: ${currmarrstat} =4 or ${currmarrstat} =1 or ${currmarrstat} =5*	1 Yes<br>2 No
spenddecider *(required)*	Who decides how money you earn is spent?<br>*Question relevant when: ${currmarrstat} =4 or ${currmarrstat} =1 or ${currmarrstat} =5*	1 myself only<br>2 partner only<br>3 respondent and partner together<br>4 Other relatives
dailypurchases *(required)*	Who makes decisions about purchases for daily needs?<br>*Question relevant when: ${currmarrstat} =4 or ${currmarrstat} =1 or ${currmarrstat} =5*	1 myself only<br>2 partner only<br>3 respondent and partner together<br>4 Other relatives
refusesex *(required)*	Can you refuse to have sex with your partner without any severe consequences?<br>*Question relevant when: ${currmarrstat} =4 or ${currmarrstat} =1 or ${currmarrstat} =5*	1 Yes<br>2 No
permission *(required)*	Do you need permission to seek care at a health facility?<br>*Question relevant when: ${currmarrstat} =4 or ${currmarrstat} =1 or ${currmarrstat} =5*	1 Yes<br>2 No
Woman of the Household (1) > SECTION 1: RESPONDENT'S BACKGROUND > Cell phone		
owncellphone *(required)*	Do you own a functional cell phone (i.e., the cell phone belongs to you personally rather than your household)?	1 Yes, seen<br>2 Yes, not seen<br>3 No

Field	Question	Answer
anycellphone *(required)*	Do you have access to any cell phone?<br>*Question relevant when: ${owncellphone} =3*	1 Within the household<br>2 Within the compound<br>3 Within the community<br>4 None
cellphonefreq *(required)*	How often do you use your/a cell phone?<br>*Question relevant when: ${anycellphone} !=4*	1 Several times a day<br>2 Daily<br>3 Every other day<br>4 Weekly<br>5 Monthly<br>other Other
cellphonefreq_other	Specify other.<br>*Question relevant when: selected(${cellphonefreq}, 'other')*	
cellphonepurp *(required)*	What do you usually use your/a cell phone for?<br>*Question relevant when: ${owncellphone} !=3 or ${anycellphone} !=4*	1 Make/receive calls<br>2 Send/receive SMS<br>3 Listen to radio<br>4 Internet services<br>5 Listen to music<br>other Other
cellphonepurp_other	Specify other.<br>*Question relevant when: selected(${cellphonepurp}, 'other')*	
callpurp *(required)*	When you make calls, what are the calls usually for?<br>*Question relevant when: ${cellphonepurp} =1*	1 Trading/Marketing<br>2 Other ways to earn money<br>3 Social call to or from friends<br>4 Calls to or from husband, children<br>5 Calls to or from other relatives<br>6 Calls to health worker or another person regarding the health of a family member<br>other Other
callpurp_other	Specify other.<br>*Question relevant when: selected(${callpurp}, 'other')*	

Field	Question	Answer
callwhom *(required)*	When you make a call when you yourself or a family member has a health concern, who do you call?<br>*Question relevant when: ${callpurp} =6*	1 Mother<br>2 Sister<br>3 Other female relative<br>4 Father<br>5 Husband<br>6 Other male relative<br>7 IMCI Volunteer<br>8 CHO<br>9 Other health worker<br>10 Other person<br>other Other
callwhom_other	Specify other.<br>*Question relevant when: selected(${callwhom}, 'other')*	
textwhom *(required)*	Over the last 7 days, who did you send/receive SMS to/from?<br>*Question relevant when: ${cellphonepurp} =2*	1 Mother-in-law<br>2 Father-in-law<br>3 Husband<br>4 Mother/father<br>5 Other relative<br>6 Friend<br>7 Health worker<br>8 Other person<br>other Other
textwhom_other	Specify other.<br>*Question relevant when: selected(${textwhom}, 'other')*	
callwhom7	Over the last 7 days, who did you send/receive phone calls to/from?<br>*Question relevant when: ${cellphonepurp} =2*	1 Mother-in-law<br>2 Father-in-law<br>3 Husband<br>4 Mother/father<br>5 Other relative<br>6 Friend<br>7 Health worker<br>8 Other person

Field	Question	Answer
		other Other
callwhom7_other	Specify other.<br>*Question relevant when: selected(${callwhom7}, 'other')*	
callHW	Have you ever called a health worker about a health concern about you or a member of your family?<br>*Question relevant when: ${cellphonepurp} =1*	1 Yes<br>2 No
lastcallwhom	On the last occasion when you made such a call, who did you call?<br>*Question relevant when: ${callHW} =1*	1 CHO<br>2 Doctor<br>3 Mid-wife<br>4 Trained TBA<br>5 Untrained TBA<br>6 Community Health Volunteer<br>other Other
lastcallwhom_other	Specify other.<br>*Question relevant when: selected(${lastcallwhom}, 'other')*	
callHWreason	What was the reason for which you contacted the health worker?<br>*Question relevant when: ${callHW} =1*	1 Emergency health care<br>2 Health counseling/advice<br>other Other
callHWreason_other	Specify other.<br>*Question relevant when: selected(${callHWreason}, 'other')*	

Woman of the Household (1) > SECTION 1: RESPONDENT'S BACKGROUND > Media

Field	Question	Answer
TV *(required)*	Do you watch television almost every day, at least once a week, less than once a week, or not at all?	1 Everyday<br>2 Almost every day<br>3 At least once a week<br>4 Less than once a week<br>5 Not at all
radio *(required)*	Do you listen to the radio almost every day, at least once a week, less than once a week, or not at all?	1 Everyday<br>2 Almost every day<br>3 At least once a week<br>4 Less than once a week<br>5 Not at all

Woman of the Household (1) > SECTION 1: RESPONDENT'S BACKGROUND > Insurance

Field	Question	Answer
NHIS *(required)*	Have you ever registered as a member of the National Health Insurance Scheme (NHIS)?	1 Yes<br>2 No
validNHIS *(required)*	Do you currently have a valid NHIS card?<br>*Please ask to see NHIS card and verify if member is currently active*<br>*Question relevant when: ${NHIS} =1*	1 Yes, seen and valid<br>2 Yes, seen and not valid<br>3 Yes, not seen<br>4 No NHIS card

Woman of the Household (1) > SECTION 2: REPRODUCTION

Field	Question	Answer
numbirths *(required)*	Now I would like to ask you how many children you have given birth to.<br>*None = 0*<br>*Response constrained to: .>=0*	

Field	Question	Answer
livewithkids *(required)*	Do you have any sons or daughters who are now living with you?<br>*Question relevant when: ${numbirths} !=0*<br>*Response constrained to: .=1*	1 Yes<br>2 No

Woman of the Household (1) > SECTION 2: REPRODUCTION > How many sons and daughters live WITH you?

Field	Question	Answer
livewithsons *(required)*	Sons<br>*Question relevant when: ${numbirths} !=0 and ${livewithkids} =1*<br>*Response constrained to: .<= ${numbirths}*	
livewithdaughters *(required)*	Daughters<br>*Question relevant when: ${numbirths} !=0 and ${livewithkids} =1*<br>*Response constrained to: .<= ${numbirths} - ${livewithsons}*	
liveelsekids *(required)*	Do you have any sons or daughters who are alive but do not live with you?<br>*Question relevant when: ${numbirths} !=0*<br>*Response constrained to: .=1*	1 Yes<br>2 No

Woman of the Household (1) > SECTION 2: REPRODUCTION > How many sons and daughters are ALIVE but DO NOT LIVE WITH you?

Field	Question	Answer
liveelsesons *(required)*	Sons<br>*Question relevant when: ${numbirths} !=0 and ${liveelsekids} =1*<br>*Response constrained to: .<= ${numbirths} - ${livewithsons} - ${livewithdaughters}*	

Field	Question	Answer
liveelsedaughters *(required)*	Daughters<br>*Question relevant when: ${numbirths} !=0 and ${liveelsekids} =1*<br>*Response constrained to: .<= ${numbirths} - ${livewithsons} - ${livewithdaughters} - ${liveelsesons}*	
deadkids *(required)*	Have you ever given birth to a son or a daughter who was born alive but later died?<br>*IF NO, PROBE: Any baby who cried or showed any sign of life but only survived a few hours or days?*<br>*Question relevant when: ${numbirths} !=0*<br>*Response constrained to: .=1*	1 Yes<br>2 No
Woman of the Household (1) > SECTION 2: REPRODUCTION > How many sons and daughters have died?		
deadsons *(required)*	Sons<br>*Question relevant when: ${numbirths} !=0 and ${deadkids} =1*<br>*Response constrained to: .<= ${numbirths} - ${livewithsons} - ${livewithdaughters} - ${liveelsesons} - ${liveelsedaughters}*	
deaddaughters *(required)*	Daughters<br>*Question relevant when: ${numbirths} !=0 and ${deadkids} =1*<br>*Response constrained to: .<= ${numbirths} - ${livewithsons} - ${livewithdaughters} - ${liveelsesons} - ${liveelsedaughters} - ${deadsons}*	
miscarry *(required)*	Women sometimes have pregnancies that do not result in a live born child. That is, a pregnancy can end early, in a miscarriage, or the child can be born dead. Have you had any such pregnancy that did not result in a live birth?	1 Yes<br>2 No
numlostpreg *(required)*	In all, how many pregnancies - that did not end in a live born child - have you had?<br>*Question relevant when: ${miscarry} =1*	
currpreg *(required)*	Are you pregnant now?	1 Yes<br>2 No<br>9 Don't know<br>8 Not applicable
currpregdur	How long, in months, have you been pregnant?<br>*Question relevant when: ${currpreg} =1*	
pregcheck1	Just to make sure that I have this right, you have had [TPpregnomiss] TOTAL PREGNANCIES during your life. Is that correct?<br>*If [TPpregnomiss] pregnancies is not correct, PROBE.*<br>*Question relevant when: ${currpreg} =1 and ${miscarry} !=1*<br>*Response constrained to: .=1*	1 Yes<br>2 No

Field	Question	Answer
pregcheck2	Just to make sure that I have this right, you have had [TPpregmiss] TOTAL PREGNANCIES during your life. Is that correct?<br>*If [TPpregmiss] pregnancies is not correct, PROBE.*<br>*Question relevant when: ${currpreg} =1 and ${miscarry} =1*<br>*Response constrained to: .=1*	1 Yes<br>2 No
pregcheck3	Just to make sure that I have this right, you have had [TPnotpregnomiss] TOTAL PREGNANCIES during your life. Is that correct?<br>*If [TPnotpregnomiss] pregnancies is not correct, PROBE.*<br>*Question relevant when: ${currpreg} !=1 and ${miscarry} !=1*<br>*Response constrained to: .=1*	1 Yes<br>2 No
pregcheck4	Just to make sure that I have this right, you have had [TPnotpregmiss] TOTAL PREGNANCIES during your life. Is that correct?<br>*If [TPnotpregmiss] pregnancies is not correct, PROBE.*<br>*Question relevant when: ${currpreg} !=1 and ${miscarry} =1*<br>*Response constrained to: .=1*	1 Yes<br>2 No
preghist	Now I would like to ask you details about your pregnancies, whether born alive, born dead, or lost before full term. Record all pregnancies, starting with the most recent of your pregnancies, including current pregnancy if pregnant.<br>*Question relevant when: ${numbirths} >0 or ${numlostpreg} >0 or ${currpreg} =1*	

Woman of the Household (1) > SECTION 2: REPRODUCTION > For your current pregnancy:

Group relevant when: ${currpreg} =1

Field	Question	Answer
pregintent_currpreg *(required)*	You said you are currently pregnant. When you got pregnant with this pregnancy, did you want to get pregnant at that time?	1 Yes<br>2 No<br>3 Don't Know
pregtiming_currpreg *(required)*	Did you want to have a baby later on or did you not want any more children?<br>*Question relevant when: ${pregintent_currpreg} !=1*	1 Later<br>2 No more<br>8 NA
wantedwait_currpreg	How much longer did you want to wait?<br>*Question relevant when: ${pregintent_currpreg} !=1 and ${pregtiming_currpreg} !=2*	1 Months<br>2 Years<br>8 NA
wantedwait_mo_currpreg	Months<br>*Question relevant when: ${wantedwait_currpreg} =1*	
wantedwait_yr_currpreg	Years<br>*Question relevant when: ${wantedwait_currpreg} =2*	
ANCcheck_currpreg	Did you see anyone for antenatal care during this pregnancy?	1 Yes<br>2 No
ANCprov_currpreg *(required)*	Whom did you see?<br>*Question relevant when: ${ANCcheck_currpreg} =1*	1 Doctor<br>2 Midwife<br>3 Nurse<br>4 Community health officer<br>5 Health extension worker<br>6 Traditional birth attendant<br>7 Other health worker<br>other Other

Field	Question	Answer
ANCprov_currpreg_other	Specify other.<br>*Question relevant when: selected(${ANCprov_currpreg}, 'other')*	
ANCplace_currpreg *(required)*	Where did you receive antenatal care for this pregnancy? Anywhere else?<br>*Probe to identify type(s) of source(s) and circle the appropriate code(s). If there is no appropriate code, select 'other', then enter the name of the place.*<br>*Question relevant when: ${ANCcheck_currpreg} =1*	1 Govt. Hospital<br>2 Govt. Health center/Clinic<br>3 CHPS compound<br>4 NGO facility<br>5 Private Clinic<br>6 Home<br>other Other
ANCplace_currpreg_other	Specify other.<br>*Question relevant when: selected(${ANCplace_currpreg}, 'other')*	
pinkbook_currpreg *(required)*	Do you have a maternal health book (pink book) for this pregnancy?<br>*Question relevant when: ${ANCcheck_currpreg} =1*	1 Yes, seen<br>2 Yes, not seen<br>3 No<br>8 NA
pinkbookANCvisits_currpreg	Enter number of visits from health book<br>*Question relevant when: ${ANCcheck_currpreg} =1 and ${pinkbook_currpreg} =1*	
ANC_mo_currpreg *(required)*	How many months pregnant were you when you first received antenatal care for this pregnancy?<br>*Question relevant when: ${ANCcheck_currpreg} =1*<br>*Response constrained to: .<= ${currpregdur}*	
firstprov_currpreg	Who did you first receive antenatal care from during this pregnancy?<br>*Question relevant when: ${ANCcheck_currpreg} =1*	1 Doctor<br>2 Midwife<br>3 Nurse<br>4 Community health officer<br>5 Health extension worker<br>6 Traditional birth attendant<br>7 Other health worker
firstplace_currpreg	Where did you first receive antenatal care from during this pregnancy?<br>*Question relevant when: ${ANCcheck_currpreg} =1*	1 Govt. Hospital<br>2 Govt. Health center/Clinic<br>3 CHPS compound<br>4 NGO facility

Field	Question	Answer
		5 Private Clinic<br>6 Home<br>other Other
firstplace_currpreg_other	Specify other.<br>*Question relevant when: selected(${firstplace_currpreg}, 'other')*	
srANCvisits_currpreg *(required)*	How many times in total did you receive antenatal care during this pregnancy?<br>*Question relevant when: ${ANCcheck_currpreg} =1*<br>*Response constrained to: .<21*	
mismatch_currpreg *(required)*	I notice that you mentioned receiving a number of ANC visits that is different than are listed in your book. Sometimes women may forget to bring their book to ANC; sometime the health worker may also not write in your book? Did this happen to you?<br>*Multiple answers allowed*<br>*Question relevant when: ${pinkbook_currpreg} =1 and (${pinkbookANCvisits_currpreg} != ${srANCvisits_currpreg})*	1 Forgot to bring book<br>2 HW did not write<br>3 Other reason<br>other Other
mismatch_currpreg_other	Specify other.<br>*Question relevant when: selected(${mismatch_currpreg}, 'other')*	

Woman of the Household (1) > SECTION 2: REPRODUCTION > For your current pregnancy: > duringpreg_currpreg

Group relevant when: ${ANCcheck_currpreg} =1

Field	Question	Answer
generated_table_list_label_203	During this pregnancy:	
reserved_name_for_field_list_labels_204		1 Yes<br>2 No

Field	Question	Answer
pregweight_currpreg *(required)*	Were you weighed?<br>*Question relevant when: ${ANCcheck_currpreg} =1*	1 Yes<br>2 No
pregBP_currpreg *(required)*	Was your blood pressure measured?<br>*Question relevant when: ${ANCcheck_currpreg} =1*	1 Yes<br>2 No
pregheight_currpreg *(required)*	Was your height measured?<br>*Question relevant when: ${ANCcheck_currpreg} =1*	1 Yes<br>2 No
preguring_currpreg *(required)*	Did you give a urine sample?<br>*Question relevant when: ${ANCcheck_currpreg} =1*	1 Yes<br>2 No
pregblood_currpreg *(required)*	Did you give a blood sample?<br>*Question relevant when: ${ANCcheck_currpreg} =1*	1 Yes<br>2 No
pregstomach_currpreg *(required)*	Was your stomach measured?<br>*Question relevant when: ${ANCcheck_currpreg} =1*	1 Yes<br>2 No
pregHR_currpreg *(required)*	Did a health worker listen to the heart rate of your baby?<br>*Question relevant when: ${ANCcheck_currpreg} =1*	1 Yes<br>2 No

Field	Question	Answer
Woman of the Household (1) > SECTION 2: REPRODUCTION > For your current pregnancy: > duringpregHW_currpreg<br>*Group relevant when: ${ANCcheck_currpreg} =1*		
generated_table_list_label_212	During this pregnancy, were you counseled by a health worker on:	
reserved_name_for_field_list_labels_213		1 Yes<br>2 No
pregfinprep_currpreg *(required)*	Financial preparation for your delivery?<br>*Question relevant when: ${ANCcheck_currpreg} =1*	1 Yes<br>2 No
pregbf_currpreg *(required)*	Breastfeeding immediately after delivery?<br>*Question relevant when: ${ANCcheck_currpreg} =1*	1 Yes<br>2 No
pregtt_currpreg *(required)*	Tetanus toxic vaccination?<br>*Question relevant when: ${ANCcheck_currpreg} =1*	1 Yes<br>2 No
pregdanger_currpreg *(required)*	Danger signs during delivery?<br>*Question relevant when: ${ANCcheck_currpreg} =1*	1 Yes<br>2 No
pregwrap_currpreg *(required)*	Wrapping the newborn after delivery?<br>*Question relevant when: ${ANCcheck_currpreg} =1*	1 Yes<br>2 No

Field	Question	Answer
pregSBA_currpreg *(required)*	Using a skilled birth attendant?<br>*Question relevant when: ${ANCcheck_currpreg} =1*	1 Yes<br>2 No
pregFP_currpreg *(required)*	Family planning?<br>*Question relevant when: ${ANCcheck_currpreg} =1*	1 Yes<br>2 No
pregtransport_currpreg *(required)*	Identifying emergency transport options?<br>*Question relevant when: ${ANCcheck_currpreg} =1*	1 Yes<br>2 No
pregdiet_currpreg *(required)*	Diet?<br>*Question relevant when: ${ANCcheck_currpreg} =1*	1 Yes<br>2 No
pregother_currpreg *(required)*	Other<br>*Question relevant when: ${ANCcheck_currpreg} =1*	1 Yes<br>2 No
other_currpreg	Please specify other things you were counseled on<br>*Question relevant when: ${pregother_currpreg} =1*	
dangersigns_currpreg	During (any of) your antenatal care visit(s), were you told about the things to look out for that might suggest problems with the pregnancy?<br>*Question relevant when: ${ANCcheck_currpreg} =1*	1 Yes<br>2 No

Field	Question	Answer
dangerplace_currpreg	Were you told where to go if you had any of these complications?<br>*Question relevant when: ${ANCcheck_currpreg} =1 and ${dangersigns_currpreg} =1*	1 Yes<br>2 No
pregbednet_currpreg	At the time of this pregnancy, did your household have any bed net?	1 Yes<br>2 No<br>3 Don't Know
sleepfreq_currpreg	How often have you slept under a bed net during this pregnancy?<br>*Question relevant when: ${pregbednet_currpreg} =1*	1 Every night<br>2 Most nights<br>3 Some nights<br>4 Rarely<br>5 Never<br>8 NA
pregmalaria_currpreg *(required)*	During this pregnancy, have you taken any drugs to keep you from getting malaria?	1 Yes<br>2 No
pregmalariameds_currpreg *(required)*	What drugs have you taken?<br>*Question relevant when: ${pregmalaria_currpreg} =1*	1 SP<br>2 ACT<br>8 NA<br>9 DK<br>other Other
pregmalariameds_currpreg_other	Specify other.<br>*Question relevant when: selected(${pregmalariameds_currpreg}, 'other')*	
SPtimes_currpreg	How many times have you taken SP?<br>*Enter 99 for DK*<br>*Question relevant when: ${pregmalariameds_currpreg} =1*	
ACTtimes_currpreg	How many times have you taken ACT?<br>*Enter 99 for DK*<br>*Question relevant when: ${pregmalariameds_currpreg} =2*	

Field	Question	Answer
Woman of the Household (1) > SECTION 2: REPRODUCTION > For your current pregnancy: > SPandACT_cur<br>*Group relevant when: ${pregmalariameds_currpreg} =1 and ${pregmalariameds_currpreg} =2*		
generated_table_list_label_233	How many times have you taken each of the following during this pregnancy	
SPandACT_SP_cur *(required)*	SP_cur<br>*Response constrained to: .>0*	
SPandACT_ACT_cur *(required)*	ACT_cur<br>*Response constrained to: .>0*	
drugs_currpreg	Did you get the drugs during any antenatal care visit, during another visit to a health facility or from another source?<br>*Question relevant when: ${pregmalariameds_currpreg} =1*	1 ANC Visit<br>2 Other health facility visit<br>3 Other source<br>8 NA
tetanus_currpreg *(required)*	During this pregnancy have you been given an injection in the arm to prevent you and the baby from getting tetanus?	1 Yes<br>2 No
tetanustimes_currpreg *(required)*	During this pregnancy, how many times have you received this tetanus injection?<br>*DK = 99*<br>*Question relevant when: ${tetanus_currpreg} =1*<br>*Response constrained to: .>0 and .<3 or .=99*	

Field	Question	Answer
previoustet_currpreg	At any time before this pregnancy, did you receive any tetanus injections?	1 Yes<br>2 No<br>9 Don't know<br>8 Not applicable
prevtettimes_currpreg	Before this pregnancy, how many tetanus injections did you receive in total?<br>*Question relevant when: ${previoustet_currpreg} =1*	
prevtetyr_currpreg	How many years ago did you receive the last tetanus injection before this pregnancy?<br>*Question relevant when: ${previoustet_currpreg} =1*	
pregworm_currpreg	During this pregnancy, have you taken any drug for intestinal worms?	1 Yes<br>2 No
pregprep_currpreg *(required)*	During this pregnancy, have you made any preparations for delivery?	1 Yes<br>2 No
Woman of the Household (1) > SECTION 2: REPRODUCTION > For your current pregnancy: > preparation_currpreg<br>*Group relevant when: ${pregprep_currpreg} =1*		
generated_table_list_label_247	What kind of preparation have you made?<br>*Include all responses which the mother mentions unprompted. Then ask, "Is there anything else." Then, read each question and select "yes" or "no."*	
reserved_name_for_field_list_labels_248		1 Yes<br>2 No
preptransport_currpreg *(required)*	Transport<br>*Question relevant when: ${pregprep_currpreg} =1*	1 Yes<br>2 No

Field	Question	Answer
prepmoney_currpreg *(required)*	Money<br>*Question relevant when: ${pregprep_currpreg} =1*	1 Yes<br>2 No
prepfood_currpreg *(required)*	Food<br>*Question relevant when: ${pregprep_currpreg} =1*	1 Yes<br>2 No
prepinstruments_currpreg *(required)*	Clean instruments for delivery<br>*Question relevant when: ${pregprep_currpreg} =1*	1 Yes<br>2 No
prepcloths_currpreg *(required)*	Clean cloths<br>*Question relevant when: ${pregprep_currpreg} =1*	1 Yes<br>2 No
prepother_currpreg *(required)*	Other<br>*Question relevant when: ${pregprep_currpreg} =1*	1 Yes<br>2 No
delplan_currpreg *(required)*	Have you discussed planning for your delivery with anybody for this pregnancy?	1 Yes<br>2 No
delplanwhom_currpreg	Whom did you plan your delivery with?<br>*Question relevant when: ${delplan_currpreg} =1*	1 Husband<br>2 Mother-in-law<br>3 Father-in-law<br>4 Friends/relative<br>5 CHN/CHO<br>6 Community Health volunteer<br>7 Other health worker<br>8 NA<br>9 DK<br>other Other
delplanwhom_currpreg_other	Specify other.<br>*Question relevant when: selected(${delplanwhom_currpreg}, 'other')*	

Field	Question	Answer
facdel_currpreg *(required)*	Have any health worker given you specific instructions to go deliver at a health facility?	1 NONE<br>2 Doctor<br>3 Midwife<br>4 Nurse<br>5 Community health officer<br>6 Health extension worker<br>other Other
facdel_currpreg_other	Specify other.<br>*Question relevant when: selected(${facdel_currpreg}, 'other')*	
whenrec_currpreg	When did they make this recommendation?<br>*Question relevant when: ${facdel_currpreg} >3*	1 1st trimester<br>2 2nd trimester<br>3 3rd trimester, but before labor started<br>4 3rd trimester, during labor<br>8 NA<br>9 Don't know<br>other Other
whenrec_currpreg_other	Specify other.<br>*Question relevant when: selected(${whenrec_currpreg}, 'other')*	
whyrec_currpreg	Why did they make this recommendation?<br>*Question relevant when: ${facdel_currpreg} >3*	1 Does not know why<br>2 Suspected twins<br>3 Position of the baby<br>4 Hypertension/edema /blurred vision<br>5 Previous c-section<br>6 First birth<br>7 Bleeding<br>8 Many hours in labor<br>9 Lack of movement of fetus<br>10 Diabetes<br>11 Anemia<br>88 NA<br>other Other
whyrec_currpreg_other	Specify other.<br>*Question relevant when: selected(${whyrec_currpreg}, 'other')*	

Field	Question	Answer
otherrec_currpreg	Did anyone else recommend that you go to deliver at health facility?	1 Husband<br>2 Mother-in-law<br>3 Mother<br>4 Friends/relative<br>5 No one else<br>other Other
otherrec_currpreg_other	Specify other.<br>*Question relevant when: selected(${otherrec_currpreg}, 'other')*	
delinstruct_currpreg	Have any health worker given you specific instructions to call him/her at the time of delivery?	1 NONE<br>2 Doctor<br>3 Midwife<br>4 Nurse<br>5 Community health officer<br>6 Health extension worker<br>other Other
delinstruct_currpreg_other	Specify other.<br>*Question relevant when: selected(${delinstruct_currpreg}, 'other')*	
Woman of the Household (1) > SECTION 2: REPRODUCTION > PREGNANCY HISTORY (1)<br>*Group relevant when: ${numbirths} >0 or ${numlostpreg} >0*		(Repeated group)
p3 *(required)*	Was the baby born alive, born dead or lost before birth?	1 Alive<br>2 Dead<br>3 Lost
p4 *(required)*	Did that baby cry, move or breathe when it was born?<br>*Question relevant when: ${p3} =2*	1 Yes<br>2 No
p6a *(required)*	Is this child still alive?<br>*Question relevant when: ${p3} =1*	1 Yes<br>2 No

Field	Question	Answer

Woman of the Household (1) > SECTION 2: REPRODUCTION > PREGNANCY HISTORY (1) > Born alive and still living

Group relevant when: ${p3} =1 and ${p6a} =1

Field	Question	Answer
multiple_alive *(required)*	Was this a multiple birth?<br>*For multiple births, add the other birth separately as a new group with same month and year of birth and this birth*	1 singleton<br>2 twin<br>3 triplets<br>4 quadruplets
p2a_alive	How many months did this pregnancy last?<br>*Response constrained to: .<13*	
p5_name_alive *(required)*	What is this child's name?	
p6_alive *(required)*	Is [p5_name_alive] a boy or a girl?	1 Male<br>2 Female

Woman of the Household (1) > SECTION 2: REPRODUCTION > PREGNANCY HISTORY (1) > Born alive and still living > In what month and year did you give birth to [p5_name_alive]?

Field	Question	Answer
p2b_mo_alive *(required)*	In what month and year did you give birth to [p5_name_alive]?	1 January<br>2 February<br>3 March<br>4 April<br>5 May<br>6 June<br>7 July<br>8 August<br>9 September<br>10 October<br>11 November<br>12 December
p2b_yr_alive *(required)*	Year<br>*YYYY*<br>*Response constrained to: .>=(${birthyear} +12)*	

Field	Question	Answer
p7_yr_alive	How old was [p5_name_alive] at his/her last birthday?<br>*Enter age in years. If child was less than 1 year old, enter 0 here and enter months in the next question*<br>*Response constrained to: .<= ${U5_alive}*	
p7_mo_alive	Months<br>*Question relevant when: ${p7_yr_alive} =0*<br>*Response constrained to: .<12*	
p8_alive	Is [p5_name_alive] living with you?	1 Yes<br>2 No
Woman of the Household (1) > SECTION 2: REPRODUCTION > PREGNANCY HISTORY (1) > Born alive and still living > ANC for [p5_name_alive]<br>*Group relevant when: ${U5_alive} <60*		
pregintent_alive *(required)*	When you got pregnant with [p5_name_alive], did you want to get pregnant at that time?	1 Yes<br>2 No<br>3 Don't Know
pregtiming_alive *(required)*	Did you want to have a baby later on or did you not want any more children?<br>*Question relevant when: ${pregintent_alive} !=1*	1 Later<br>2 No more<br>8 NA
wantedwait_alive *(required)*	How much longer did you want to wait?<br>*Question relevant when: ${pregintent_alive} !=1 and ${pregtiming_alive} !=2*	1 Months<br>2 Years<br>8 NA
wantedwait_mo_alive *(required)*	Months<br>*Question relevant when: ${wantedwait_alive} =1*	
wantedwait_yr_alive *(required)*	Years<br>*Question relevant when: ${wantedwait_alive} =2*	

Field	Question	Answer
ANCcheck_alive *(required)*	Did you see anyone for antenatal care during this pregnancy?	1 Yes<br>2 No
ANCprov_alive	Whom did you see?<br>*Question relevant when: ${ANCcheck_alive} =1*	1 Doctor<br>2 Midwife<br>3 Nurse<br>4 Community health officer<br>5 Health extension worker<br>6 Traditional birth attendant<br>7 Other health worker<br>other Other
ANCprov_alive_other	Specify other.<br>*Question relevant when: selected(${ANCprov_alive}, 'other')*	
ANCplace_alive	Where did you receive antenatal care for this pregnancy? Anywhere else?<br>*Probe to identify type(s) of source(s) and circle the appropriate code(s). If there is no appropriate code, select 'other', then enter the name of the place.*<br>*Question relevant when: ${ANCcheck_alive} =1*	1 Govt. Hospital<br>2 Govt. Health center/Clinic<br>3 CHPS compound<br>4 NGO facility<br>5 Private Clinic<br>6 Home<br>other Other
ANCplace_alive_other	Specify other.<br>*Question relevant when: selected(${ANCplace_alive}, 'other')*	
pinkbook_alive *(required)*	Did you ever have a maternal health book (pink book) for this pregnancy?<br>*Question relevant when: ${ANCcheck_alive} =1*	1 Yes, seen<br>2 Yes, not seen<br>3 No<br>8 NA
pinkbookANCvisits_alive	Enter number of visits from health book<br>*Question relevant when: ${pinkbook_alive} =1*	
ANC_mo_alive	How many months pregnant were you when you first received antenatal care for this pregnancy?<br>*Question relevant when: ${ANCcheck_alive} =1*<br>*Response constrained to: .<= ${p2a_alive}*	
firstprov_alive *(required)*	Who did you first receive antenatal care from during your pregnancy?<br>*Question relevant when: ${ANCcheck_alive} =1*	1 Doctor<br>2 Midwife

Field	Question	Answer
		3 Nurse<br>4 Community health officer<br>5 Health extension worker<br>6 Traditional birth attendant<br>7 Other health worker
firstplace_alive *(required)*	Where did you first receive antenatal care from during your pregnancy?<br>*Question relevant when: ${ANCcheck_alive} =1*	1 Govt. Hospital<br>2 Govt. Health center/Clinic<br>3 CHPS compound<br>4 NGO facility<br>5 Private Clinic<br>6 Home<br>other Other
firstplace_alive_other	Specify other.<br>*Question relevant when: selected(${firstplace_alive}, 'other')*	
srANCvisits_alive	How many times in total did you receive antenatal care during this pregnancy?<br>*99=DK*<br>*Question relevant when: ${ANCcheck_alive} =1*	
mismatch_alive	I notice that you mentioned receiving a number of ANC visits that is different than are listed in your book. Sometimes women may forget to bring their book to ANC; sometime the health worker may also not write in your book? Did this happen to you?<br>*Multiple answers allowed*<br>*Question relevant when: ${pinkbook_alive} =1 and ${pinkbookANCvisits_alive} != ${srANCvisits_alive}*	1 Forgot to bring book<br>2 HW did not write<br>3 Other reason<br>other Other
mismatch_alive_other	Specify other.<br>*Question relevant when: selected(${mismatch_alive}, 'other')*	
Woman of the Household (1) > SECTION 2: REPRODUCTION > PREGNANCY HISTORY (1) > Born alive and still living > ANC for [p5_name_alive] > duringpreg_alive<br>*Group relevant when: ${ANCcheck_alive} =1*		
generated_table_list_label_302	During this pregnancy:	
reserved_name_for_field_list_labels_303		1 Yes<br>2 No
pregweight_alive *(required)*	Were you weighed?<br>*Question relevant when: ${ANCcheck_alive} =1*	1 Yes<br>2 No
pregBP_alive *(required)*	Was your blood pressure measured?<br>*Question relevant when: ${ANCcheck_alive} =1*	1 Yes<br>2 No
pregheight_alive *(required)*	Was your height measured?<br>*Question relevant when: ${ANCcheck_alive} =1*	1 Yes<br>2 No
preguring_alive *(required)*	Did you give a urine sample?<br>*Question relevant when: ${ANCcheck_alive} =1*	1 Yes<br>2 No
pregblood_alive *(required)*	Did you give a blood sample?<br>*Question relevant when: ${ANCcheck_alive} =1*	1 Yes<br>2 No
pregstomach_alive *(required)*	Was your stomach measured?<br>*Question relevant when: ${ANCcheck_alive} =1*	1 Yes<br>2 No
pregHR_alive *(required)*	Did a health worker listen to the heart rate of your baby?<br>*Question relevant when: ${ANCcheck_alive} =1*	1 Yes<br>2 No
Woman of the Household (1) > SECTION 2: REPRODUCTION > PREGNANCY HISTORY (1) > Born alive and still living > ANC for [p5_name_alive] > duringpregHW_alive<br>*Group relevant when: ${ANCcheck_alive} =1*		
generated_table_list_label_311	During this pregnancy, were you counseled by a health worker on:	

Field	Question	Answer
reserved_name_for_field_list_labels_312		1 Yes<br>2 No
pregfinprep_alive *(required)*	Financial preparation for your delivery?<br>*Question relevant when: ${ANCcheck_alive} =1*	1 Yes<br>2 No
pregbf_alive *(required)*	Breastfeeding immediately after delivery?<br>*Question relevant when: ${ANCcheck_alive} =1*	1 Yes<br>2 No
pregtt_alive *(required)*	Tetanus toxic vaccination?<br>*Question relevant when: ${ANCcheck_alive} =1*	1 Yes<br>2 No
pregdanger_alive *(required)*	Danger signs during delivery?<br>*Question relevant when: ${ANCcheck_alive} =1*	1 Yes<br>2 No
pregwrap_alive *(required)*	Wrapping the newborn after delivery?<br>*Question relevant when: ${ANCcheck_alive} =1*	1 Yes<br>2 No
pregSBA_alive *(required)*	Using a skilled birth attendant?<br>*Question relevant when: ${ANCcheck_alive} =1*	1 Yes<br>2 No
pregFP_alive *(required)*	Family planning?<br>*Question relevant when: ${ANCcheck_alive} =1*	1 Yes<br>2 No

Field	Question	Answer
pregtransport_alive *(required)*	Identifying emergency transport options? *Question relevant when: ${ANCcheck_alive} =1*	1 Yes<br>2 No
pregdiet_alive *(required)*	Diet? *Question relevant when: ${ANCcheck_alive} =1*	1 Yes<br>2 No
pregother_alive *(required)*	Other *Question relevant when: ${ANCcheck_alive} =1*	1 Yes<br>2 No
other_alive	Please specify other things you were counseled on *Question relevant when: ${pregother_alive} =1*	
dangersigns_alive *(required)*	During (any of) your antenatal care visit(s) with [p5_name_alive], were you told about the things to look out for that might suggest problems with the pregnancy? *Question relevant when: ${ANCcheck_alive} =1*	1 Yes<br>2 No
dangerplace_alive *(required)*	Were you told where to go if you had any of these complications? *Question relevant when: ${ANCcheck_alive} =1 and ${dangersigns_alive} =1*	1 Yes<br>2 No
pregbednet_alive *(required)*	At the time of this pregnancy, did your household have any bed net?	1 Yes<br>2 No<br>3 Don't Know
sleepfreq_alive *(required)*	How often did you sleep under a bed net during this pregnancy? *Question relevant when: ${pregbednet_alive} !=2*	1 Every night<br>2 Most nights<br>3 Some nights<br>4 Rarely<br>5 Never<br>8 NA
pregmalaria_alive *(required)*	During this pregnancy, did you take any drugs to keep you from getting malaria?	1 Yes<br>2 No
pregmalariameds_alive	What drugs did you take? *Question relevant when: ${pregmalaria_alive} =1*	1 SP<br>2 ACT<br>8 NA<br>9 DK<br>other Other
pregmalariameds_alive_other	Specify other. *Question relevant when: selected(${pregmalariameds_alive}, 'other')*	
SPtimes_alive	How many times did you take SP? *Enter 99 for DK* *Question relevant when: ${pregmalariameds_alive} =1*	

Field	Question	Answer
ACTtimes_alive	How many times did you take ACT?<br>*Enter 99 for DK*<br>*Question relevant when: ${pregmalariameds_alive} =2*	

Woman of the Household (1) > SECTION 2: REPRODUCTION > PREGNANCY HISTORY (1) > Born alive and still living > ANC for [p5_name_alive] > SPandACT_alive

Group relevant when: ${pregmalariameds_alive} =1 and ${pregmalariameds_alive} =2

Field	Question	Answer
generated_table_list_label_332	How many times have you taken each of the following during this pregnancy	
SPandACT_SP_alive *(required)*	SP_alive<br>*Response constrained to: .>0*	
SPandACT_ACT_alive *(required)*	ACT_alive<br>*Response constrained to: .>0*	

Field	Question	Answer
drugs_alive *(required)*	Did you get the drugs during any antenatal care visit, during another visit to a health facility or from another source?<br>*Question relevant when: ${pregmalariameds_alive} =1*	1 ANC Visit<br>2 Other health facility visit<br>3 Other source<br>8 NA
tetanus_alive *(required)*	During this pregnancy were you given an injection in the arm to prevent you and the baby from getting tetanus?	1 Yes<br>2 No
tetanustimes_alive *(required)*	During this pregnancy, how many times did you get this tetanus injection?<br>*Question relevant when: ${tetanus_alive} =1*	
previoustet_alive *(required)*	At any time before this pregnancy, did you receive any tetanus injections?	1 Yes<br>2 No<br>9 Don't know<br>8 Not applicable
prevtettimes_alive *(required)*	Before this pregnancy, how many tetanus injections did you receive in total?<br>*Question relevant when: ${previoustet_alive} =1*	

Field	Question	Answer
prevtetyr_alive *(required)*	How many years ago did you receive the last tetanus injection before this pregnancy? *Question relevant when: ${previoustet_alive} =1*	
pregworm_alive *(required)*	During this pregnancy, did you take any drug for intestinal worms?	1 Yes<br>2 No
pregprep_alive *(required)*	During this pregnancy, did you make any preparations for delivery?	1 Yes<br>2 No
Woman of the Household (1) > SECTION 2: REPRODUCTION > PREGNANCY HISTORY (1) > Born alive and still living > ANC for [p5_name_alive] > preparation_alive *Group relevant when: ${pregprep_alive} =1*		
generated_table_list_label_345	What kind of preparation did you make? *Include all responses which the mother mentions unprompted. Then ask, "Is there anything else." Then, read each question and select "yes" or "no."*	
reserved_name_for_field_list_labels_346		1 Yes<br>2 No
preptransport_alive *(required)*	Transport *Question relevant when: ${pregprep_alive} =1*	1 Yes<br>2 No
prepmoney_alive *(required)*	Money *Question relevant when: ${pregprep_alive} =1*	1 Yes<br>2 No
prepfood_alive *(required)*	Food *Question relevant when: ${pregprep_alive} =1*	1 Yes<br>2 No

Field	Question	Answer
prepinstruments_alive *(required)*	Clean instruments for delivery<br>*Question relevant when: ${pregprep_alive} =1*	1 Yes<br>2 No
prepcloths_alive *(required)*	Clean cloths<br>*Question relevant when: ${pregprep_alive} =1*	1 Yes<br>2 No
prepother_alive *(required)*	Other<br>*Question relevant when: ${pregprep_alive} =1*	1 Yes<br>2 No
prep_aliveother	Please specify other preparations<br>*Question relevant when: ${pregprep_alive} =1 and ${prepother_alive} =1*	
delplan_alive *(required)*	Did you discuss planning for your delivery with anybody while you were pregnant?	1 Yes<br>2 No
delplanwhom_alive	Whom did you plan your delivery with?<br>*Question relevant when: ${delplan_alive} =1*	1 Husband<br>2 Mother-in-law<br>3 Father-in-law<br>4 Friends/relative<br>5 CHN/CHO<br>6 Community Health volunteer<br>7 Other health worker<br>8 NA<br>9 DK<br>other Other
delplanwhom_alive_other	Specify other.<br>*Question relevant when: selected(${delplanwhom_alive}, 'other')*	
facdel_alive	Did any health worker give you specific instructions to go deliver at a health facility?	1 NONE<br>2 Doctor<br>3 Midwife<br>4 Nurse<br>5 Community health officer<br>6 Health extension worker<br>other Other
facdel_alive_other	Specify other.<br>*Question relevant when: selected(${facdel_alive}, 'other')*	
whenrec_alive	When did they make this recommendation?<br>*Question relevant when: ${facdel_alive} >3*	1 1st trimester<br>2 2nd trimester<br>3 3rd trimester, but before labor started<br>4 3rd trimester, during labor<br>8 NA<br>9 Don't know<br>other Other
whenrec_alive_other	Specify other.<br>*Question relevant when: selected(${whenrec_alive}, 'other')*	
whyrec_alive	Why did they make this recommendation?<br>*Question relevant when: ${facdel_alive} >3*	1 Does not know why<br>2 Suspected twins<br>3 Position of the baby<br>4 Hypertension/edema /blurred vision<br>5 Previous c-section<br>6 First birth<br>7 Bleeding<br>8 Many hours in labor<br>9 Lack of movement of fetus<br>10 Diabetes<br>11 Anemia<br>88 NA<br>other Other
whyrec_alive_other	Specify other.<br>*Question relevant when: selected(${whyrec_alive}, 'other')*	
otherrec_alive	Did anyone else recommend that you go to deliver at health facility?	1 Husband<br>2 Mother-in-law

Field	Question	Answer
		3 Mother<br>4 Friends/relative<br>5 No one else<br>other Other
otherrec_alive_other	Specify other.<br>*Question relevant when: selected(${otherrec_alive}, 'other')*	
delinstruct_alive	Did any health worker give you specific instructions to call him/her at the time of delivery?	1 NONE<br>2 Doctor<br>3 Midwife<br>4 Nurse<br>5 Community health officer<br>6 Health extension worker<br>other Other
delinstruct_alive_other	Specify other.<br>*Question relevant when: selected(${delinstruct_alive}, 'other')*	

Woman of the Household (1) > SECTION 2: REPRODUCTION > PREGNANCY HISTORY (1) > Born alive and still living > Delivering [p5_name_alive]

Group relevant when: ${U5_alive} <60

Field	Question	Answer
del_alive	Now I would like to ask you some questions about the delivery of your pregnancy with [p5_name_alive] and the care you may have received following his/her birth.	
delplace_alive *(required)*	Where did you give birth to [p5_name_alive]?<br>*Probe to identify the type of source and circle the appropriate code. If unable to determine if a hospital, health center…, write the name of the place in 'other' field*	1 Your home<br>2 Other home<br>3 Govt. Hospital<br>4 Govt. Health center<br>5 CHPS compound<br>6 Govt. Health post<br>7 NGO Facility<br>8 Private facility<br>9 ON THE WAY TO FACILITY<br>other Other
delplace_alive_other	Specify other.<br>*Question relevant when: selected(${delplace_alive}, 'other')*	

Woman of the Household (1) > SECTION 2: REPRODUCTION > PREGNANCY HISTORY (1) > Born alive and still living > Delivering [p5_name_alive] > facreasons_alive

Group relevant when: ${delplace_alive} =1 or ${delplace_alive} =2

Field	Question	Answer
generated_table_list_label_365	What was the reason you didn't deliver in a health facility?<br>*Do Not Read Out Responses PROBE: Any other reason? (Record all mentioned, and rank 1-9 according to importance).*	
expense_alive	Cost too much<br>*Question relevant when: ${delplace_alive} =1 or ${delplace_alive} =2*	
closed_alive	Facility not open<br>*Question relevant when: ${delplace_alive} =1 or ${delplace_alive} =2*	
transport_alive	Too far / no transportation<br>*Question relevant when: ${delplace_alive} =1 or ${delplace_alive} =2*	
trust_alive	Don't trust facility / poor quality service<br>*Question relevant when: ${delplace_alive} =1 or ${delplace_alive} =2*	
maleprov_alive	No female provider at facility<br>*Question relevant when: ${delplace_alive} =1 or ${delplace_alive} =2*	
autonomy_alive	Husband / family did not allow<br>*Question relevant when: ${delplace_alive} =1 or ${delplace_alive} =2*	
unnecessary_alive	Not necessary<br>*Question relevant when: ${delplace_alive} =1 or ${delplace_alive} =2*	
notcustomary_alive	Not customary<br>*Question relevant when: ${delplace_alive} =1 or ${delplace_alive} =2*	
other_fac_alive	Other<br>*Question relevant when: ${delplace_alive} =1 or ${delplace_alive} =2*	

Field	Question	Answer
state_otheralive	Please specify other reason<br>*Question relevant when: ${other_fac_alive} >0*	
Woman of the Household (1) > SECTION 2: REPRODUCTION > PREGNANCY HISTORY (1) > Born alive and still living > Delivering [p5_name_alive] > homereasons_alive<br>*Group relevant when: ${delplace_alive} >2 and ${delplace_alive} <10*		
generated_table_list_label_377	What was the reason you didn't deliver at home?<br>*DO NOT READ OUT RESPONSES Record responses as they are mentioned. The 1st reason mentioned = 1, the 2nd reason mentioned =2, etc.*	
complicate_alive	Experienced complications<br>*Question relevant when: ${delplace_alive} >2 and ${delplace_alive} <10*	
safety_alive	Facilities are safer<br>*Question relevant when: ${delplace_alive} >2 and ${delplace_alive} <10*	
free_alive	Free delivery at the facility<br>*Question relevant when: ${delplace_alive} >2 and ${delplace_alive} <10*	
access_alive	Access to the facility<br>*Question relevant when: ${delplace_alive} >2 and ${delplace_alive} <10*	
quality_alive	Better care at facilities<br>*Question relevant when: ${delplace_alive} >2 and ${delplace_alive} <10*	
other_home_alive	Other<br>*Question relevant when: ${delplace_alive} >2 and ${delplace_alive} <10*	
specify_otheralive	Please specify other reason<br>*Question relevant when: ${other_home_alive} >0*	
delcompwho_alive *(required)*	Who accompanied you to the health facility?<br>*Question relevant when: ${delplace_alive} >2 and ${delplace_alive} <10*	1 Nobody<br>2 Mother-in-law<br>3 Father-in-law<br>4 Husband<br>5 Other relative<br>6 CHO/CHN<br>9 DK<br>other Other
delcompwho_alive_other	Specify other.<br>*Question relevant when: selected(${delcompwho_alive}, 'other')*	
del_transportalive *(required)*	How did you travel to the health facility?<br>*Question relevant when: ${delplace_alive} >2 and ${delplace_alive} <9*	1 by foot (walking)<br>2 personal bicycle/motorbike /motoking<br>3 public transport (bus/taxi)<br>4 emergency motorking<br>5 ambulance<br>6 other
otherdel_transportalive *(required)*	Please specify transport used<br>*Question relevant when: ${del_transportalive} =6*	
homeassistwho_alive *(required)*	Who assisted with the delivery of [p5_name_alive]? Anyone else?<br>*Probe for the type(s) of person(s) and record all mentioned. If respondent says 'no one assisted', probe to determine whether any adults were present at the delivery.*	1 Doctor<br>2 Midwife<br>3 Nurse<br>4 Community health officer<br>5 Health extension worker<br>6 Trained TBA<br>7 Community health volunteer<br>8 Untrained TBA<br>9 Relative/friends<br>10 Nobody<br>11 Other
otherassist_alive	Please specify<br>*Question relevant when: ${homeassistwho_alive} =11*	
delcomp_alive *(required)*	During your most recent delivery, did you, personally, experience any of the following symptoms?	1 Convulsions<br>2 Long labor<br>3 Baby hand or feet coming first/abnormal position

Field	Question	Answer
		4 Excessive bleeding/shock<br>5 None<br>6 Others
othercomp_alive	Please specify other complications<br>*Question relevant when: ${delcomp_alive} =6*	
symptoms_alive *(required)*	When you experienced these symptoms, were you told to go to a health facility?<br>*Question relevant when: ${delcomp_alive} !=5*	1 Yes<br>2 Experienced symptoms while at a health facility<br>3 No<br>8 NA<br>9 DK
healthfacwho_alive	By whom?<br>*Question relevant when: ${delcomp_alive} !=5 and ${symptoms_alive} =1*	1 Doctor<br>2 Midwife<br>3 Nurse<br>4 Community health officer<br>5 Health extension worker<br>6 Trained TBA<br>7 Community health volunteer<br>8 Untrained TBA<br>9 Relative/friends<br>10 Nobody<br>11 Other
delassito_alive	Please specify<br>*Question relevant when: ${healthfacwho_alive} =11*	
Csec_alive *(required)*	Was [p5_name_alive] delivered by caesarean section, i.e., did they cut your belly open to take the baby out?<br>*Question relevant when: ${delplace_alive} >2 and ${delplace_alive} <9*	1 Yes<br>2 No
dried_alive *(required)*	Was the baby wiped (dried) before the placenta was delivered?	1 Yes<br>2 No<br>3 Don't Know
babyplaced_alive *(required)*	Where was the baby placed immediately after delivery?	1 On the floor<br>2 On a cot<br>3 On the mother's abdomen<br>4 With someone else<br>8 NA<br>9 DK<br>other Other
babyplaced_alive_other	Specify other.<br>*Question relevant when: selected(${babyplaced_alive}, 'other')*	
swaddle_alive *(required)*	Was the baby wrapped with cloth before the placenta was delivered?	1 Yes<br>2 No<br>9 Don't know<br>8 Not applicable

Field	Question	Answer
cry_alive *(required)*	Did your baby cry or breathe easily immediately after birth?	1 Yes<br>2 No<br>9 Don't know<br>8 Not applicable
helpbreathe_alive *(required)*	What was done to help the baby cry or breathe at the time of birth?<br>*Please do not read out responses. ASK: Anything else? Record all responses*<br>*Question relevant when: ${cry_alive} !=1*	1 Rubbed/massaged<br>2 Dried<br>3 Mouth cleared<br>4 Nothing<br>8 NA<br>9 DK<br>other Other
helpbreathe_alive_other	Specify other.<br>*Question relevant when: selected(${helpbreathe_alive}, 'other')*	
breatheassist_alive	Who took these measures to help the baby cry or breathe?<br>*Please do not read out responses.*<br>*Question relevant when: ${cry_alive} !=1 and ${helpbreathe_alive} !=4*	1 Doctor<br>2 Midwife<br>3 Nurse<br>4 Community health officer<br>5 Health extension worker<br>6 Trained TBA<br>7 Community health volunteer<br>8 Untrained TBA<br>9 Relative/friends<br>10 Nobody<br>11 Other
size_alive *(required)*	When [p5_name_alive] was born, was he/she very large, large, average, and smaller than average or very small?	1 Very large<br>2 Larger than average<br>3 Average<br>4 Smaller than average<br>5 Very small<br>9 Don't know
keepwarm_alive *(required)*	What was done to keep [p5_name_alive] warm after delivery (within the first day after birth)? Anything else?<br>*Multiple responses possible*	1 Dried the baby<br>2 Wrapped the baby with clean cloths<br>3 Put baby beside the mother<br>4 Kept the baby on bare skin to skin contact<br>5 Bathed in warm water<br>6 Warmed delivery room<br>7 Nothing done<br>8 NA<br>9 DK<br>other Other
keepwarm_alive_other	Specify other.<br>*Question relevant when: selected(${keepwarm_alive}, 'other')*	

Field	Question	Answer
bftime_alive *(required)*	How long after birth did you first put [p5_name_alive] to the breast?<br>*If less than 1 hour, record '00' hours. If less than 24 hours, record hours. Otherwise, record days.*	1 Hours<br>2 Days<br>8 Never breastfed<br>9 DK
bfhours_alive	Hours<br>*Question relevant when: ${bftime_alive} =1*<br>*Response constrained to: .<24*	
bfdays_alive	Days<br>*Question relevant when: ${bftime_alive} =2*	
otherfood_alive *(required)*	When did you first start giving food or drink other than breast milk to [p5_name_alive]?<br>*Question relevant when: ${bftime_alive} !=8*	1 Hours<br>2 Days<br>3 Months<br>4 Baby exclusively breastfed until now<br>8 NA<br>9 DK
otherfoodhours_alive	Hours<br>*Question relevant when: ${otherfood_alive} =1*<br>*Response constrained to: .<24*	
otherfooddays_alive	Days<br>*Question relevant when: ${otherfood_alive} =2*<br>*Response constrained to: .<32*	
otherfoodmonths_alive	Months<br>*Question relevant when: ${otherfood_alive} =3*	
stillbreastfed_alive *(required)*	Are you still breastfeeding [p5_name_alive]?<br>*Question relevant when: ${bftime_alive} !=8 and ${otherfood_alive} !=4*	1 Yes<br>2 No<br>3 NA
bflength_alive *(required)*	How long, in all, did you breastfeed [p5_name_alive]?<br>*Question relevant when: ${bftime_alive} !=8 and ${stillbreastfed_alive} !=1 and ${otherfood_alive} !=4*	1 Months<br>2 Until child died<br>8 NA<br>9 DK
bfmonths_alive	Months<br>*Question relevant when: ${bflength_alive} =1 and ${stillbreastfed_alive} !=1*	
stopbf_alive	Why did you stop breastfeeding [p5_name_alive]?<br>*Question relevant when: ${bftime_alive} !=8 and ${stillbreastfed_alive} !=1 and ${otherfood_alive} !=4*	1 Mother ill/weak<br>2 Child ill/weak

Field	Question	Answer
		3 Child died<br>4 Nipple/breast problem<br>5 Not enough milk<br>6 Mother working<br>7 Child refused<br>8 Weaning age<br>9 Became pregnant<br>88 NA<br>other Other
stopbf_alive_other	Specify other.<br>*Question relevant when: selected(${stopbf_alive}, 'other')*	
facstay_alive *(required)*	How long did you stay at the facility following your delivery?<br>*Question relevant when: ${delplace_alive} !=1 and ${delplace_alive} !=2*	1 Hours<br>2 Days<br>3 Months<br>8 NA<br>9 DK
fachr_alive	Hours<br>*Question relevant when: ${facstay_alive} =1*<br>*Response constrained to: .<24*	
facday_alive	Days<br>*Question relevant when: ${facstay_alive} =2*<br>*Response constrained to: .<32*	
facmo_alive	Months<br>*Question relevant when: ${facstay_alive} =3*	

Woman of the Household (1) > SECTION 2: REPRODUCTION > PREGNANCY HISTORY (1) > Born alive and still living > PNC for [p5_name_alive]

Group relevant when: ${U5_alive} <60 and ${momPNCwkvisits_alive} >1 or ${babyPNCtotvisits_alive} >1

Field	Question	Answer
PNCfac_alive *(required)*	Before you were discharged after [p5_name_alive] was born, did any health care provider check on you or your newborn's health?<br>*Question relevant when: ${delplace_alive} !=1 and ${delplace_alive} !=2*	1 Yes<br>2 No<br>3 Don't Know
PNChome_alive	Before the Health Professional, CHO/CHN or traditional birth attendant left your house, after [p5_name_alive] was born, did he/she check on your health?<br>*Question relevant when: ${delplace_alive} =1 or ${delplace_alive} =2*	1 Yes<br>2 No

Field	Question	Answer
PNCcheck_alive	Not including this first check, after [p5_name_alive] was born, did any health care provider check on you or your baby's health in the first week?<br>*PROBE: Any visits where the health care provider or CHW came to your home after delivery to talk, provide counseling and examine yourself or your baby?*	1 Yes<br>2 No
momPNCtotvisits_alive	How many visits did YOU have in total following the delivery?<br>*PROBE: Any counseling given to the Mother for care of her baby is considered a visit for the baby*<br>*Question relevant when: ${PNCcheck_alive} =1 and ${PNCfac_alive} =1 or ${PNCfac_alive} =3 or ${PNChome_alive} =1 or ${PNChome_alive} =3*	
babyPNCtotvisits_alive *(required)*	How many visits did your BABY have in total following the delivery?<br>*PROBE: Any counseling given to the Mother for care of her baby is considered a visit for the baby*<br>*Question relevant when: ${PNCcheck_alive} =1 and ${PNCfac_alive} =2 or ${PNCfac_alive} =3 or ${PNChome_alive} =2 or ${PNChome_alive} =3*	
momPNCwkvisits_alive *(required)*	How many visits did YOU have in the first week following the delivery?<br>*PROBE: Any counseling given to the Mother for care of her baby is considered a visit for the baby*<br>*Question relevant when: ${PNCcheck_alive} =1 and ${momPNCtotvisits_alive} >0*<br>*Response constrained to: .< ${momPNCtotvisits_alive}*	
babyPNCwkvisits_alive *(required)*	How many visits did [p5_name_alive] have in the first week following the delivery?<br>*PROBE: Any counseling given to the Mother for care of her baby is considered a visit for the baby*<br>*Question relevant when: ${PNCcheck_alive} =1 and ${babyPNCtotvisits_alive} >0*<br>*Response constrained to: .< ${babyPNCtotvisits_alive}*	
momPNCtime_alive *(required)*	After [p5_name_alive] was born, how many hours, days or weeks after the birth did somebody first check on YOUR health?<br>*Question relevant when: ${PNCcheck_alive} =1 and ${momPNCwkvisits_alive} >0*	1 Hours<br>2 Days<br>3 Weeks<br>8 NA<br>9 DK
momPNChours_alive	Hours<br>*Question relevant when: ${momPNCtime_alive} =1*<br>*Response constrained to: .<24*	
momPNCdays_alive	Days<br>*Question relevant when: ${momPNCtime_alive} =2*<br>*Response constrained to: .<32*	
momPNCweeks_alive	Weeks<br>*Question relevant when: ${momPNCtime_alive} =3*	
babyPNCtime_alive *(required)*	After [p5_name_alive] was born, how many hours, days or weeks after the birth did somebody first check on your BABY's health?<br>*Question relevant when: ${PNCcheck_alive} =1 and ${babyPNCwkvisits_alive} >1*	1 Hours<br>2 Days<br>3 Weeks<br>8 NA<br>9 DK
babyPNChours_alive	Hours<br>*Question relevant when: ${babyPNCtime_alive} =1*<br>*Response constrained to: .<24*	
babyPNCdays_alive	Days<br>*Question relevant when: ${babyPNCtime_alive} =2*<br>*Response constrained to: .<32*	

Field	Question	Answer
babyPNCweeks_alive	Weeks<br>*Question relevant when: ${babyPNCtime_alive} =3*	
momPNCprov_alive *(required)*	Who checked on YOUR health at that time? Anyone else?<br>*Question relevant when: ${PNCcheck_alive} =1 and ${momPNCwkvisits_alive} >0*	1 Doctor<br>2 Midwife<br>3 Nurse<br>4 Community health officer<br>5 Health extension worker<br>6 Trained TBA<br>7 Community health volunteer<br>8 Untrained TBA<br>9 Relative/friends<br>10 Nobody<br>11 Other<br>other Other
momPNCprov_alive_other	Specify other.<br>*Question relevant when: selected(${momPNCprov_alive}, 'other')*	
babyPNCprov_alive *(required)*	Who checked on your [p5_name_alive]'s health at that time? Anyone else?<br>*Question relevant when: ${PNCcheck_alive} =1 and ${babyPNCwkvisits_alive} >0*	1 Doctor<br>2 Midwife<br>3 Nurse<br>4 Community health officer<br>5 Health extension worker<br>6 Trained TBA<br>7 Community health volunteer<br>8 Untrained TBA<br>9 Relative/friends<br>10 Nobody<br>11 Other<br>other Other
babyPNCprov_alive_other	Specify other.<br>*Question relevant when: selected(${babyPNCprov_alive}, 'other')*	
momPNCplace_alive	Who checked on YOUR health at that time? Anyone else?<br>*Probe to identify the type of source and circle the appropriate code. If unable to determine if a hospital, health center, or clinic is public or private medical, write the name of the place in 'other' field.*<br>*Question relevant when: ${PNCcheck_alive} =1 and ${momPNCwkvisits_alive} >1*	1 Your home<br>2 Other home<br>3 Govt. Hospital<br>4 Govt. Health center<br>5 CHPS compound<br>6 Govt. Health post<br>7 NGO Facility<br>8 Private facility<br>9 ON THE WAY TO FACILITY<br>other Other
momPNCplace_alive_other	Specify other.<br>*Question relevant when: selected(${momPNCplace_alive}, 'other')*	
babyPNCplace_alive *(required)*	Who checked on [p5_name_alive]'s health at that time? Anyone else?<br>*Probe to identify the type of source and circle the appropriate code. If unable to determine if a hospital, health center, or clinic is public or private medical, write the name of the place in 'other' field.*<br>*Question relevant when: ${PNCcheck_alive} =1 and ${babyPNCwkvisits_alive} >1*	1 Your home<br>2 Other home<br>3 Govt. Hospital<br>4 Govt. Health center<br>5 CHPS compound<br>6 Govt. Health post<br>7 NGO Facility<br>8 Private facility

Field	Question	Answer
		9 ON THE WAY TO FACILITY<br>other Other
babyPNCplace_alive_other	Specify other.<br>*Question relevant when: selected(${babyPNCplace_alive}, 'other')*	
healthcheck_alive	What did the health worker/other do during that visit to check on YOUR health?<br>*Question relevant when: ${PNCcheck_alive} =1 and ${momPNCwkvisits_alive} >1*	1 Examined body<br>2 Checked breasts<br>3 Checked for heavy bleeding<br>4 Counseled on danger signs for newborns<br>5 Counseled on breastfeeding<br>6 Counseled on nutrition<br>8 NA<br>other Other
healthcheck_alive_other	Specify other.<br>*Question relevant when: selected(${healthcheck_alive}, 'other')*	

Woman of the Household (1) > SECTION 2: REPRODUCTION > PREGNANCY HISTORY (1) > Born alive and died later

Group relevant when: ${p3} =1 and ${p6a} =2

Field	Question	Answer
multiple_died *(required)*	Was this a multiple birth?<br>*For multiple births, add the other birth separately as a new group with same month and year of birth and this birth*	1 Yes<br>2 No
p2a_died *(required)*	How many months did this pregnancy last?<br>*Response constrained to: .<13*	
p5_died *(required)*	Was a name given to that baby?	1 Yes<br>2 No<br>9 Don't know<br>8 Not applicable
p5_name_died *(required)*	Name<br>*Question relevant when: ${p5_died} =1*	
p6_died *(required)*	Was [p5_name_died] a boy or a girl?	1 Male<br>2 Female

Woman of the Household (1) > SECTION 2: REPRODUCTION > PREGNANCY HISTORY (1) > Born alive and died later > In what month and year did you give birth to [p5_name_died]?

Field	Question	Answer
p2b_mo_died *(required)*	In what month and year did you give birth to [p5_name_died]?<br>*Response constrained to: .<13 or .=99*	1 January<br>2 February<br>3 March<br>4 April<br>5 May<br>6 June<br>7 July<br>8 August<br>9 September<br>10 October

Field	Question	Answer
		11 November<br>12 December
p2b_yr_died *(required)*	Year<br>*YYYY*<br>*Response constrained to: .>1975 and .<2015*	
p9_yr *(required)*	How old was [p5_name_died] when he/she died?<br>*If >1 year old at death, enter age in years. Otherwise, enter 0 here and months in the next question*	
p9_mo	Months<br>*Enter age in months at time of death*<br>*Question relevant when: ${p9_yr} =0*<br>*Response constrained to: .<12*	
p9_day	Days<br>*Question relevant when: ${p9_mo} =0*<br>*Response constrained to: .<=31*	
Woman of the Household (1) > SECTION 2: REPRODUCTION > PREGNANCY HISTORY (1) > Born alive and died later > In what month and year did [p5_name_died] die?		
p10_mo *(required)*	In what month and year did [p5_name_died] die?	1 January<br>2 February<br>3 March<br>4 April<br>5 May<br>6 June<br>7 July<br>8 August<br>9 September<br>10 October<br>11 November<br>12 December
p10_yr *(required)*	Year<br>*YYYY*<br>*Response constrained to: .>1975 and .<2015*	
Woman of the Household (1) > SECTION 2: REPRODUCTION > PREGNANCY HISTORY (1) > Born alive and died later > ANC for [p5_name_died]<br>*Group relevant when: ${U5_died} <60*		
pregintent_died *(required)*	When you got pregnant with [p5_name_died], did you want to get pregnant at that time?	1 Yes<br>2 No<br>3 Don't Know
pregtiming_died *(required)*	Did you want to have a baby later on or did you not want any more children?<br>*Question relevant when: ${pregintent_died} !=1*	1 Later<br>2 No more<br>8 NA
wantedwait_died *(required)*	How much longer did you want to wait?<br>*Question relevant when: ${pregintent_died} !=1 and ${pregtiming_died} !=2*	1 Months<br>2 Years<br>8 NA
wantedwait_mo_died	Months<br>*Question relevant when: ${wantedwait_died} =1*	
wantedwait_yr_died	Years<br>*Question relevant when: ${wantedwait_died} =2*	

Field	Question	Answer
ANCcheck_died *(required)*	Did you see anyone for antenatal care during this pregnancy?	1 Yes<br>2 No
ANCprov_died *(required)*	Whom did you see?<br>*Question relevant when: ${ANCcheck_died} =1*	1 Doctor<br>2 Midwife<br>3 Nurse<br>4 Community health officer<br>5 Health extension worker<br>6 Traditional birth attendant<br>7 Other health worker<br>other Other
ANCprov_died_other	Specify other.<br>*Question relevant when: selected(${ANCprov_died}, 'other')*	
ANCplace_died *(required)*	Where did you receive antenatal care for this pregnancy? Anywhere else?<br>*Probe to identify type(s) of source(s) and circle the appropriate code(s). If there is no appropriate code, select 'other', then enter the name of the place.*<br>*Question relevant when: ${ANCcheck_died} =1*	1 Govt. Hospital<br>2 Govt. Health center/Clinic<br>3 CHPS compound<br>4 NGO facility<br>5 Private Clinic<br>6 Home<br>other Other
ANCplace_died_other	Specify other.<br>*Question relevant when: selected(${ANCplace_died}, 'other')*	
pinkbook_died *(required)*	Have you ever had a maternal health book (pink book) for this pregnancy?<br>*Question relevant when: ${ANCcheck_died} =1*	1 Yes, seen<br>2 Yes, not seen<br>3 No<br>8 NA
pinkbookANCvisits_died	Enter number of visits from health book<br>*Question relevant when: ${pinkbook_died} =1*	

Field	Question	Answer
ANC_mo_died	How many months pregnant were you when you first received antenatal care for this pregnancy?<br>*88=NA, 99=DK*<br>*Question relevant when: ${ANCcheck_died} =1*	
firstprov_died *(required)*	Who did you first receive antenatal care from during your pregnancy?<br>*Question relevant when: ${ANCcheck_died} =1*	1 Doctor<br>2 Midwife<br>3 Nurse<br>4 Community health officer<br>5 Health extension worker<br>6 Traditional birth attendant<br>7 Other health worker
firstplace_died *(required)*	Where did you first receive antenatal care from during your pregnancy?<br>*Question relevant when: ${ANCcheck_died} =1*	1 Govt. Hospital<br>2 Govt. Health center/Clinic<br>3 CHPS compound<br>4 NGO facility<br>5 Private Clinic<br>6 Home<br>other Other
firstplace_died_other	Specify other.<br>*Question relevant when: selected(${firstplace_died}, 'other')*	
srANCvisits_died *(required)*	How many times in total did you receive antenatal care during this pregnancy?<br>*88=NA, 99=DK*<br>*Question relevant when: ${ANCcheck_died} =1*	
mismatch_died	I notice that you mentioned receiving a number of ANC visits that is different than are listed in your book. Sometimes women may forget to bring their book to ANC; sometime the health worker may also not write in your book? Did this happen to you?<br>*Multiple answers allowed*<br>*Question relevant when: ${pinkbookANCvisits_died} != ${srANCvisits_died}*	1 Forgot to bring book<br>2 HW did not write<br>3 Other reason<br>other Other
mismatch_died_other	Specify other.<br>*Question relevant when: selected(${mismatch_died}, 'other')*	

Woman of the Household (1) > SECTION 2: REPRODUCTION > PREGNANCY HISTORY (1) > Born alive and died later > ANC for [p5_name_died] > duringpreg_died

Group relevant when: ${ANCcheck_died} =1

Field	Question	Answer
generated_table_list_label_489	During this pregnancy:	
reserved_name_for_field_list_labels_490		1 Yes<br>2 No
pregweight_died *(required)*	Were you weighed?<br>*Question relevant when: ${ANCcheck_died} =1*	1 Yes<br>2 No
pregBP_died *(required)*	Was your blood pressure measured?<br>*Question relevant when: ${ANCcheck_died} =1*	1 Yes<br>2 No

Field	Question	Answer
pregheight_died *(required)*	Was your height measured?<br>*Question relevant when: ${ANCcheck_died} =1*	1 Yes<br>2 No
preguring_died *(required)*	Did you give a urine sample?<br>*Question relevant when: ${ANCcheck_died} =1*	1 Yes<br>2 No
pregblood_died *(required)*	Did you give a blood sample?<br>*Question relevant when: ${ANCcheck_died} =1*	1 Yes<br>2 No
pregstomach_died *(required)*	Was your stomach measured?<br>*Question relevant when: ${ANCcheck_died} =1*	1 Yes<br>2 No
pregHR_died *(required)*	Did a health worker listen to the heart rate of your baby?<br>*Question relevant when: ${ANCcheck_died} =1*	1 Yes<br>2 No

Woman of the Household (1) > SECTION 2: REPRODUCTION > PREGNANCY HISTORY (1) > Born alive and died later > ANC for [p5_name_died] > duringpregHW_died

Group relevant when: ${ANCcheck_died} =1

Field	Question	Answer
generated_table_list_label_498	During this pregnancy, were you counseled by a health worker on:	
reserved_name_for_field_list_labels_499		1 Yes<br>2 No
pregfinprep_died *(required)*	Financial preparation for your delivery?<br>*Question relevant when: ${ANCcheck_died} =1*	1 Yes<br>2 No
pregbf_died *(required)*	Breastfeeding immediately after delivery?<br>*Question relevant when: ${ANCcheck_died} =1*	1 Yes<br>2 No
pregtt_died *(required)*	Tetanus toxic vaccination?<br>*Question relevant when: ${ANCcheck_died} =1*	1 Yes<br>2 No
pregdanger_died *(required)*	Danger signs during delivery?<br>*Question relevant when: ${ANCcheck_died} =1*	1 Yes<br>2 No
pregwrap_died *(required)*	Wrapping the newborn after delivery?<br>*Question relevant when: ${ANCcheck_died} =1*	1 Yes<br>2 No
pregSBA_died *(required)*	Using a skilled birth attendant?<br>*Question relevant when: ${ANCcheck_died} =1*	1 Yes<br>2 No
pregFP_died *(required)*	Family planning?<br>*Question relevant when: ${ANCcheck_died} =1*	1 Yes<br>2 No

Field	Question	Answer
pregtransport_died *(required)*	Identifying emergency transport options?<br>*Question relevant when: ${ANCcheck_died} =1*	1 Yes<br>2 No
pregdiet_died *(required)*	Diet?<br>*Question relevant when: ${ANCcheck_died} =1*	1 Yes<br>2 No
pregother_died *(required)*	Other<br>*Question relevant when: ${ANCcheck_died} =1*	1 Yes<br>2 No
other_died	Please specify other things you were conseled on<br>*Question relevant when: ${pregother_died} =1*	
dangersigns_died *(required)*	During (any of) your antenatal care visit(s) with [p5_name_died], were you told about the things to look out for that might suggest problems with the pregnancy?<br>*Question relevant when: ${ANCcheck_died} =1*	1 Yes<br>2 No
dangerplace_died *(required)*	Were you told where to go if you had any of these complications?<br>*Question relevant when: ${ANCcheck_died} =1 and ${dangersigns_died} =1*	1 Yes<br>2 No
pregbednet_died *(required)*	At the time of this pregnancy, did your household have any bed net?	1 Yes<br>2 No<br>3 Don't Know
sleepfreq_died *(required)*	How often did you sleep under a bed net during this pregnancy?<br>*Question relevant when: ${pregbednet_died} !=2*	1 Every night<br>2 Most nights<br>3 Some nights<br>4 Rarely<br>5 Never<br>8 NA
pregmalaria_died *(required)*	During this pregnancy, did you take any drugs to keep you from getting malaria?	1 Yes<br>2 No
pregmalariameds_died	What drugs did you take?<br>*Question relevant when: ${pregmalaria_died} =1*	1 SP<br>2 ACT<br>8 NA<br>9 DK<br>other Other
pregmalariameds_died_other	Specify other.<br>*Question relevant when: selected(${pregmalariameds_died}, 'other')*	
SPtimes_died	How many times did you take SP?<br>*Enter 99 for DK*<br>*Question relevant when: ${pregmalariameds_died} =1*	
ACTtimes_died	How many times did you take ACT?<br>*Enter 99 for DK*<br>*Question relevant when: ${pregmalariameds_died} =2*	
drugs_died	Did you get the drugs during any antenatal care visit, during another visit to a health facility or from another source?<br>*Question relevant when: ${pregmalariameds_died} =1*	1 ANC Visit<br>2 Other health facility visit<br>3 Other source<br>8 NA
tetanus_died *(required)*	During this pregnancy were you given an injection in the arm to prevent you and the baby from getting tetanus?	1 Yes<br>2 No
tetanustimes_died *(required)*	During this pregnancy, how many times did you get this tetanus injection?<br>*Question relevant when: ${tetanus_died} =1*	
previoustet_died *(required)*	At any time before this pregnancy, did you receive any tetanus injections?	1 Yes<br>2 No<br>9 Don't know<br>8 Not applicable
prevtettimes_died	Before this pregnancy, how many tetanus injections did you receive in total?<br>*Question relevant when: ${previoustet_died} =1*	
prevtetyr_died	How many years ago did you receive the last tetanus injection before this pregnancy?<br>*Question relevant when: ${previoustet_died} =1*	
pregworm_died *(required)*	During this pregnancy, did you take any drug for intestinal worms?	1 Yes<br>2 No
pregprep_died	During this pregnancy, did you make any preparations for delivery?	1 Yes<br>2 No
Woman of the Household (1) > SECTION 2: REPRODUCTION > PREGNANCY HISTORY (1) > Born alive and died later > ANC for [p5_name_died] > preparation_died<br>*Group relevant when: ${pregprep_died} =1*		
generated_table_list_label_527	What kind of preparation did you make?<br>*Include all responses which the mother mentions unprompted. Then ask, "Is there anything else." Then, read each question and select "yes" or "no."*	

Field	Question	Answer
reserved_name_for_field_list_labels_528		1 Yes<br>2 No
preptransport_died	Transport<br>*Question relevant when: ${pregprep_died} =1*	1 Yes<br>2 No
prepmoney_died	Money<br>*Question relevant when: ${pregprep_died} =1*	1 Yes<br>2 No
prepfood_died	Food<br>*Question relevant when: ${pregprep_died} =1*	1 Yes<br>2 No
prepinstruments_died	Clean instruments for delivery<br>*Question relevant when: ${pregprep_died} =1*	1 Yes<br>2 No
prepcloths_died	Clean cloths<br>*Question relevant when: ${pregprep_died} =1*	1 Yes<br>2 No
prepother_died	Other<br>*Question relevant when: ${pregprep_died} =1*	1 Yes<br>2 No
prep_diedother	Please specify other preparations<br>*Question relevant when: ${pregprep_died} =1 and ${prepother_died} =1*	
delplan_died	Did you discuss planning for your delivery with anybody while you were pregnant?	1 Yes<br>2 No
delplanwhom_died	Whom did you plan your delivery with?<br>*Question relevant when: ${delplan_died} =1*	1 Husband<br>2 Mother-in-law<br>3 Father-in-law<br>4 Friends/relative<br>5 CHN/CHO<br>6 Community Health volunteer<br>7 Other health worker<br>8 NA<br>9 DK<br>other Other
delplanwhom_died_other	Specify other.<br>*Question relevant when: selected(${delplanwhom_died}, 'other')*	
facdel_died	Did any health worker give you specific instructions to go deliver at a health facility?	1 NONE<br>2 Doctor<br>3 Midwife<br>4 Nurse<br>5 Community health officer<br>6 Health extension worker<br>other Other
facdel_died_other	Specify other.<br>*Question relevant when: selected(${facdel_died}, 'other')*	
whenrec_died	When did they make this recommendation?<br>*Question relevant when: ${facdel_died} >3*	1 1st trimester<br>2 2nd trimester<br>3 3rd trimester, but before labor started<br>4 3rd trimester, during labor<br>8 NA<br>9 Don't know<br>other Other
whenrec_died_other	Specify other.<br>*Question relevant when: selected(${whenrec_died}, 'other')*	
whyrec_died	Why did they make this recommendation?<br>*Question relevant when: ${facdel_died} >3*	1 Does not know why<br>2 Suspected twins<br>3 Position of the baby

Field	Question	Answer
		4 Hypertension/edema /blurred vision<br>5 Previous c-section<br>6 First birth<br>7 Bleeding<br>8 Many hours in labor<br>9 Lack of movement of fetus<br>10 Diabetes<br>11 Anemia<br>88 NA<br>other Other
whyrec_died_other	Specify other.<br>*Question relevant when: selected(${whyrec_died}, 'other')*	
otherrec_died	Did anyone else recommend that you go to deliver at health facility?	1 Husband<br>2 Mother-in-law<br>3 Mother<br>4 Friends/relative<br>5 No one else<br>other Other
otherrec_died_other	Specify other.<br>*Question relevant when: selected(${otherrec_died}, 'other')*	
delinstruct_died	Did any health worker give you specific instructions to call him/her at the time of delivery?	1 NONE<br>2 Doctor<br>3 Midwife<br>4 Nurse<br>5 Community health officer<br>6 Health extension worker<br>other Other
delinstruct_died_other	Specify other.<br>*Question relevant when: selected(${delinstruct_died}, 'other')*	

Woman of the Household (1) > SECTION 2: REPRODUCTION > PREGNANCY HISTORY (1) > Born alive and died later > Delivering [p5_name_died]

Group relevant when: ${U5_died} <60

Field	Question	Answer
del_died	Now I would like to ask you some questions about the delivery of your pregnancy with [p5_name_alive] and the care you may have received following his/her birth. Interviewer (determine name of the baby according to pregnancy table). If woman is currently pregnant, ask about last delivery prior to the current pregnancy.	
delplace_died *(required)*	Where did you give birth to [p5_name_alive]?<br>*Probe to identify the type of source and circle the appropriate code. If unable to determine if a hospital, health center…, write the name of the place in 'other' field*	1 Your home<br>2 Other home<br>3 Govt. Hospital<br>4 Govt. Health center<br>5 CHPS compound<br>6 Govt. Health post<br>7 NGO Facility<br>8 Private facility<br>9 ON THE WAY TO FACILITY<br>other Other
delplace_died_other	Specify other.<br>*Question relevant when: selected(${delplace_died}, 'other')*	

Woman of the Household (1) > SECTION 2: REPRODUCTION > PREGNANCY HISTORY (1) > Born alive and died later > Delivering [p5_name_died] > facreasons_died

Group relevant when: ${delplace_died} =1 or ${delplace_died} =2

Field	Question	Answer
generated_table_list_label_550	What was the reason you didn't deliver in a health facility?<br>*Do Not Read Out Responses PROBE: Any other reason? (Record all mentioned, and rank 1-9 according to importance).*	
expense_died	Cost too much<br>*Question relevant when: ${delplace_died} =1 or ${delplace_died} =2*	
closed_died	Facility not open<br>*Question relevant when: ${delplace_died} =1 or ${delplace_died} =2*	
transport_died	Too far / no transportation<br>*Question relevant when: ${delplace_died} =1 or ${delplace_died} =2*	
trust_died	Don't trust facility / poor quality service<br>*Question relevant when: ${delplace_died} =1 or ${delplace_died} =2*	

Field	Question	Answer
maleprov_died	No female provider at facility<br>*Question relevant when: ${delplace_died} =1 or ${delplace_died} =2*	
autonomy_died	Husband / family did not allow<br>*Question relevant when: ${delplace_died} =1 or ${delplace_died} =2*	
unnecessary_died	Not necessary<br>*Question relevant when: ${delplace_died} =1 or ${delplace_died} =2*	
notcustomary_died	Not customary<br>*Question relevant when: ${delplace_died} =1 or ${delplace_died} =2*	
other_fac_died	Other<br>*Question relevant when: ${delplace_died} =1 or ${delplace_died} =2*	
state_otherdied	Please specify other reason<br>*Question relevant when: ${other_fac_died} >0*	
Woman of the Household (1) > SECTION 2: REPRODUCTION > PREGNANCY HISTORY (1) > Born alive and died later > Delivering [p5_name_died] > homereasons_died<br>*Group relevant when: ${delplace_died} >2 and ${delplace_died} <10*		
generated_table_list_label_562	What was the reason you didn't deliver at home?<br>*Do Not Read Out Responses PROBE: Any other reason? (Record all mentioned, and rank 1-9 according to importance).*	
complicate_died	Experienced complications<br>*Question relevant when: ${delplace_died} >2 and ${delplace_died} <10*	
safety_died	Facilities are safer<br>*Question relevant when: ${delplace_died} >2 and ${delplace_died} <10*	
free_died	Free delivery at the facility<br>*Question relevant when: ${delplace_died} >2 and ${delplace_died} <10*	
access_died	Access to the facility<br>*Question relevant when: ${delplace_died} >2 and ${delplace_died} <10*	
quality_died	Better care at facilities<br>*Question relevant when: ${delplace_died} >2 and ${delplace_died} <10*	
other_home_died	Other<br>*Question relevant when: ${delplace_died} >2 and ${delplace_died} <10*	
specify_otherdied	Please specify other reason<br>*Question relevant when: ${other_home_died} >0*	
delcompwho_died	Who accompanied you to the health facility?<br>*Question relevant when: ${delplace_died} >2 and ${delplace_died} <10*	1 Nobody<br>2 Mother-in-law<br>3 Father-in-law<br>4 Husband<br>5 Other relative<br>6 CHO/CHN<br>9 DK<br>other Other
delcompwho_died_other	Specify other.<br>*Question relevant when: selected(${delcompwho_died}, 'other')*	
del_transportdied *(required)*	How did you travel to the health facility?<br>*Question relevant when: ${delplace_died} >2 and ${delplace_died} <9*	1 by foot (walking)<br>2 personal bicycle/motorbike /motoking

Field	Question	Answer
		3 public transport (bus/taxi)<br>4 emergency motorking<br>5 ambulance<br>6 other
otherdel_transportdied *(required)*	Please specify transport used<br>*Question relevant when: ${del_transportdied} =6*	
homeassistwho_died *(required)*	Who assisted with the delivery of [p5_name_died]? Anyone else?<br>*Probe for the type(s) of person(s) and record all mentioned. If respondent says 'no one assisted', probe to determine whether any adults were present at the delivery.*	1 Doctor<br>2 Midwife<br>3 Nurse<br>4 Community health officer<br>5 Health extension worker<br>6 Trained TBA<br>7 Community health volunteer<br>8 Untrained TBA<br>9 Relative/friends<br>10 Nobody<br>11 Other
otherassist_died	Please specify<br>*Question relevant when: ${homeassistwho_died} =11*	
delcomp_died *(required)*	During your most recent delivery, did you, personally, experience any of the following symptoms?	1 Convulsions<br>2 Long labor<br>3 Baby hand or feet coming first/abnormal position<br>4 Excessive bleeding/shock<br>5 None<br>6 Others
othercomp_died	Please specify other complications<br>*Question relevant when: ${delcomp_died} =6*	
symptoms_died *(required)*	When you experienced these symptoms, were you told to go to a health facility?<br>*Question relevant when: ${delcomp_died} !=5*	1 Yes<br>2 Experienced symptoms while at a health facility<br>3 No<br>8 NA<br>9 DK
healthfacwho_died	By whom?<br>*Question relevant when: ${delcomp_died} !=5 and ${symptoms_died} =1*	1 Doctor<br>2 Midwife<br>3 Nurse<br>4 Community health officer<br>5 Health extension worker<br>6 Trained TBA<br>7 Community health volunteer<br>8 Untrained TBA<br>9 Relative/friends<br>10 Nobody<br>11 Other
delassito_died	Please specify<br>*Question relevant when: ${healthfacwho_died} =11*	
Csec_died *(required)*	Was [p5_name_died] delivered by caesarean section, i.e., did they cut your belly open to take the baby out?<br>*Question relevant when: ${delplace_died} >2 and ${delplace_died} <9*	1 Yes<br>2 No
dried_died *(required)*	Was the baby wiped (dried) before the placenta was delivered?	1 Yes<br>2 No<br>9 Don't know<br>8 Not applicable
babyplaced_died *(required)*	Where was the baby placed immediately after delivery?	1 On the floor<br>2 On a cot<br>3 On the mother's abdomen<br>4 With someone else

Field	Question	Answer
		8 NA<br>9 DK<br>other Other
babyplaced_died_other	Specify other.<br>*Question relevant when: selected(${babyplaced_died}, 'other')*	
swaddle_died *(required)*	Was the baby wrapped with cloth before the placenta was delivered?	1 Yes<br>2 No<br>9 Don't know<br>8 Not applicable
cry_died	Did your baby cry or breathe easily immediately after birth?	1 Yes<br>2 No<br>9 Don't know<br>8 Not applicable
helpbreathe_died *(required)*	What was done to help the baby cry or breathe at the time of birth?<br>*Please do not read out responses. ASK: Anything else? Record all responses*<br>*Question relevant when: ${cry_died} !=1*	1 Rubbed/massaged<br>2 Dried<br>3 Mouth cleared<br>4 Nothing<br>8 NA<br>9 DK<br>other Other
helpbreathe_died_other	Specify other.<br>*Question relevant when: selected(${helpbreathe_died}, 'other')*	
breatheassist_died	Who took these measures to help the baby cry or breathe?<br>*Please do not read out responses.*<br>*Question relevant when: ${cry_died} !=1 and ${helpbreathe_died} !=4*	1 Doctor<br>2 Midwife<br>3 Nurse<br>4 Community health officer<br>5 Health extension worker<br>6 Trained TBA<br>7 Community health volunteer<br>8 Untrained TBA<br>9 Relative/friends<br>10 Nobody<br>11 Other
size_died *(required)*	When [p5_name_died] was born, was he/she very large, large, average, and smaller than average or very small?	1 Very large<br>2 Larger than average<br>3 Average<br>4 Smaller than average<br>5 Very small<br>9 Don't know
keepwarm_died *(required)*	What was done to keep [p5_name_died] warm after delivery (within the first day after birth)? Anything else?<br>*Multiple responses possible*	1 Dried the baby<br>2 Wrapped the baby with clean cloths<br>3 Put baby beside the mother

Field	Question	Answer
		4 Kept the baby on bare skin to skin contact<br>5 Bathed in warm water<br>6 Warmed delivery room<br>7 Nothing done<br>8 NA<br>9 DK<br>other Other
keepwarm_died_other	Specify other.<br>*Question relevant when: selected(${keepwarm_died}, 'other')*	
bftime_died *(required)*	How long after birth did you first put [p5_name_died] to the breast?<br>*If less than 1 hour, record '00' hours. If less than 24 hours, record hours. Otherwise, record days.*	1 Hours<br>2 Days<br>8 Never breastfed<br>9 DK
bfhours_died	Hours<br>*Question relevant when: ${bftime_died} =1*<br>*Response constrained to: .<24*	
bfdays_died	Days<br>*Question relevant when: ${bftime_died} =2*	
otherfood_died *(required)*	When did you first start giving food or drink other than breast milk to [p5_name_died]?<br>*Question relevant when: ${bftime_died} !=8*	1 Hours<br>2 Days<br>3 Months<br>4 Baby exclusively breastfed until now<br>8 NA<br>9 DK
otherfoodhours_died	Hours<br>*Question relevant when: ${otherfood_died} =1*<br>*Response constrained to: .<24*	
otherfooddays_died	Days<br>*Question relevant when: ${otherfood_died} =2*<br>*Response constrained to: .<32*	
otherfoodmonths_died *(required)*	Months<br>*Question relevant when: ${otherfood_died} =3*	
stillbreastfed_died *(required)*	Are you still breastfeeding [p5_name_died]?<br>*Question relevant when: ${bftime_died} !=8 and ${otherfood_died} !=4*	1 Yes<br>2 No<br>9 Don't know<br>8 Not applicable
bflength_died *(required)*	How long, in all, did you breastfeed [p5_name_died]?<br>*Question relevant when: ${bftime_died} !=8 and ${stillbreastfed_died} !=1 and ${otherfood_died} !=4*	1 Months<br>2 Until child died<br>8 NA<br>9 DK
bfmonths_died	Months<br>*Question relevant when: ${bflength_died} =1 and ${stillbreastfed_died} !=1*	
stopbf_died *(required)*	Why did you stop breastfeeding [p5_name_died]?<br>*Question relevant when: ${bftime_died} !=8 and ${stillbreastfed_died} !=1 and ${otherfood_died} !=4*	1 Mother ill/weak<br>2 Child ill/weak<br>3 Child died<br>4 Nipple/breast problem<br>5 Not enough milk<br>6 Mother working<br>7 Child refused<br>8 Weaning age<br>9 Became pregnant

Field	Question	Answer
		88 NA<br>other Other
stopbf_died_other	Specify other.<br>*Question relevant when: selected(${stopbf_died}, 'other')*	
facstay_died *(required)*	How long did you stay at the facility following your delivery?<br>*Question relevant when: ${delplace_died} !=1 and ${delplace_died} !=2*	1 Hours<br>2 Days<br>3 Months<br>8 NA<br>9 DK
fachr_died	Hours<br>*Question relevant when: ${facstay_died} =1*<br>*Response constrained to: .<24*	
facday_died	Days<br>*Question relevant when: ${facstay_died} =2*<br>*Response constrained to: .<32*	
facmo_died	Months<br>*Question relevant when: ${facstay_died} =3*	
Woman of the Household (1) > SECTION 2: REPRODUCTION > PREGNANCY HISTORY (1) > Born alive and died later > PNC for [p5_name_died]<br>*Group relevant when: ${U5_died} <60 and ${momPNCwkvisits_died} >1 or ${babyPNCtotvisits_died} >1*		
PNCfac_died *(required)*	Before you were discharged after [p5_name_died] was born, did any health care provider check on you or your newborn's health?<br>*Question relevant when: ${delplace_died} !=1 and ${delplace_died} !=2*	1 Yes<br>2 No
PNChome_died *(required)*	Before the Health Professional, CHO/CHN or traditional birth attendant left your house, after [p5_name_died] was born, did he/she check on your health?<br>*Question relevant when: ${delplace_died} =1 or ${delplace_died} =2*	1 Yes<br>2 No
PNCcheck_died *(required)*	Not including this first check, after [p5_name_died] was born, did any health care provider check on you or your baby's health in the first week?<br>*PROBE: Any visits where the health care provider or CHW came to your home after delivery to talk, provide counseling and examine yourself or your baby?*	1 Yes<br>2 No
momPNCtotvisits_died *(required)*	How many visits did YOU have in total following the delivery?<br>*PROBE: Any counseling given to the Mother for care of her baby is considered a visit for the baby*<br>*Question relevant when: ${PNCcheck_died} =1 and ${PNCfac_died} =1 or ${PNCfac_died} =3 or ${PNChome_died} =1 or ${PNChome_died} =3*	
babyPNCtotvisits_died *(required)*	How many visits did your BABY have in total following the delivery?<br>*PROBE: Any counseling given to the Mother for care of her baby is considered a visit for the baby*<br>*Question relevant when: ${PNCcheck_died} =1 and ${PNCfac_died} =2 or ${PNCfac_died} =3 or ${PNChome_died} =2 or ${PNChome_died} =3*	
momPNCwkvisits_died *(required)*	How many visits did YOU have in the first week following the delivery?<br>*PROBE: Any counseling given to the Mother for care of her baby is considered a visit for the baby*	

Field	Question	Answer
	Question relevant when: ${PNCcheck_died} =1 and ${momPNCtotvisits_died} >0<br>*Response constrained to: .< ${momPNCtotvisits_died}*	
babyPNCwkvisits_died *(required)*	How many visits did [p5_name_died] have in the first week following the delivery?<br>*PROBE: Any counseling given to the Mother for care of her baby is considered a visit for the baby*<br>*Question relevant when: ${PNCcheck_died} =1 and ${babyPNCtotvisits_died} >0*<br>*Response constrained to: .< ${babyPNCtotvisits_died}*	
momPNCtime_died *(required)*	After [p5_name_died] was born, how many hours, days or weeks after the birth did somebody first check on YOUR health?<br>*Question relevant when: ${PNCcheck_died} =1 and ${momPNCwkvisits_died} >0*	1 Hours<br>2 Days<br>3 Weeks<br>8 NA<br>9 DK
momPNChours_died	Hours<br>*Question relevant when: ${momPNCtime_died} =1*<br>*Response constrained to: .<24*	
momPNCdays_died	Days<br>*Question relevant when: ${momPNCtime_died} =2*<br>*Response constrained to: .<32*	
momPNCweeks_died	Weeks<br>*Question relevant when: ${momPNCtime_died} =3*	
babyPNCtime_died *(required)*	After [p5_name_died] was born, how many hours, days or weeks after the birth did somebody first check on your BABY's health?<br>*Question relevant when: ${PNCcheck_died} =1 and ${babyPNCwkvisits_died} >1*	1 Hours<br>2 Days<br>3 Weeks<br>8 NA<br>9 DK
babyPNChours_died	Hours<br>*Question relevant when: ${babyPNCtime_died} =1*<br>*Response constrained to: .<24*	
babyPNCdays_died	Days<br>*Question relevant when: ${babyPNCtime_died} =2*<br>*Response constrained to: .<32*	
babyPNCweeks_died	Weeks<br>*Question relevant when: ${babyPNCtime_died} =3*	
momPNCprov_died *(required)*	Who checked on YOUR health at that time? Anyone else?<br>*Question relevant when: ${PNCcheck_died} =1 and ${momPNCwkvisits_died} >0*	1 Doctor<br>2 Midwife<br>3 Nurse<br>4 Community health officer<br>5 Health extension worker<br>6 Trained TBA<br>7 Community health volunteer<br>8 Untrained TBA<br>9 Relative/friends<br>10 Nobody<br>11 Other<br>other Other
momPNCprov_died_other	Specify other.<br>*Question relevant when: selected(${momPNCprov_died}, 'other')*	
babyPNCprov_died *(required)*	Who checked on your [p5_name_died]'s health at that time? Anyone else?<br>*Question relevant when: ${PNCcheck_died} =1 and ${babyPNCwkvisits_died} >0*	1 Doctor<br>2 Midwife<br>3 Nurse<br>4 Community health officer<br>5 Health extension worker<br>6 Trained TBA<br>7 Community health volunteer<br>8 Untrained TBA<br>9 Relative/friends

Field	Question	Answer
		10 Nobody<br>11 Other<br>other Other
babyPNCprov_died_other	Specify other.<br>*Question relevant when: selected(${babyPNCprov_died}, 'other')*	
momPNCplace_died *(required)*	Who checked on YOUR health at that time? Anyone else?<br>*Probe to identify the type of source and circle the appropriate code. If unable to determine if a hospital, health center, or clinic is public or private medical, write the name of the place in 'other' field.*<br>*Question relevant when: ${PNCcheck_died} =1 and ${momPNCwkvisits_died} >1*	1 Your home<br>2 Other home<br>3 Govt. Hospital<br>4 Govt. Health center<br>5 CHPS compound<br>6 Govt. Health post<br>7 NGO Facility<br>8 Private facility<br>9 ON THE WAY TO FACILITY<br>other Other
momPNCplace_died_other	Specify other.<br>*Question relevant when: selected(${momPNCplace_died}, 'other')*	
babyPNCplace_died *(required)*	Who checked on [p5_name_died]'s health at that time? Anyone else?<br>*Probe to identify the type of source and circle the appropriate code. If unable to determine if a hospital, health center, or clinic is public or private medical, write the name of the place in 'other' field.*<br>*Question relevant when: ${PNCcheck_died} =1 and ${babyPNCwkvisits_died} >1*	1 Your home<br>2 Other home<br>3 Govt. Hospital<br>4 Govt. Health center<br>5 CHPS compound<br>6 Govt. Health post<br>7 NGO Facility<br>8 Private facility<br>9 ON THE WAY TO FACILITY<br>other Other
babyPNCplace_died_other	Specify other.<br>*Question relevant when: selected(${babyPNCplace_died}, 'other')*	
healthcheck_died *(required)*	What did the health worker/other do during that visit to check on YOUR health?<br>*Question relevant when: ${PNCcheck_died} =1 and ${momPNCwkvisits_died} >1*	1 Examined body<br>2 Checked breasts<br>3 Checked for heavy bleeding<br>4 Counseled on danger signs for newborns<br>5 Counseled on breastfeeding<br>6 Counseled on nutrition<br>8 NA<br>other Other
healthcheck_died_other	Specify other.<br>*Question relevant when: selected(${healthcheck_died}, 'other')*	

Woman of the Household (1) > SECTION 2: REPRODUCTION > PREGNANCY HISTORY (1) > Stillbirth or livebirth and death on the first day

Group relevant when: ${p3} =2

Field	Question	Answer
p2a_dead *(required)*	How many months did this pregnancy last?<br>*Response constrained to: .<13*	
Woman of the Household (1) > SECTION 2: REPRODUCTION > PREGNANCY HISTORY (1) > Stillbirth or livebirth and death on the first day > In what month and year did this pregnancy end?		
endpregmo *(required)*	In what month and year did this pregnancy end?	1 January<br>2 February<br>3 March<br>4 April<br>5 May<br>6 June<br>7 July<br>8 August<br>9 September<br>10 October<br>11 November<br>12 December
endpregyr *(required)*	Year<br>*YYYY*<br>*Response constrained to: .>1975 and .<2015*	
Woman of the Household (1) > SECTION 2: REPRODUCTION > PREGNANCY HISTORY (1) > Stillbirth or livebirth and death on the first day > ANC for stillbirth or livebirth<br>*Group relevant when: ${U5_dead} <60*		
pregintent_dead *(required)*	When you got pregnant with with this pregnancy, did you want to get pregnant at that time?	1 Yes<br>2 No<br>3 Don't Know
pregtiming_dead *(required)*	Did you want to have a baby later on or did you not want any more children?<br>*Question relevant when: ${pregintent_dead} !=1*	1 Later<br>2 No more<br>8 NA
wantedwait_dead *(required)*	How much longer did you want to wait?<br>*Question relevant when: ${pregintent_dead} !=1 and ${pregtiming_dead} !=2*	1 Months<br>2 Years<br>8 NA
wantedwait_mo_dead	Months<br>*Question relevant when: ${wantedwait_dead} =1*	
wantedwait_yr_dead	Years<br>*Question relevant when: ${wantedwait_dead} =2*	
ANCcheck_dead *(required)*	Did you see anyone for antenatal care during this pregnancy?	1 Yes<br>2 No
ANCprov_dead *(required)*	Whom did you see?<br>*Question relevant when: ${ANCcheck_dead} =1*	1 Doctor<br>2 Midwife<br>3 Nurse<br>4 Community health officer<br>5 Health extension worker<br>6 Traditional birth attendant<br>7 Other health worker<br>other Other
ANCprov_dead_other	Specify other.<br>*Question relevant when: selected(${ANCprov_dead}, 'other')*	
ANCplace_dead *(required)*	Where did you receive antenatal care for this pregnancy? Anywhere else?<br>*Probe to identify type(s) of source(s) and circle the appropriate code(s). If there is no appropriate code, select 'other', then enter the name of the place.*<br>*Question relevant when: ${ANCcheck_dead} =1*	1 Govt. Hospital<br>2 Govt. Health center/Clinic<br>3 CHPS compound<br>4 NGO facility<br>5 Private Clinic<br>6 Home<br>other Other
ANCplace_dead_other	Specify other.<br>*Question relevant when: selected(${ANCplace_dead}, 'other')*	
pinkbook_dead *(required)*	Have you ever had a maternal health book (pink book) for this pregnancy?<br>*Question relevant when: ${ANCcheck_dead} =1*	1 Yes, seen<br>2 Yes, not seen<br>3 No<br>8 NA
pinkbookANCvisits_dead	Enter number of visits from health book<br>*Question relevant when: ${pinkbook_dead} =1*	

Field	Question	Answer
ANC_mo_dead *(required)*	How many months pregnant were you when you first received antenatal care for this pregnancy?<br>*99=DK*<br>*Question relevant when: ${ANCcheck_dead} =1*	
firstprov_dead *(required)*	Who did you first receive antenatal care from during your pregnancy?<br>*Question relevant when: ${ANCcheck_dead} =1*	1 Doctor<br>2 Midwife<br>3 Nurse<br>4 Community health officer<br>5 Health extension worker<br>6 Traditional birth attendant<br>7 Other health worker
firstplace_dead *(required)*	Where did you first receive antenatal care from during your pregnancy?<br>*Question relevant when: ${ANCcheck_dead} =1*	1 Govt. Hospital<br>2 Govt. Health center/Clinic<br>3 CHPS compound<br>4 NGO facility<br>5 Private Clinic<br>6 Home
srANCvisits_dead *(required)*	How many times in total did you receive antenatal care during this pregnancy?<br>*99=DK*<br>*Question relevant when: ${ANCcheck_dead} =1*	
mismatch_dead	I notice that you mentioned receiving a number of ANC visits that is different than are listed in your book. Sometimes women may forget to bring their book to ANC; sometime the health worker may also not write in your book? Did this happen to you?<br>*Multiple answers allowed*<br>*Question relevant when: ${pinkbookANCvisits_dead} != ${srANCvisits_dead}*	1 Forgot to bring book<br>2 HW did not write<br>3 Other reason<br>other Other
mismatch_dead_other	Specify other.<br>*Question relevant when: selected(${mismatch_dead}, 'other')*	
Woman of the Household (1) > SECTION 2: REPRODUCTION > PREGNANCY HISTORY (1) > Stillbirth or livebirth and death on the first day > ANC for stillbirth or livebirth > duringpreg_dead<br>*Group relevant when: ${ANCcheck_dead} =1*		
generated_table_list_label_662	During this pregnancy:	
reserved_name_for_field_list_labels_663		1 Yes<br>2 No
pregweight_dead *(required)*	Were you weighed?<br>*Question relevant when: ${ANCcheck_dead} =1*	1 Yes<br>2 No
pregBP_dead *(required)*	Was your blood pressure measured?<br>*Question relevant when: ${ANCcheck_dead} =1*	1 Yes<br>2 No
pregheight_dead *(required)*	Was your height measured?<br>*Question relevant when: ${ANCcheck_dead} =1*	1 Yes<br>2 No
preguring_dead *(required)*	Did you give a urine sample?<br>*Question relevant when: ${ANCcheck_dead} =1*	1 Yes<br>2 No
pregblood_dead *(required)*	Did you give a blood sample?<br>*Question relevant when: ${ANCcheck_dead} =1*	1 Yes<br>2 No
pregstomach_dead *(required)*	Was your stomach measured?<br>*Question relevant when: ${ANCcheck_dead} =1*	1 Yes<br>2 No
pregHR_dead *(required)*	Did a health worker listen to the heart rate of your baby?<br>*Question relevant when: ${ANCcheck_dead} =1*	1 Yes<br>2 No
Woman of the Household (1) > SECTION 2: REPRODUCTION > PREGNANCY HISTORY (1) > Stillbirth or livebirth and death on the first day > ANC for stillbirth or livebirth > duringpregHW_dead<br>*Group relevant when: ${ANCcheck_dead} =1*		
generated_table_list_label_671	During this pregnancy, were you counseled by a health worker on:	
reserved_name_for_field_list_labels_672		1 Yes<br>2 No
pregfinprep_dead *(required)*	Financial preparation for your delivery?<br>*Question relevant when: ${ANCcheck_dead} =1*	1 Yes<br>2 No
pregbf_dead *(required)*	Breastfeeding immediately after delivery?<br>*Question relevant when: ${ANCcheck_dead} =1*	1 Yes<br>2 No
pregtt_dead *(required)*	Tetanus toxic vaccination?<br>*Question relevant when: ${ANCcheck_dead} =1*	1 Yes<br>2 No
pregdanger_dead *(required)*	Danger signs during delivery?<br>*Question relevant when: ${ANCcheck_dead} =1*	1 Yes<br>2 No
pregwrap_dead *(required)*	Wrapping the newborn after delivery?<br>*Question relevant when: ${ANCcheck_dead} =1*	1 Yes<br>2 No

Field	Question	Answer
pregSBA_dead *(required)*	Using a skilled birth attendant?<br>*Question relevant when: ${ANCcheck_dead} =1*	1 Yes<br>2 No
pregFP_dead *(required)*	Family planning?<br>*Question relevant when: ${ANCcheck_dead} =1*	1 Yes<br>2 No
pregtransport_dead *(required)*	Identifying emergency transport options?<br>*Question relevant when: ${ANCcheck_dead} =1*	1 Yes<br>2 No
pregdiet_died *(required)*	Diet?<br>*Question relevant when: ${ANCcheck_dead} =1*	1 Yes<br>2 No
pregother1_died *(required)*	Other<br>*Question relevant when: ${ANCcheck_dead} =1*	1 Yes<br>2 No
other_died	Please specify other things you were counseled on<br>*Question relevant when: ${pregother1_died} =1*	
dangersigns_dead *(required)*	During (any of) your antenatal care visit(s) during this pregnancy, were you told about the things to look out for that might suggest problems with the pregnancy?<br>*Question relevant when: ${ANCcheck_dead} =1*	1 Yes<br>2 No
dangerplace_dead *(required)*	Were you told where to go if you had any of these complications?<br>*Question relevant when: ${ANCcheck_dead} =1 and ${dangersigns_dead} =1*	1 Yes<br>2 No
pregbednet_dead *(required)*	At the time of this pregnancy, did your household have any bed net?	1 Yes<br>2 No<br>3 Don't Know
sleepfreq_dead *(required)*	How often did you sleep under a bed net during this pregnancy?<br>*Question relevant when: ${pregbednet_dead} !=2*	1 Every night<br>2 Most nights<br>3 Some nights<br>4 Rarely<br>5 Never<br>8 NA
pregmalaria_dead *(required)*	During this pregnancy, did you take any drugs to keep you from getting malaria?	1 Yes<br>2 No
pregmalariameds_dead	What drugs did you take?<br>*Question relevant when: ${pregmalaria_dead} =1*	1 SP<br>2 ACT<br>8 NA<br>9 DK<br>other Other
pregmalariameds_dead_other	Specify other.<br>*Question relevant when: selected(${pregmalariameds_dead}, 'other')*	
SPtimes_dead	How many times did you take SP?<br>*Enter 99 for DK*<br>*Question relevant when: ${pregmalariameds_dead} =1*	
ACTtimes_dead	How many times did you take ACT?<br>*Enter 99 for DK*<br>*Question relevant when: ${pregmalariameds_dead} =2*	
drugs_dead *(required)*	Did you get the drugs during any antenatal care visit, during another visit to a health facility or from another source?<br>*Question relevant when: ${pregmalariameds_dead} =1*	1 ANC Visit<br>2 Other health facility visit<br>3 Other source<br>8 NA
tetanus_dead *(required)*	During this pregnancy were you given an injection in the arm to prevent you and the baby from getting tetanus?	1 Yes<br>2 No
tetanustimes_dead *(required)*	During this pregnancy, how many times did you get this tetanus injection?<br>*Question relevant when: ${tetanus_dead} =1*	
previoustet_dead *(required)*	At any time before this pregnancy, did you receive any tetanus injections?	1 Yes<br>2 No<br>9 Don't know<br>8 Not applicable
prevtettimes_dead *(required)*	Before this pregnancy, how many tetanus injections did you receive in total?<br>*Question relevant when: ${previoustet_dead} =1*	
prevtetyr_dead *(required)*	How many years ago did you receive the last tetanus injection before this pregnancy?<br>*Question relevant when: ${previoustet_dead} =1*	
pregworm_dead *(required)*	During this pregnancy, did you take any drug for intestinal worms?	1 Yes<br>2 No
pregprep_dead	During this pregnancy, did you make any preparations for delivery?	1 Yes<br>2 No

Field	Question	Answer
Woman of the Household (1) > SECTION 2: REPRODUCTION > PREGNANCY HISTORY (1) > Stillbirth or livebirth and death on the first day > ANC for stillbirth or livebirth > preparation_dead<br>*Group relevant when: ${pregprep_dead} =1*		
generated_table_list_label_700	What kind of preparation did you make?<br>*Include all responses which the mother mentions unprompted. Then ask, "Is there anything else." Then, read each question and select "yes" or "no."*	
reserved_name_for_field_list_labels_701		1 Yes<br>2 No
preptransport_dead	Transport<br>*Question relevant when: ${pregprep_dead} =1*	1 Yes<br>2 No
prepmoney_dead	Money<br>*Question relevant when: ${pregprep_dead} =1*	1 Yes<br>2 No
prepfood_dead	Food<br>*Question relevant when: ${pregprep_dead} =1*	1 Yes<br>2 No
prepinstruments_dead	Clean instruments for delivery<br>*Question relevant when: ${pregprep_dead} =1*	1 Yes<br>2 No
prepcloths_dead	Clean cloths<br>*Question relevant when: ${pregprep_dead} =1*	1 Yes<br>2 No
prepother_dead	Other<br>*Question relevant when: ${pregprep_dead} =1*	1 Yes<br>2 No
prep_deadother	Please specify other preparations<br>*Question relevant when: ${pregprep_dead} =1 and ${prepother_dead} =1*	
delplan_dead	Did you discuss planning for your delivery with anybody while you were pregnant?	1 Yes<br>2 No
delplanwhom_dead	Whom did you plan your delivery with?<br>*Question relevant when: ${delplan_dead} =1*	1 Husband<br>2 Mother-in-law<br>3 Father-in-law<br>4 Friends/relative<br>5 CHN/CHO<br>6 Community Health volunteer<br>7 Other health worker<br>8 NA<br>9 DK<br>other Other
delplanwhom_dead_other	Specify other.<br>*Question relevant when: selected(${delplanwhom_dead}, 'other')*	
facdel_dead	Did any health worker give you specific instructions to go deliver at a health facility?	1 NONE<br>2 Doctor<br>3 Midwife<br>4 Nurse<br>5 Community health officer<br>6 Health extension worker<br>other Other
facdel_dead_other	Specify other.<br>*Question relevant when: selected(${facdel_dead}, 'other')*	
whenrec_dead	When did they make this recommendation?<br>*Question relevant when: ${facdel_dead} >3*	1 1st trimester<br>2 2nd trimester<br>3 3rd trimester, but before labor started<br>4 3rd trimester, during labor<br>8 NA<br>9 Don't know<br>other Other
whenrec_dead_other	Specify other.<br>*Question relevant when: selected(${whenrec_dead}, 'other')*	
whyrec_dead	Why did they make this recommendation?<br>*Question relevant when: ${facdel_dead} >3*	1 Does not know why<br>2 Suspected twins<br>3 Position of the baby<br>4 Hypertension/edema /blurred vision<br>5 Previous c-section<br>6 First birth

Field	Question	Answer
		7 Bleeding<br>8 Many hours in labor<br>9 Lack of movement of fetus<br>10 Diabetes<br>11 Anemia<br>88 NA<br>other Other
whyrec_dead_other	Specify other.<br>*Question relevant when: selected(${whyrec_dead}, 'other')*	
otherrec_dead	Did anyone else recommend that you go to deliver at health facility?	1 Husband<br>2 Mother-in-law<br>3 Mother<br>4 Friends/relative<br>5 No one else<br>other Other
otherrec_dead_other	Specify other.<br>*Question relevant when: selected(${otherrec_dead}, 'other')*	
delinstruct_dead	Did any health worker give you specific instructions to call him/her at the time of delivery?	1 NONE<br>2 Doctor<br>3 Midwife<br>4 Nurse<br>5 Community health officer<br>6 Health extension worker<br>other Other
delinstruct_dead_other	Specify other.<br>*Question relevant when: selected(${delinstruct_dead}, 'other')*	
Woman of the Household (1) > SECTION 2: REPRODUCTION > PREGNANCY HISTORY (1) > Stillbirth or livebirth and death on the first day > Delivering stillbirth or livebirth<br>*Group relevant when: ${U5_dead} <60*		
del_dead	Now I would like to ask you some questions about the delivery of your pregnancy and the care you may have received following his/her birth.	
delplace_dead *(required)*	Where did you give birth to this baby?<br>*Probe to identify the type of source and circle the appropriate code. If unable to determine if a hospital, health center…, write the name of the place in 'other' field*	1 Your home<br>2 Other home<br>3 Govt. Hospital<br>4 Govt. Health center<br>5 CHPS compound<br>6 Govt. Health post<br>7 NGO Facility<br>8 Private facility<br>9 ON THE WAY TO FACILITY<br>other Other
delplace_dead_other	Specify other.<br>*Question relevant when: selected(${delplace_dead}, 'other')*	
Woman of the Household (1) > SECTION 2: REPRODUCTION > PREGNANCY HISTORY (1) > Stillbirth or livebirth and death on the first day > Delivering stillbirth or livebirth > facreasons_dead<br>*Group relevant when: ${delplace_dead} =1 or ${delplace_dead} =2*		
generated_table_list_label_723	What was the reason you didn't deliver in a health facility?<br>*Do Not Read Out Responses PROBE: Any other reason? (Record all mentioned, and rank 1-9 according to importance).*	
expense_dead	Cost too much<br>*Question relevant when: ${delplace_dead} =1 or ${delplace_dead} =2*	
closed_dead	Facility not open<br>*Question relevant when: ${delplace_dead} =1 or ${delplace_dead} =2*	
transport_dead	Too far / no transportation<br>*Question relevant when: ${delplace_dead} =1 or ${delplace_dead} =2*	
trust_dead	Don't trust facility / poor quality service<br>*Question relevant when: ${delplace_dead} =1 or ${delplace_dead} =2*	
maleprov_dead	No female provider at facility<br>*Question relevant when: ${delplace_dead} =1 or ${delplace_dead} =2*	
autonomy_dead	Husband / family did not allow<br>*Question relevant when: ${delplace_dead} =1 or ${delplace_dead} =2*	
unnecessary_dead	Not necessary<br>*Question relevant when: ${delplace_dead} =1 or ${delplace_dead} =2*	

Field	Question	Answer
notcustomary_dead	Not customary<br>*Question relevant when: ${delplace_dead} =1 or ${delplace_dead} =2*	
other_fac_dead	Other<br>*Question relevant when: ${delplace_dead} =1 or ${delplace_dead} =2*	
state_otherdead	Please specify other reason<br>*Question relevant when: ${other_fac_dead} >0*	
Woman of the Household (1) > SECTION 2: REPRODUCTION > PREGNANCY HISTORY (1) > Stillbirth or livebirth and death on the first day > Delivering stillbirth or livebirth > homereasons_dead<br>*Group relevant when: ${delplace_dead} >2 and ${delplace_dead} <10*		
generated_table_list_label_735	What was the reason you didn't deliver at home?<br>*Do Not Read Out Responses PROBE: Any other reason? (Record all mentioned, and rank 1-9 according to importance).*	
complicate_dead	Experienced complications<br>*Question relevant when: ${delplace_dead} >2 and ${delplace_dead} <10*	
safety_dead	Facilities are safer<br>*Question relevant when: ${delplace_dead} >2 and ${delplace_dead} <10*	
free_dead	Free delivery at the facility<br>*Question relevant when: ${delplace_dead} >2 and ${delplace_dead} <10*	
access_dead	Access to the facility<br>*Question relevant when: ${delplace_dead} >2 and ${delplace_dead} <10*	
quality_dead	Better care at facilities<br>*Question relevant when: ${delplace_dead} >2 and ${delplace_dead} <10*	
other_home_dead	Other<br>*Question relevant when: ${delplace_dead} >2 and ${delplace_dead} <10*	
specify_otherdead	Please specify other reason<br>*Question relevant when: ${other_home_dead} >0*	
delcompwho_dead *(required)*	Who accompanied you to the health facility?<br>*Question relevant when: ${delplace_dead} >2 and ${delplace_dead} <10*	1 Nobody<br>2 Mother-in-law<br>3 Father-in-law<br>4 Husband<br>5 Other relative<br>6 CHO/CHN<br>9 DK<br>other Other
delcompwho_dead_other	Specify other.<br>*Question relevant when: selected(${delcompwho_dead}, 'other')*	
del_transportdead *(required)*	How did you travel to the health facility?<br>*Question relevant when: ${delplace_dead} >2 and ${delplace_dead} <9*	1 by foot (walking)<br>2 personal bicycle/motorbike /motoking<br>3 public transport (bus/taxi)<br>4 emergency motorking<br>5 ambulance<br>6 other
otherdel_transportdead *(required)*	Please specify transport used<br>*Question relevant when: ${del_transportdead} =6*	
homeassistwho_dead *(required)*	Who assisted with the delivery of this baby? Anyone else?<br>*Probe for the type(s) of person(s) and record all mentioned. If respondent says 'no one assisted', probe to determine whether any adults were present at the delivery.*	1 Doctor<br>2 Midwife<br>3 Nurse<br>4 Community health officer<br>5 Health extension worker<br>6 Trained TBA<br>7 Community health volunteer<br>8 Untrained TBA<br>9 Relative/friends<br>10 Nobody<br>11 Other
otherassist_dead	Please specify<br>*Question relevant when: ${homeassistwho_dead} =11*	
delcomp_dead *(required)*	During your most recent delivery, did you, personally, experience any of the following symptoms?	1 Convulsions<br>2 Long labor<br>3 Baby hand or feet coming first/abnormal position<br>4 Excessive bleeding/shock

Field	Question	Answer
		5 None<br>6 Others
othercomp_dead	Please specify other complications<br>*Question relevant when: ${delcomp_dead} =6*	
symptoms_dead *(required)*	When you experienced these symptoms, were you told to go to a health facility?<br>*Question relevant when: ${delcomp_dead} !=5*	1 Yes<br>2 Experienced symptoms while at a health facility<br>3 No<br>8 NA<br>9 DK
healthfacwho_dead	By whom?<br>*Question relevant when: ${delcomp_dead} !=5 and ${symptoms_dead} =1*	1 Doctor<br>2 Midwife<br>3 Nurse<br>4 Community health officer<br>5 Health extension worker<br>6 Trained TBA<br>7 Community health volunteer<br>8 Untrained TBA<br>9 Relative/friends<br>10 Nobody<br>11 Other
delassito_dead	Please specify<br>*Question relevant when: ${healthfacwho_dead} =11*	
Csec_dead *(required)*	Was this baby delivered by caesarean section, i.e., did they cut your belly open to take the baby out?<br>*Question relevant when: ${delplace_dead} >2 and ${delplace_dead} <9*	1 Yes<br>2 No
Woman of the Household (1) > SECTION 2: REPRODUCTION > PREGNANCY HISTORY (1) > Stillbirth or livebirth and death on the first day > PNC for still birth or live birth<br>*Group relevant when: ${U5_dead} <60 and ${momPNCwkvisits_dead} >1*		
PNCmomfac_dead *(required)*	Before you were discharged after this baby was born, did any health care provider check on your health?<br>*Question relevant when: ${delplace_dead} !=1 and ${delplace_dead} !=2*	1 Yes<br>2 No
PNCmomhome_dead	Before the Health Professional, CHO/CHN or traditional birth attendant left your house, after this baby was born, did he/she check on your health?<br>*Question relevant when: ${delplace_dead} =1 or ${delplace_dead} =2*	1 Yes<br>2 No
PNCmomcheck_dead	Not including this first check, after this baby was born, did any health care provider check on your health in the first week?<br>*PROBE: Any visits where the health care provider or CHW came to your home after delivery to talk, provide counseling and examine yourself or your baby?*	1 Yes<br>2 No
momPNCtotvisits_dead *(required)*	How many visits did YOU have in total following the delivery?<br>*PROBE: Any counseling given to the Mother for care of her baby is considered a visit for the baby*<br>*Question relevant when: ${PNCmomcheck_dead} =1 and ${PNCmomfac_dead} =1 or ${PNCmomfac_dead} =3 or ${PNCmomhome_dead} =1 or ${PNCmomhome_dead} =3*	
momPNCwkvisits_dead *(required)*	How many visits did YOU have in the first week following the delivery?<br>*PROBE: Any counseling given to the Mother for care of her baby is considered a visit for the baby*<br>*Question relevant when: ${PNCmomcheck_dead} =1 and ${momPNCtotvisits_dead} >0*<br>*Response constrained to: .< ${momPNCtotvisits_dead}*	
momPNCtime_dead *(required)*	After this baby was born, how many hours, days or weeks after the birth did somebody first check on YOUR health?<br>*Question relevant when: ${PNCmomcheck_dead} =1 and ${momPNCwkvisits_dead} >0*	1 Hours<br>2 Days<br>3 Weeks<br>8 NA<br>9 DK
momPNChours_dead	Hours<br>*Question relevant when: ${momPNCtime_dead} =1*<br>*Response constrained to: .<24*	
momPNCdays_dead	Days<br>*Question relevant when: ${momPNCtime_dead} =2*<br>*Response constrained to: .<32*	
momPNCweeks_dead	Weeks<br>*Question relevant when: ${momPNCtime_dead} =3*	
momPNCprov_dead *(required)*	Who checked on YOUR health at that time? Anyone else?<br>*Question relevant when: ${PNCmomcheck_dead} =1 and ${momPNCwkvisits_dead} >0*	1 Doctor<br>2 Midwife<br>3 Nurse<br>4 Community health officer<br>5 Health extension worker<br>6 Trained TBA

Field	Question	Answer
		7 Community health volunteer<br>8 Untrained TBA<br>9 Relative/friends<br>10 Nobody<br>11 Other<br>other Other
momPNCprov_dead_other	Specify other.<br>*Question relevant when: selected(${momPNCprov_dead}, 'other')*	
momPNCplace_dead *(required)*	Who checked on YOUR health at that time? Anyone else?<br>*Probe to identify the type of source and circle the appropriate code. If unable to determine if a hospital, health center, or clinic is public or private medical, write the name of the place in 'other' field.*<br>*Question relevant when: ${PNCmomcheck_dead} =1 and ${momPNCwkvisits_dead} >1*	1 Your home<br>2 Other home<br>3 Govt. Hospital<br>4 Govt. Health center<br>5 CHPS compound<br>6 Govt. Health post<br>7 NGO Facility<br>8 Private facility<br>9 ON THE WAY TO FACILITY<br>other Other
momPNCplace_dead_other	Specify other.<br>*Question relevant when: selected(${momPNCplace_dead}, 'other')*	
healthcheck_dead *(required)*	What did the health worker/other do during that visit to check on YOUR health?<br>*Question relevant when: ${PNCmomcheck_dead} =1 and ${momPNCwkvisits_dead} >1*	1 Examined body<br>2 Checked breasts<br>3 Checked for heavy bleeding<br>4 Counseled on danger signs for newborns<br>5 Counseled on breastfeeding<br>6 Counseled on nutrition<br>8 NA<br>other Other
healthcheck_dead_other	Specify other.<br>*Question relevant when: selected(${healthcheck_dead}, 'other')*	
Woman of the Household (1) > SECTION 2: REPRODUCTION > PREGNANCY HISTORY (1) > If lost<br>*Group relevant when: ${p3} =3*		
Woman of the Household (1) > SECTION 2: REPRODUCTION > PREGNANCY HISTORY (1) > If lost > In what month and year did you lose this pregnancy?		
datelostmo *(required)*	In what month and year did you lose this pregnancy?	1 January<br>2 February<br>3 March<br>4 April<br>5 May<br>6 June<br>7 July<br>8 August<br>9 September<br>10 October<br>11 November<br>12 December
datelostyr *(required)*	Year<br>*YYYY*<br>*Response constrained to: .>1975 and .<2015*	
Woman of the Household (1) > SECTION 2: REPRODUCTION > PREGNANCY HISTORY (1) > If lost > ANC for lost pregnancy<br>*Group relevant when: ${U5_lost} <60*		
pregintent_lost *(required)*	When you got pregnant with this pregnancy, did you want to get pregnant at that time?	1 Yes<br>2 No<br>3 Don't Know
pregtiming_lost *(required)*	Did you want to have a baby later on or did you not want any more children?<br>*Question relevant when: ${pregintent_lost} !=1*	1 Later<br>2 No more<br>8 NA

Field	Question	Answer
wantedwait_lost *(required)*	How much longer did you want to wait?<br>*Question relevant when: ${pregintent_lost} !=1 and ${pregtiming_lost} !=2*	1 Months<br>2 Years<br>8 NA
wantedwait_mo_lost	Months<br>*Question relevant when: ${wantedwait_lost} =1*	
wantedwait_yr_lost	Years<br>*Question relevant when: ${wantedwait_lost} =2*	
ANCcheck_lost *(required)*	Did you see anyone for antenatal care during this pregnancy?	1 Yes<br>2 No
ANCprov_lost *(required)*	Whom did you see?<br>*Question relevant when: ${ANCcheck_lost} =1*	1 Doctor<br>2 Midwife<br>3 Nurse<br>4 Community health officer<br>5 Health extension worker<br>6 Traditional birth attendant<br>7 Other health worker<br>other Other
ANCprov_lost_other	Specify other.<br>*Question relevant when: selected(${ANCprov_lost}, 'other')*	
ANCplace_lost *(required)*	Where did you first receive antenatal care for this pregnancy? Anywhere else?<br>*Probe to identify type(s) of source(s) and circle the appropriate code(s). If there is no appropriate code, select 'other', then enter the name of the place.*<br>*Question relevant when: ${ANCcheck_lost} =1*	1 Govt. Hospital<br>2 Govt. Health center/Clinic<br>3 CHPS compound<br>4 NGO facility<br>5 Private Clinic<br>6 Home<br>other Other
ANCplace_lost_other	Specify other.<br>*Question relevant when: selected(${ANCplace_lost}, 'other')*	
pinkbook_lost *(required)*	Have you ever had a maternal health book (pink book) for this pregnancy?<br>*Question relevant when: ${ANCcheck_lost} =1*	1 Yes, seen<br>2 Yes, not seen<br>3 No<br>8 NA
pinkbookANCvisits_lost *(required)*	Enter number of visits from health book<br>*Question relevant when: ${pinkbook_lost} =1*	
ANC_mo_lost *(required)*	How many months pregnant were you when you first received antenatal care for this pregnancy?<br>*Question relevant when: ${ANCcheck_lost} =1*	
firstprov_lost *(required)*	Who did you first receive antenatal care from during your pregnancy?<br>*Question relevant when: ${ANCcheck_lost} =1*	1 Doctor<br>2 Midwife<br>3 Nurse<br>4 Community health officer<br>5 Health extension worker<br>6 Traditional birth attendant<br>7 Other health worker
firstplace_lost *(required)*	Where did you first receive antenatal care from during your pregnancy?<br>*Question relevant when: ${ANCcheck_lost} =1*	1 Govt. Hospital<br>2 Govt. Health center/Clinic<br>3 CHPS compound<br>4 NGO facility<br>5 Private Clinic<br>6 Home<br>other Other
firstplace_lost_other	Specify other.<br>*Question relevant when: selected(${firstplace_lost}, 'other')*	
srANCvisits_lost	How many times in total did you receive antenatal care during this pregnancy?<br>*99=DK*<br>*Question relevant when: ${ANCcheck_lost} =1*	
mismatch_lost	I notice that you mentioned receiving a number of ANC visits that is different than are listed in your book. Sometimes women may forget to bring their book to ANC; sometime the health worker may also not write in your book? Did this happen to you?<br>*Multiple answers allowed*<br>*Question relevant when: ${pinkbookANCvisits_lost} != ${srANCvisits_lost}*	1 Forgot to bring book<br>2 HW did not write<br>3 Other reason<br>other Other
mismatch_lost_other	Specify other.<br>*Question relevant when: selected(${mismatch_lost}, 'other')*	

Field	Question	Answer
Woman of the Household (1) > SECTION 2: REPRODUCTION > PREGNANCY HISTORY (1) > If lost > ANC for lost pregnancy > duringpreg_lost<br>*Group relevant when: ${ANCcheck_lost} =1*		
generated_table_list_label_805	During this pregnancy:	
reserved_name_for_field_list_labels_806		1 Yes<br>2 No
pregweight_lost *(required)*	Were you weighed?<br>*Question relevant when: ${ANCcheck_lost} =1*	1 Yes<br>2 No
pregBP_lost *(required)*	Was your blood pressure measured?<br>*Question relevant when: ${ANCcheck_lost} =1*	1 Yes<br>2 No
pregheight_lost *(required)*	Was your height measured?<br>*Question relevant when: ${ANCcheck_lost} =1*	1 Yes<br>2 No
preguring_lost *(required)*	Did you give a urine sample?<br>*Question relevant when: ${ANCcheck_lost} =1*	1 Yes<br>2 No
pregblood_lost *(required)*	Did you give a blood sample?<br>*Question relevant when: ${ANCcheck_lost} =1*	1 Yes<br>2 No
pregstomach_lost *(required)*	Was your stomach measured?<br>*Question relevant when: ${ANCcheck_lost} =1*	1 Yes<br>2 No
pregHR_lost *(required)*	Did a health worker listen to the heart rate of your baby?<br>*Question relevant when: ${ANCcheck_lost} =1*	1 Yes<br>2 No
Woman of the Household (1) > SECTION 2: REPRODUCTION > PREGNANCY HISTORY (1) > If lost > ANC for lost pregnancy > duringpregHW_lost<br>*Group relevant when: ${ANCcheck_lost} =1*		
generated_table_list_label_814	During this pregnancy, were you counseled by a health worker on:	
reserved_name_for_field_list_labels_815		1 Yes<br>2 No
pregfinprep_lost	Financial preparation for your delivery?<br>*Question relevant when: ${ANCcheck_lost} =1*	1 Yes<br>2 No
pregbf_lost	Breastfeeding immediately after delivery?<br>*Question relevant when: ${ANCcheck_lost} =1*	1 Yes<br>2 No
pregtt_lost	Tetanus toxic vaccination?<br>*Question relevant when: ${ANCcheck_lost} =1*	1 Yes<br>2 No
pregdanger_lost	Danger signs during delivery?<br>*Question relevant when: ${ANCcheck_lost} =1*	1 Yes<br>2 No
pregwrap_lost	Wrapping the newborn after delivery?<br>*Question relevant when: ${ANCcheck_lost} =1*	1 Yes<br>2 No
pregSBA_lost	Using a skilled birth attendant?<br>*Question relevant when: ${ANCcheck_lost} =1*	1 Yes<br>2 No
pregFP_lost	Family planning?<br>*Question relevant when: ${ANCcheck_lost} =1*	1 Yes<br>2 No
pregtransport_lost	Identifying emergency transport options?<br>*Question relevant when: ${ANCcheck_lost} =1*	1 Yes<br>2 No
pregdiet_alive *(required)*	Diet?<br>*Question relevant when: ${ANCcheck_lost} =1*	1 Yes<br>2 No
pregother1_lost *(required)*	Other<br>*Question relevant when: ${ANCcheck_lost} =1*	1 Yes<br>2 No
other_lost	Please specify other things you were counseled on<br>*Question relevant when: ${pregother1_lost} =1*	
dangersigns_lost *(required)*	During (any of) your antenatal care visit(s) with this pregnancy, were you told about the things to look out for that might suggest problems with the pregnancy?<br>*Question relevant when: ${ANCcheck_lost} =1*	1 Yes<br>2 No
dangerplace_lost *(required)*	Were you told where to go if you had any of these complications?<br>*Question relevant when: ${ANCcheck_lost} =1 and ${dangersigns_lost} =1*	1 Yes<br>2 No
pregbednet_lost *(required)*	At the time of this pregnancy, did your household have any bed net?	1 Yes<br>2 No<br>3 Don't Know
sleepfreq_lost *(required)*	How often did you sleep under a bed net during this pregnancy?<br>*Question relevant when: ${pregbednet_lost} !=2*	1 Every night<br>2 Most nights<br>3 Some nights<br>4 Rarely<br>5 Never<br>8 NA

Field	Question	Answer
pregmalaria_lost *(required)*	During this pregnancy, did you take any drugs to keep you from getting malaria?	1 Yes<br>2 No
pregmalariameds_lost *(required)*	What drugs did you take?<br>*Question relevant when: ${pregmalaria_lost} =1*	1 SP<br>2 ACT<br>8 NA<br>9 DK<br>other Other
pregmalariameds_lost_other	Specify other.<br>*Question relevant when: selected(${pregmalariameds_lost}, 'other')*	
SPtimes_lost	How many times did you take SP?<br>*Enter 99 for DK*<br>*Question relevant when: ${pregmalariameds_lost} =1*	
ACTtimes_lost	How many times did you take ACT?<br>*Enter 99 for DK*<br>*Question relevant when: ${pregmalariameds_lost} =2*	
drugs_lost	Did you get the drugs during any antenatal care visit, during another visit to a health facility or from another source?<br>*Question relevant when: ${pregmalariameds_lost} =1*	1 ANC Visit<br>2 Other health facility visit<br>3 Other source<br>8 NA
tetanus_lost *(required)*	During this pregnancy were you given an injection in the arm to prevent you and the baby from getting tetanus?	1 Yes<br>2 No
tetanustimes_lost *(required)*	During this pregnancy, how many times did you get this tetanus injection?<br>*Question relevant when: ${tetanus_lost} =1*	
previoustet_lost *(required)*	At any time before this pregnancy, did you receive any tetanus injections?	1 Yes<br>2 No<br>9 Don't know<br>8 Not applicable
prevtettimes_lost *(required)*	Before this pregnancy, how many tetanus injections did you receive in total?<br>*Question relevant when: ${previoustet_lost} =1*	
prevtetyr_lost *(required)*	How many years ago did you receive the last tetanus injection before this pregnancy?<br>*Question relevant when: ${previoustet_lost} =1*	
pregworm_lost *(required)*	During this pregnancy, did you take any drug for intestinal worms?	1 Yes<br>2 No
pregprep_lost	During this pregnancy, did you make any preparations for delivery?	1 Yes<br>2 No
Woman of the Household (1) > SECTION 2: REPRODUCTION > PREGNANCY HISTORY (1) > If lost > ANC for lost pregnancy > preparation_lost<br>*Group relevant when: ${pregprep_lost} =1*		
generated_table_list_label_843	What kind of preparation did you make?<br>*Include all responses which the mother mentions unprompted. Then ask, "Is there anything else." Then, read each question and select "yes" or "no."*	
reserved_name_for_field_list_labels_844		1 Yes<br>2 No
preptransport_lost	Transport<br>*Question relevant when: ${pregprep_lost} =1*	1 Yes<br>2 No
prepmoney_lost	Money<br>*Question relevant when: ${pregprep_lost} =1*	1 Yes<br>2 No
prepfood_lost	Food<br>*Question relevant when: ${pregprep_lost} =1*	1 Yes<br>2 No
prepinstruments_lost	Clean instruments for delivery<br>*Question relevant when: ${pregprep_lost} =1*	1 Yes<br>2 No
prepcloths_lost	Clean cloths<br>*Question relevant when: ${pregprep_lost} =1*	1 Yes<br>2 No
prepother_lost	Other<br>*Question relevant when: ${pregprep_lost} =1*	1 Yes<br>2 No
delplan_lost	Did you discuss planning for your delivery with anybody while you were pregnant?	1 Yes<br>2 No
delplanwhom_lost	Whom did you plan your delivery with?<br>*Question relevant when: ${delplan_lost} =1*	1 Husband<br>2 Mother-in-law<br>3 Father-in-law<br>4 Friends/relative<br>5 CHN/CHO

Field	Question	Answer
		6 Community Health volunteer<br>7 Other health worker<br>8 NA<br>9 DK<br>other Other
delplanwhom_lost_other	Specify other.<br>*Question relevant when: selected(${delplanwhom_lost}, 'other')*	
facdel_lost	Did any health worker give you specific instructions to go deliver at a health facility?	1 NONE<br>2 Doctor<br>3 Midwife<br>4 Nurse<br>5 Community health officer<br>6 Health extension worker<br>other Other
facdel_lost_other	Specify other.<br>*Question relevant when: selected(${facdel_lost}, 'other')*	
whenrec_lost	When did they make this recommendation?<br>*Question relevant when: ${facdel_lost} >3*	1 1st trimester<br>2 2nd trimester<br>3 3rd trimester, but before labor started<br>4 3rd trimester, during labor<br>8 NA<br>9 Don't know<br>other Other
whenrec_lost_other	Specify other.<br>*Question relevant when: selected(${whenrec_lost}, 'other')*	
whyrec_lost	Why did they make this recommendation?<br>*Question relevant when: ${facdel_lost} >3*	1 Does not know why<br>2 Suspected twins<br>3 Position of the baby<br>4 Hypertension/edema /blurred vision<br>5 Previous c-section<br>6 First birth<br>7 Bleeding<br>8 Many hours in labor<br>9 Lack of movement of fetus<br>10 Diabetes<br>11 Anemia<br>88 NA<br>other Other
whyrec_lost_other	Specify other.<br>*Question relevant when: selected(${whyrec_lost}, 'other')*	
otherrec_lost	Did anyone else recommend that you go to deliver at health facility?	1 Husband<br>2 Mother-in-law<br>3 Mother<br>4 Friends/relative<br>5 No one else<br>other Other
otherrec_lost_other	Specify other.<br>*Question relevant when: selected(${otherrec_lost}, 'other')*	
delinstruct_lost	Did any health worker give you specific instructions to call him/her at the time of delivery?	1 NONE<br>2 Doctor<br>3 Midwife<br>4 Nurse<br>5 Community health officer<br>6 Health extension worker<br>other Other
delinstruct_lost_other	Specify other.<br>*Question relevant when: selected(${delinstruct_lost}, 'other')*	

Woman of the Household (1) > SECTION 3: CONTRACEPTION

Field	Question	Answer
generated_note_name_868	I would like to ask you some questions about the times you or your partner(s) may have used a method to avoid getting pregnant during the last few years.	
everused *(required)*	Have you ever used anything or tried in any way to delay or avoid getting pregnant?	1 Yes<br>2 No
evermethod *(required)*	What have you used or tried?<br>*PROBE AND RECORD ALL MENTIONED.*<br>*Question relevant when: ${everused} =1*	1 Female sterilization<br>2 Male sterilization<br>3 IUD<br>4 Injection<br>5 Implant<br>6 Pill<br>7 Condom<br>8 Female condom<br>9 Diaphragm<br>10 Foam/Jelly<br>11 Lactational amen method<br>12 Rhythm/Periodic abstinence<br>13 Withdrawal<br>14 Other modern method<br>15 Other traditional method<br>88 NA
Woman of the Household (1) > SECTION 3: CONTRACEPTION > In what month and year was the sterilization performed?<br>*Group relevant when: ${evermethod} =1 or ${evermethod} =2*		
sterile_mo	In what month and year was the sterilization performed?	1 January<br>2 February<br>3 March<br>4 April<br>5 May<br>6 June<br>7 July<br>8 August<br>9 September<br>10 October<br>11 November<br>12 December
sterile_yr	Year<br>*YYYY*<br>*Response constrained to: .>1965 and .<2015*	
FPcheck *(required)*	Are you or your husband/partner currently doing something or using any method to delay or avoid getting pregnant?<br>*Question relevant when: ${currpreg} !=1*	1 Yes<br>2 No<br>3 Don't Know
Cmethod *(required)*	Which method are you currently using?<br>*Question relevant when: ${currpreg} !=1 and ${FPcheck} !=2*	1 Female sterilization<br>2 Male sterilization<br>3 IUD<br>4 Injection<br>5 Implant<br>6 Pill<br>7 Condom<br>8 Female condom<br>9 Diaphragm<br>10 Foam/Jelly<br>11 Lactational amen method<br>12 Rhythm/Periodic abstinence<br>13 Withdrawal<br>14 Other modern method<br>15 Other traditional method<br>88 NA
obtaincheck *(required)*	Do you know of a place where you can obtain a method of family planning?	1 Yes<br>2 No
obtainmethod *(required)*	Where is that?<br>*MULTIPLE ANSWERS ALLOWED*<br>*Question relevant when: ${obtaincheck} !=2*	1 Govt. hospital<br>2 Govt. health center<br>3 Govt. Community Health Clinic (CHPS Zone)

Field	Question	Answer
		4 Mobile clinic<br>5 NGO facility<br>6 Other public<br>7 Private clinic<br>8 Pharmacy<br>9 Other private<br>10 Shop<br>11 Church<br>12 Friend/relative<br>88 NA<br>other Other
obtainmethod_other	Specify other.<br>*Question relevant when: selected(${obtainmethod}, 'other')*	
morekidspreg *(required)*	Now I have some questions about the future. After the child you are expecting now, would you like to have another child, or would you prefer not to have any more children?<br>*If currently pregnant*<br>*Question relevant when: ${currpreg} =1*	1 Have (a/another) child<br>2 No more/none<br>3 Says she can't get pregnant<br>9 Undecided/don't know
morekidsnopreg *(required)*	Now I have some questions about the future. Would you like to have (a/another) child, or would you prefer not to have any (more) children?<br>*If not currently pregnant*<br>*Question relevant when: ${currpreg} !=1*	1 Have (a/another) child<br>2 No more/none<br>3 Says she can't get pregnant<br>9 Undecided/don't know
wantsmorekidspreg *(required)*	After the birth of the child you are expecting now, how long would you like to wait before the birth of another child?<br>*If currently pregnant*<br>*Question relevant when: ${currpreg} =1 and ${morekidspreg} =1*	1 Specific number of months<br>2 Specific number of years<br>3 Soon/now<br>4 After marriage<br>5 DK<br>other Other
wantsmorekidspreg_other	Specify other.<br>*Question relevant when: selected(${wantsmorekidspreg}, 'other')*	
morekidspregmo *(required)*	Months<br>*Question relevant when: ${wantsmorekidspreg} =1*<br>*Response constrained to: .<12*	
morekidspregyr *(required)*	Years<br>*Question relevant when: ${wantsmorekidspreg} =2*	
wantsmorekidsnopreg *(required)*	How long would you like to wait from now before the birth of (a/another) child?<br>*If not currently pregnant*<br>*Question relevant when: ${currpreg} !=1 and ${morekidsnopreg} =1*	1 Specific number of months<br>2 Specific number of years<br>3 Soon/now<br>4 After marriage<br>5 DK<br>other Other
wantsmorekidsnopreg_other	Specify other.<br>*Question relevant when: selected(${wantsmorekidsnopreg}, 'other')*	
morekidsnopregmo *(required)*	Months<br>*if more than 12 months choose years*<br>*Question relevant when: ${wantsmorekidsnopreg} =1*<br>*Response constrained to: .<12*	
morekidsnopregyr	Years<br>*Question relevant when: ${wantsmorekidsnopreg} =2*	
anotherkidsoon *(required)*	You have said that you want (a/another) child soon. Can you tell me why you are not pregnant now?<br>*Question relevant when: ${wantsmorekidsnopreg} =3 and ${wantsmorekidspreg} !=3*	1 Not married<br>2 Not having sex<br>3 Infrequent sex<br>4 Menopausal/hysterectomy<br>5 Can't get pregnant<br>6 Not menstruated since last birth<br>7 Breastfeeding<br>8 Up to God<br>other Other
anotherkidsoon_other	Specify other.<br>*Question relevant when: selected(${anotherkidsoon}, 'other')*	
nomorekids *(required)*	You have said that you do not want any (more) children. Can you tell me why you are not using a method to prevent pregnancy?<br>*Question relevant when: ${morekidspreg} =2 or ${morekidsnopreg} =3*	1 Not married<br>2 Not having sex

Field	Question	Answer
		3 Infrequent sex<br>4 Menopausal/hysterectomy<br>5 Can't get pregnant<br>6 Not menstruated since last birth<br>7 Breastfeeding<br>8 Up to God<br>9 Respondent opposed<br>10 Partner opposed<br>11 Other opposed<br>12 Religion prohibition<br>13 Knows no method<br>14 Knows no source<br>15 Side-effects/health concerns<br>16 Lack of access/too far<br>17 Costs too much<br>18 Preferred method not available<br>19 Inconvenient to use<br>888 NA<br>other Other
nomorekids_other	Specify other.<br>*Question relevant when: selected(${nomorekids}, 'other')*	
futuremethod *(required)*	Do you think you will use a contraceptive method to delay or avoid pregnancy at any time in the future?<br>*Question relevant when: ${wantsmorekidspreg} !=3 and ${wantsmorekidsnopreg} !=3 and ${morekidsnopreg} !=3*	1 Yes<br>2 No<br>3 Up to God<br>8 NA<br>9 DK/Unsure
idealkidshaskids *(required)*	If you could go back to the time you did not have any children and could choose exactly the number of children to have in your whole life, how many would that be?<br>*Question relevant when: ${livewithkids} =1 or ${liveelsekids} =1*<br>*Response constrained to: .<21*	
idealkidshasnokids *(required)*	If you could choose exactly the number of children to have in your whole life, how many would that be?<br>*Question relevant when: ${livewithkids} !=1 and ${liveelsekids} !=1*<br>*Response constrained to: .<21*	
Woman of the Household (1) > SECTION 3: CONTRACEPTION > How many of these children would you like to be boys, how many would you like to be girls and for how many would it not matter if it's a boy or a girl?<br>*Group relevant when: ${idealkidshaskids} >0 or ${idealkidshasnokids} >0*		
hypoboys	Boys	
hypogirls	Girls	
hypoeither	Either	
Woman of the Household (1) > SECTION 3: CONTRACEPTION > FPmessages		
generated_table_list_label_901	In the last few months have you: Heard about family planning on the	
reserved_name_for_field_list_labels_902		1 Yes<br>2 No
FPradio *(required)*	Radio?	1 Yes<br>2 No
FPtv *(required)*	Television?	1 Yes<br>2 No
FPnews *(required)*	Newspaper?	1 Yes<br>2 No
FPnet *(required)*	Internet?	1 Yes<br>2 No
Fpmouth *(required)*	Word of mouth?	1 Yes<br>2 No
Woman of the Household (1) > SECTION 4: ANTENATAL CARE		
generated_note_name_911	Now I would like to ask you some questions about the ANC. I understand that you may not have attended ANC before but we would like to assess your knowledge of ANC.	
pregcomp *(required)*	What are the complications during pregnancy that need medical treatment?<br>*DO NOT READ OUT ANSWERS. Probe: Anything else?*	1 Severe Headache<br>2 Blurry Vision

Field	Question	Answer
		3 Reduced/absent fetal movement<br>4 High Blood Pressure<br>5 Edema of the face/hands<br>6 Convulsions<br>7 Excessive Vaginal Bleeding<br>8 Severe lower abdominal pain<br>9 Don't know/None mentioned<br>other Other
pregcomp_other	Specify other.<br>*Question relevant when: selected(${pregcomp}, 'other')*	
compmedtx *(required)*	What are the complications in a woman during childbirth that needs medical treatment?<br>*Please do not read out responses. ASK: Anything else? Record all responses.*	1 Excessive Vaginal Bleeding<br>2 Foul-Smelling Discharge<br>3 High Fever<br>4 Baby's Hand or Feet Come First<br>5 Baby in abnormal position<br>6 Prolonged Labor (>12 hours)<br>7 Retained Placenta<br>8 Rupture uterus<br>9 Prolapsed Cord<br>10 Cord around neck<br>11 Convulsions<br>99 Don't Know<br>other Other
compmedtx_other	Specify other.<br>*Question relevant when: selected(${compmedtx}, 'other')*	
Woman of the Household (1) > SECTION 5: MONTH AFTER BIRTH<br>*Group relevant when: ${numbirths} >0*		
generated_note_name_918	Now I would like to ask you some questions about your most recent delivery. In particular the care you received first one month after you delivered	
HWhome *(required)*	Have you been visited at home by a health worker?	1 Yes<br>2 No
HWwhen *(required)*	When was the last time that you were visited?<br>*Question relevant when: ${HWhome} =1*	1 Less than a week ago<br>2 A week ago<br>3 Two weeks ago<br>4 A month ago<br>5 More than a month ago<br>8 NA
HWtype *(required)*	What type of health worker visited you at the last time?<br>*Question relevant when: ${HWhome} =1*	1 Nurse/CHO<br>2 Trained TBA<br>3 Community health volunteer<br>4 Untrained TBA<br>8 NA<br>other Other
HWtype_other	Specify other.<br>*Question relevant when: selected(${HWtype}, 'other')*	
HWspeak *(required)*	Did this person speak with you the last time you were visited?<br>*Question relevant when: ${HWhome} =1*	1 Yes<br>2 No
HWtopic *(required)*	What did you speak about the last time you were visited?<br>*Question relevant when: ${HWhome} =1 and ${HWspeak} !=2*	1 Health of children<br>2 Health of adults<br>3 General sanitation<br>4 Disease prevention<br>5 Nutrition<br>6 Family Planning<br>7 Other Health topic<br>8 NA<br>other Other

Field	Question	Answer
HWtopic_other	Specify other.<br>*Question relevant when: selected(${HWtopic}, 'other')*	
HWservices *(required)*	Were any services provided to you or any of your children?<br>*Question relevant when: ${HWhome} =1 and ${HWspeak} !=2*	1 Yes<br>2 No<br>9 Don't know<br>8 Not applicable
HWservicetype *(required)*	What types of services were provided to you and any of your children?<br>*Question relevant when: ${HWhome} =1 and ${HWspeak} !=2 and ${HWservices} !=2*	1 Treatment for fever<br>2 Family planning services<br>3 Nutrition<br>4 Immunization<br>5 Treatment for diarrhoea<br>6 Treatment for cough<br>7 Treatment for skin rashes<br>8 NA<br>other Other
HWservicetype_other	Specify other.<br>*Question relevant when: selected(${HWservicetype}, 'other')*	
everfac *(required)*	Have you ever visited a health facility in this locality?	1 Yes<br>2 No<br>9 Don't know<br>8 Not applicable
HWrecentservice *(required)*	What types of services were provided to you during your most recent visits?<br>*Question relevant when: ${everfac} !=2*	1 Treatment for fever<br>2 Family planning services<br>3 Nutrition<br>4 Immunization<br>5 Treatment for diarrhoea<br>6 Treatment for cough<br>7 Treatment for skin rashes<br>8 NA<br>other Other
HWrecentservice_other	Specify other.<br>*Question relevant when: selected(${HWrecentservice}, 'other')*	
babydanger *(required)*	What are the danger signs/symptoms after giving birth indicating the need to seek health care for a baby less than a week old?<br>*When she has finished answering, ask "Is there anything else?"*	1 Fever<br>2 Unable to suckle/feed<br>3 Difficult/fast breathing<br>4 Diarrhea<br>5 Convulsions<br>6 Persistent vomiting<br>7 Yellow palms/soles /eyes/jaundice<br>8 Lethargy<br>9 Unconsciousness<br>10 Red/discharging eyes<br>11 Skin pustules<br>12 Skin around cord red<br>13 Pus from cord<br>14 Failure to pass urine<br>15 Shivering/cold baby/low temperature<br>16 Bluish palms and soles<br>17 Very small baby/below normal weight<br>18 Baby doesn't cry at birth<br>19 Baby cries stridently<br>20 None<br>other Other
babydanger_other	Specify other.<br>*Question relevant when: selected(${babydanger}, 'other')*	
everdanger *(required)*	Did [Name] experience any danger sign/symptoms during the first month following delivery?	1 Yes<br>2 No<br>9 Don't know<br>8 Not applicable

Field	Question	Answer
babyexp *(required)*	What were the danger signs/symptoms that [NAME] experienced?<br>*Question relevant when: ${everdanger} =1*	1 Fever<br>2 Unable to suckle/feed<br>3 Difficult/fast breathing<br>4 Diarrhea<br>5 Convulsions<br>6 Persistent vomiting<br>7 Yellow palms/soles /eyes/jaundice<br>8 Lethargy<br>9 Unconsciousness<br>10 Red/discharging eyes<br>11 Skin pustules<br>12 Skin around cord red<br>13 Pus from cord<br>14 Failure to pass urine<br>15 Shivering/cold baby/low temperature<br>16 Bluish palms and soles<br>17 Very small baby/below normal weight<br>18 Baby doesn't cry at birth<br>19 Baby cries stridently<br>20 None<br>other Other
babyexp_other	Specify other.<br>*Question relevant when: selected(${babyexp}, 'other')*	
numepisodes *(required)*	How many episodes of illness did [NAME/BABY] have up to the age of 1 month?<br>*For no episodes, enter 0.*	
numtreat *(required)*	How many times did you seek medical help up to the age of 1 month?<br>*Question relevant when: ${numepisodes} !=0*	
mostserious	If multiple danger signs were selected, what was the most serious episode that [NAME] experienced?<br>*IF MORE THAN ONE EPISODE OF ILLNESS, IDENTIFY WHAT MOTHER FELT WAS MOST SERIOUS EPISODE*<br>*Question relevant when: ${numepisodes} !=0*	1 Fever<br>2 Unable to suckle/feed<br>3 Difficult/fast breathing<br>4 Diarrhea<br>5 Convulsions<br>6 Persistent vomiting<br>7 Yellow palms/soles /eyes/jaundice<br>8 Lethargy<br>9 Unconsciousness<br>10 Red/discharging eyes<br>11 Skin pustules<br>12 Skin around cord red<br>13 Pus from cord<br>14 Failure to pass urine<br>15 Shivering/cold baby/low temperature<br>16 Bluish palms and soles<br>17 Very small baby/below normal weight<br>18 Baby doesn't cry at birth<br>19 Baby cries stridently<br>20 None
moserious	MOST SERIOUS ILLNESS: How old was [NAME] when the problem started?<br>*If less than 1 day, record hours. If less than 1 week, record days. Otherwise, record weeks.*<br>*Question relevant when: ${numepisodes} !=0*	1 Hours<br>2 Days<br>3 Weeks<br>8 NA<br>9 DK
moserioushrs	Hours<br>*Question relevant when: ${moserious} =1*	
moseriousdys	Days<br>*Question relevant when: ${moserious} =2*	

Field	Question	Answer
moseriouswks	Weeks<br>*Question relevant when: ${moserious} =3*	
Woman of the Household (1) > SECTION 5: MONTH AFTER BIRTH > How long after illness started was care initiated at home?		
athomecare	How long after illness started was care initiated at home?<br>*If less than 1 day, record hours. If less than 1 week, record days. Otherwise, record weeks.*<br>*Question relevant when: ${numepisodes} !=0*	1 Hours<br>2 Days<br>3 Weeks<br>8 NA<br>9 DK
athomehrs	Hours<br>*Question relevant when: ${athomecare} =1*	
athomedys	Days<br>*Question relevant when: ${athomecare} =2*	
athomewks	Weeks<br>*Question relevant when: ${athomecare} =3*	
everseek *(required)*	Did you seek advice or treatment for the illness outside the home?<br>*Question relevant when: ${numepisodes} !=0*	1 Yes<br>2 No<br>9 Don't know<br>8 Not applicable
outhomecare *(required)*	How much time after illness started was [NAME] brought outside the home for care?<br>*If less 30 minutes, enter 0 in hours. If 30 minutes or more enter 1 hour. If less than 1 day, record hours. If less than 1 week, record days. Otherwise, record weeks.*<br>*Question relevant when: ${numepisodes} !=0 and ${everseek} =1*	1 Hours<br>2 Days<br>3 Weeks<br>8 NA<br>9 DK
outhomehrs	Hours<br>*Question relevant when: ${outhomecare} =1*	
outhomedys	Days<br>*Question relevant when: ${outhomecare} =2*	
outhomewks	Weeks<br>*Question relevant when: ${outhomecare} =3*	
firstcarewho *(required)*	Whom did you go to for the first time for the problem?<br>*Question relevant when: ${numepisodes} !=0 and ${everseek} =1*	1 Doctor<br>2 Midwife<br>3 Nurse<br>4 Community health officer<br>5 Health extension worker<br>6 Trained TBA<br>7 Community health volunteer<br>8 Untrained TBA<br>9 Relative/friends<br>10 Nobody<br>11 Other<br>other Other
firstcarewho_other	Specify other.<br>*Question relevant when: selected(${firstcarewho}, 'other')*	
Woman of the Household (1) > SECTION 5: MONTH AFTER BIRTH > soughtcare<br>*Group relevant when: ${numepisodes} !=0 and ${everseek} =1*		
generated_table_list_label_950	From where did you seek care for the first time?	
reserved_name_for_field_list_labels_951		1 Yes<br>2 No
myhome *(required)*	Your home	1 Yes<br>2 No
othhome *(required)*	Other home	1 Yes<br>2 No
tradheal *(required)*	Traditional healer/ Healer	1 Yes<br>2 No
govthosp *(required)*	Government Hospital	1 Yes<br>2 No
govtHCfac *(required)*	Govt. Health Center facility	1 Yes<br>2 No
govtCHC *(required)*	Govt. Community Health Center (CHPS compound)	1 Yes<br>2 No

Field	Question	Answer
NGOfac *(required)*	NGO Facility	1 Yes<br>2 No
privfac *(required)*	Private facility	1 Yes<br>2 No
otherplace *(required)*	Other	1 Yes<br>2 No
nomedreason *(required)*	Why did you not seek medical help?<br>*Question relevant when: ${numepisodes} !=0 and ${everseek} !=1*	1 Expecting self resolution of the illness<br>2 Health facility too far/no transportation<br>3 Cost of treatment/service high<br>4 Don't trust facility/poor quality of care<br>5 Respected family members did not allow<br>6 The traditional birth attendant did not allow<br>7 Not customary to seek care outside home after childbirth<br>other Other
nomedreason_other	Specify other.<br>*Question relevant when: selected(${nomedreason}, 'other')*	
bffreq *(required)*	Now I would like to know how frequently your baby was breastfed during the illness. Was he/she breastfed less than usual, about the same or more than usual frequency?<br>*Question relevant when: ${numepisodes} !=0*	1 Less than usual<br>2 Same as usual<br>3 More than usual<br>4 Nothing to drink<br>9 Don't know
Woman of the Household (1) > SECTION 6: HEALTH-SEEKING BEHAVIORS<br>*Group relevant when: ${numbirths} >0*		
generated_note_name_966	Now I would like to ask you a few questions about the immunization of your children.	
novacc *(required)*	Who decides whether or not to immunize your children?<br>*This question will elicit anything other than a socially acceptable response*	1 Respondent<br>2 Husband/Partner<br>3 Respondent and spouse jointly<br>4 Mother-in-law<br>5 Father-in-law<br>6 Brother-in-law<br>7 Respondent and someone else<br>8 N/A – Decision never taken<br>other Other
novacc_other	Specify other.<br>*Question relevant when: selected(${novacc}, 'other')*	
vaccperm *(required)*	Do you need your husband's permission to go to a health facility for your child's immunization?	1 Yes<br>2 No
standperm *(required)*	Has your husband/partner given you standing permission to go to a health facility for any health needs of your child?<br>*Question relevant when: ${vaccperm} =2*	1 Yes<br>2 No
name *(required)*	Now, I would like to ask you a few questions about your last live-born child even if the child is no longer alive. Can you please remind me of the name of your last born child	
namestatus *(required)*	Check status: is [name] alive?	1 Yes<br>2 No
confirmage *(required)*	Is [name] at least 6 months but less than five years?<br>*If [name] is outside this age bracket, ask for the name of the next child who meets this cut-off*	1 Yes<br>2 No
generated_note_name_973	GO BACK TWO QUESTIONS AND CHANGE THE NAME OF CHILD TO NEW NAME PROVIDED. IF WOMEN HAS NO CHILD LESS THAN FIVE YEARS, SKIP THE REST OF THIS SECTION<br>*Question relevant when: ${confirmage} =2*	
immcard *(required)*	Is there a Child Health/Immunization card for [name]?<br>*IF YES: May I see it please?*	1 Yes, seen<br>2 Yes, not seen<br>3 No

Field	Question	Answer
BCGyes *(required)*	Does the card shows a BCG vaccination? *Do not read out this to woman*	1 Yes<br>2 No
Woman of the Household (1) > SECTION 6: HEALTH-SEEKING BEHAVIORS > Please enter dates for each immunization from the card BCG *Group relevant when: ${immcard} =1 and ${BCGyes} =1*		
BCGday *(required)*	Day *Enter 44 if card shows that vaccination was given but no date recorded* *Response constrained to: .<32 or .=44*	
BCGmo *(required)*	Month *Response constrained to: .<13*	
BCGyr *(required)*	Year *YYYY*	
polio0yes *(required)*	Does the card shows a polio0 vaccination? *Question relevant when: ${immcard} =1*	1 Yes<br>2 No
Woman of the Household (1) > SECTION 6: HEALTH-SEEKING BEHAVIORS > Polio at birth *Group relevant when: ${immcard} =1 and ${polio0yes} =1*		
polio0day *(required)*	Day *Enter 44 if card shows that vaccination was given but no date recorded* *Response constrained to: .<32 or .=44*	
polio0mo *(required)*	Month *Response constrained to: .<13*	
polio0yr *(required)*	Year *YYYY*	
polio1yes *(required)*	Does the card shows a polio1 vaccination? *Question relevant when: ${immcard} =1*	1 Yes<br>2 No
Woman of the Household (1) > SECTION 6: HEALTH-SEEKING BEHAVIORS > Polio 1 *Group relevant when: ${immcard} =1 and ${polio1yes} =1*		
polio1day *(required)*	Day *Enter 44 if card shows that vaccination was given but no date recorded* *Response constrained to: .<32 or .=44*	
polio1mo *(required)*	Month *Response constrained to: .<13*	
polio1yr *(required)*	Year *YYYY*	
polio2yes *(required)*	Does the card shows a polio2 vaccination? *Question relevant when: ${immcard} =1*	1 Yes<br>2 No
Woman of the Household (1) > SECTION 6: HEALTH-SEEKING BEHAVIORS > Polio 2 *Group relevant when: ${immcard} =1 and ${polio2yes} =1*		
polio2day *(required)*	Day *Enter 44 if card shows that vaccination was given but no date recorded* *Response constrained to: .<32 or .=44*	
polio2mo *(required)*	Month *Response constrained to: .<13*	
polio2yr *(required)*	Year *YYYY*	
polio3yes *(required)*	Does the card shows a polio3 vaccination? *Question relevant when: ${immcard} =1*	1 Yes<br>2 No
Woman of the Household (1) > SECTION 6: HEALTH-SEEKING BEHAVIORS > Polio 3 *Group relevant when: ${immcard} =1 and ${polio3yes} =1*		
polio3day *(required)*	Day *Enter 44 if card shows that vaccination was given but no date recorded* *Response constrained to: .<32 or .=44*	
polio3mo *(required)*	Month *Response constrained to: .<13*	
polio3yr *(required)*	Year *YYYY*	
dpt1yes *(required)*	Does the card shows a dpt1 vaccination? *Question relevant when: ${immcard} =1*	1 Yes<br>2 No
Woman of the Household (1) > SECTION 6: HEALTH-SEEKING BEHAVIORS > DPT 1 *Group relevant when: ${immcard} =1 and ${dpt1yes} =1*		
DPT1day *(required)*	Day *Enter 44 if card shows that vaccination was given but no date recorded* *Response constrained to: .<32 or .=44*	
DPT1mo *(required)*	Month *Response constrained to: .<13*	
DPT1yr *(required)*	Year *YYYY*	

Field	Question	Answer
dpt2yes *(required)*	Does the card shows a dpt2 vaccination?<br>*Question relevant when: ${immcard} =1*	1 Yes<br>2 No
Woman of the Household (1) > SECTION 6: HEALTH-SEEKING BEHAVIORS > DPT 2<br>*Group relevant when: ${immcard} =1 and ${dpt2yes} =1*		
DPT2day *(required)*	Day<br>*Enter 44 if card shows that vaccination was given but no date recorded*<br>*Response constrained to: .<32 or .=44*	
DPT2mo *(required)*	Month<br>*Response constrained to: .<13*	
DPT2yr *(required)*	Year<br>*YYYY*	
dpt3yes *(required)*	Does the card shows a dpt3 vaccination?<br>*Question relevant when: ${immcard} =1*	1 Yes<br>2 No
Woman of the Household (1) > SECTION 6: HEALTH-SEEKING BEHAVIORS > DPT 3<br>*Group relevant when: ${immcard} =1 and ${dpt3yes} =1*		
DPT3day *(required)*	Day<br>*Enter 44 if card shows that vaccination was given but no date recorded*<br>*Response constrained to: .<32 or .=44*	
DPT3mo *(required)*	Month<br>*Response constrained to: .<13*	
DPT3yr *(required)*	Year<br>*YYYY*	
measlesyes *(required)*	Does the card shows a measles vaccination?<br>*Question relevant when: ${immcard} =1*	1 Yes<br>2 No
Woman of the Household (1) > SECTION 6: HEALTH-SEEKING BEHAVIORS > Measles<br>*Group relevant when: ${immcard} =1 and ${measlesyes} =1*		
measlesday *(required)*	Day<br>*Enter 44 if card shows that vaccination was given but no date recorded*<br>*Response constrained to: .<32 or .=44*	
measlesmo *(required)*	Month<br>*Response constrained to: .<13*	
measlesyr *(required)*	Year<br>*YYYY*	
HEPb1yes *(required)*	Does the card shows a HEPb1 vaccination?<br>*Question relevant when: ${immcard} =1*	1 Yes<br>2 No
Woman of the Household (1) > SECTION 6: HEALTH-SEEKING BEHAVIORS > HEP B1<br>*Group relevant when: ${immcard} =1 and ${HEPb1yes} =1*		
HEPb1day *(required)*	Day<br>*Enter 44 if card shows that vaccination was given but no date recorded*<br>*Response constrained to: .<32 or .=44*	
HEPb1mo *(required)*	Month<br>*Response constrained to: .<13*	
HEPb1yr *(required)*	Year<br>*YYYY*	
HEPb2yes *(required)*	Does the card shows a HEPb2 vaccination?<br>*Question relevant when: ${immcard} =1*	1 Yes<br>2 No
Woman of the Household (1) > SECTION 6: HEALTH-SEEKING BEHAVIORS > HEP B2<br>*Group relevant when: ${immcard} =1 and ${HEPb2yes} =1*		
HEPb2day *(required)*	Day<br>*Enter 44 if card shows that vaccination was given but no date recorded*<br>*Response constrained to: .<32 or .=44*	
HEPb2mo *(required)*	Month<br>*Response constrained to: .<13*	
HEPb2yr *(required)*	Year<br>*YYYY*	
HEPb3yes *(required)*	Does the card shows a HEPb3 vaccination?<br>*Question relevant when: ${immcard} =1*	1 Yes<br>2 No
Woman of the Household (1) > SECTION 6: HEALTH-SEEKING BEHAVIORS > HEP B3<br>*Group relevant when: ${immcard} =1 and ${HEPb3yes} =1*		
HEPb3day *(required)*	Day<br>*Enter 44 if card shows that vaccination was given but no date recorded*<br>*Response constrained to: .<32 or .=44*	
HEPb3mo *(required)*	Month<br>*Response constrained to: .<13*	
HEPb3yr *(required)*	Year<br>*YYYY*	

Field	Question	Answer
Yellowfeveryes *(required)*	Does the card shows a yellow fever vaccination?<br>*Question relevant when: ${immcard} =1*	1 Yes<br>2 No
Woman of the Household (1) > SECTION 6: HEALTH-SEEKING BEHAVIORS > Yellow Fever<br>*Group relevant when: ${immcard} =1 and ${Yellowfeveryes} =1*		
yellowfeverday *(required)*	Day<br>*Enter 44 if card shows that vaccination was given but no date recorded*<br>*Response constrained to: .<32 or .=44*	
yellowfevermo *(required)*	Month<br>*Response constrained to: .<13*	
yellowfeveryr *(required)*	Year<br>*YYYY*	
VitA1yes *(required)*	Does the card shows a VitA1 vaccination?<br>*Question relevant when: ${immcard} =1*	1 Yes<br>2 No
Woman of the Household (1) > SECTION 6: HEALTH-SEEKING BEHAVIORS > Vitamin A1<br>*Group relevant when: ${immcard} =1 and ${VitA1yes} =1*		
vitA1day *(required)*	Day<br>*Enter 44 if card shows that vaccination was given but no date recorded*<br>*Response constrained to: .<32 or .=44*	
vitA1mo *(required)*	Month<br>*Response constrained to: .<13*	
vitA1yr *(required)*	Year<br>*YYYY*	
VitA2yes *(required)*	Does the card shows a VitA2 vaccination?<br>*Question relevant when: ${immcard} =1*	1 Yes<br>2 No
Woman of the Household (1) > SECTION 6: HEALTH-SEEKING BEHAVIORS > Vitamin A2<br>*Group relevant when: ${immcard} =1 and ${VitA2yes} =1*		
vitA2day *(required)*	Day<br>*Enter 44 if card shows that vaccination was given but no date recorded*<br>*Response constrained to: .<32 or .=44*	
vitA2mo *(required)*	Month<br>*Response constrained to: .<13*	
vitA2yr *(required)*	Year<br>*YYYY*	
otherimm *(required)*	Did [name] receive an immunization not recorded on this card, including immunization received during any campaigns (NIDs, SIA, Child health days or Measles campaigns)?<br>*Question relevant when: ${immcard} =1*	1 Yes<br>2 No<br>9 Don't know<br>8 Not applicable
otherimmreason *(required)*	Why was (were) this (these) immunization(s) not recorded?<br>*Question relevant when: ${otherimm} =1*	1 Forgot to bring book<br>2 HW did not write during NID/VACC campaign<br>3 HW did not write during facility visit<br>4 HW did not write during outreach/home visit<br>8 NA<br>other Other
otherimmreason_other	Specify other.<br>*Question relevant when: selected(${otherimmreason}, 'other')*	
Woman of the Household (1) > SECTION 6: HEALTH-SEEKING BEHAVIORS > Now I want to talk about any immunization not recorded on this card that [name] received.<br>*Group relevant when: ${otherimm} =1*		
othBCGyn	Did [name] receive BCG (Vaccination against tuberculosis): an injection in the arm or shoulder that usually causes a scar?<br>*Question relevant when: ${otherimm} =1*	1 Yes<br>2 No
othBCGx	Number of injections<br>*Question relevant when: ${otherimm} =1*	
othBCGtiming	Just after birth?<br>*Question relevant when: ${otherimm} =1*	1 Yes<br>2 No
Woman of the Household (1) > SECTION 6: HEALTH-SEEKING BEHAVIORS > Polio<br>*Group relevant when: ${otherimm} =1*		
othpolioyn	Did [name] receive Polio vaccine, that is, drops in the mouth?<br>*Question relevant when: ${otherimm} =1*	1 Yes<br>2 No
othpoliox	Number of times<br>*Question relevant when: ${otherimm} =1*	

Field	Question	Answer
othpoliotiming	Just after birth?<br>*Question relevant when: ${otherimm} =1*	1 Yes<br>2 No
Woman of the Household (1) > SECTION 6: HEALTH-SEEKING BEHAVIORS > DPT<br>*Group relevant when: ${otherimm} =1*		
othDPTyn	Did [name] receive DPT: an injection given in the right outer upper thigh, sometimes at the same time as polio drops?<br>*Question relevant when: ${otherimm} =1*	1 Yes<br>2 No
othDPTx	Number of injections<br>*Question relevant when: ${otherimm} =1*	
othDPTtiming	Just after birth?<br>*Question relevant when: ${otherimm} =1*	1 Yes<br>2 No
Woman of the Household (1) > SECTION 6: HEALTH-SEEKING BEHAVIORS > Measles<br>*Group relevant when: ${otherimm} =1*		
othmeaslesyn	Did [name] receive an injection to prevent measles?<br>*Question relevant when: ${otherimm} =1*	1 Yes<br>2 No
othmeaslesx	Number of injections<br>*Question relevant when: ${otherimm} =1*	
othmeaslestiming	Just after birth?<br>*Question relevant when: ${otherimm} =1*	1 Yes<br>2 No
Woman of the Household (1) > SECTION 6: HEALTH-SEEKING BEHAVIORS > Yellow Fever<br>*Group relevant when: ${otherimm} =1*		
othyellfeveryn	Did [name] receive yellow fever: an injection given in the upper right arm, often at the same time as measles vaccine?<br>*Question relevant when: ${otherimm} =1*	1 Yes<br>2 No
othyellfeverx	Number of injections<br>*Question relevant when: ${otherimm} =1*	
othyellfevertiming	Just after birth?<br>*Question relevant when: ${otherimm} =1*	1 Yes<br>2 No
othervacc *(required)*	Did [name] ever receive any immunization to prevent him/her from getting diseases, including immunization during a campaign (NIDs, SIA, or Measles campaign)?<br>*Question relevant when: ${immcard} !=1*	1 Yes<br>2 No
Woman of the Household (1) > SECTION 6: HEALTH-SEEKING BEHAVIORS > Now I want to talk about the immunization that [name] received.<br>*Group relevant when: ${immcard} !=1 and ${othervacc} =1*		
BCGnocardyn	Did [name] receive BCG (Vaccination against tuberculosis): an injection in the arm or shoulder that usually causes a scar?<br>*Question relevant when: ${immcard} !=1 and ${othervacc} =1*	1 Yes<br>2 No
BCGnocardx	Number of injections<br>*Question relevant when: ${immcard} !=1 and ${othervacc} =1*	
BCGnocardtiming	Just after birth?<br>*Question relevant when: ${immcard} !=1 and ${othervacc} =1*	1 Yes<br>2 No
Woman of the Household (1) > SECTION 6: HEALTH-SEEKING BEHAVIORS > Polio<br>*Group relevant when: ${immcard} !=1 and ${othervacc} =1*		
polionocardyn	Did [name] receive Polio vaccine, that is, drops in the mouth?<br>*Question relevant when: ${immcard} !=1 and ${othervacc} =1*	1 Yes<br>2 No
polionocardx	Number of times<br>*Question relevant when: ${immcard} !=1 and ${othervacc} =1*	
polionocardtiming	Just after birth?<br>*Question relevant when: ${immcard} !=1 and ${othervacc} =1*	1 Yes<br>2 No
Woman of the Household (1) > SECTION 6: HEALTH-SEEKING BEHAVIORS > DPT<br>*Group relevant when: ${immcard} !=1 and ${othervacc} =1*		
DPTnocardyn	Did [name] receive DPT: an injection given in the right outer upper thigh, sometimes at the same time as polio drops?<br>*Question relevant when: ${immcard} !=1 and ${othervacc} =1*	1 Yes<br>2 No
DPTnocardx	Number of injections<br>*Question relevant when: ${immcard} !=1 and ${othervacc} =1*	
DPTnocardtiming	Just after birth?<br>*Question relevant when: ${immcard} !=1 and ${othervacc} =1*	1 Yes<br>2 No
Woman of the Household (1) > SECTION 6: HEALTH-SEEKING BEHAVIORS > Measles<br>*Group relevant when: ${immcard} !=1 and ${othervacc} =1*		
measlesnocardyn	Did [name] receive an injection to prevent measles?<br>*Question relevant when: ${immcard} !=1 and ${othervacc} =1*	1 Yes<br>2 No

Field	Question	Answer
measlesnocardx	Number of injections<br>*Question relevant when: ${immcard} !=1 and ${othervacc} =1*	
measlesnocardtiming	Just after birth?<br>*Question relevant when: ${immcard} !=1 and ${othervacc} =1*	1 Yes<br>2 No
Woman of the Household (1) > SECTION 6: HEALTH-SEEKING BEHAVIORS > Yellow Fever<br>*Group relevant when: ${immcard} !=1 and ${othervacc} =1*		
yellfevernocardyn	Did [name] receive yellow fever: an injection given in the upper right arm, often at the same time as measles vaccine?<br>*Question relevant when: ${immcard} !=1 and ${othervacc} =1*	1 Yes<br>2 No
yellfevernocardx	Number of injections<br>*Question relevant when: ${immcard} !=1 and ${othervacc} =1*	
yellfevernocardtiming	Just after birth?<br>*Question relevant when: ${immcard} !=1 and ${othervacc} =1*	1 Yes<br>2 No
immsource *(required)*	From which sources did you receive immunization for [name]? Anywhere else?<br>*DO NOT READ LIST. RECORD ALL PLACES MENTIONED.*<br>*Question relevant when: ${immcard} !=1 and ${othervacc} =1*	1 Govt. Hospital<br>2 Govt. Health center/Clinic<br>3 CHPS compound<br>4 NGO facility<br>5 Private Clinic<br>6 Home<br>other Other
immsource_other	Specify other.<br>*Question relevant when: selected(${immsource}, 'other')*	
vacsource_other	Please specify<br>*Question relevant when: ${immsource} =7*	
evercampaign *(required)*	Did [name] receive any vaccine to prevent polio during National Immunization Days (NIDs), Supplementary Immunization Days or Immunization Plus Days (IPDs)?	1 Yes<br>2 No
Woman of the Household (1) > SECTION 6: HEALTH-SEEKING BEHAVIORS > campaign<br>*Group relevant when: ${evercampaign} =1*		
generated_table_list_label_1124	Did [name] participate in	
reserved_name_for_field_list_labels_1125		1 Yes<br>2 No<br>3 NA
NID *(required)*	Polio mass campaign (NID) of September 2014	1 Yes<br>2 No<br>3 NA
IPD *(required)*	Child health days of May 2014	1 Yes<br>2 No<br>3 NA
unabletovacc *(required)*	Have you ever taken [name] to a health facility for immunization and he/she was not able to receive the vaccine?	1 Yes<br>2 No<br>9 Don't know<br>8 Not applicable
novaccreason *(required)*	Why was [name] not able to receive the vaccine on that (those) occasion(s)?<br>*Question relevant when: ${unabletovacc} =1*	1 Lack of vaccines<br>2 Long waiting times<br>3 Negative attitude of provider<br>4 Did not have enough money<br>other Other
novaccreason_other	Specify other.<br>*Question relevant when: selected(${novaccreason}, 'other')*	
Woman of the Household (1) > SECTION 6: HEALTH-SEEKING BEHAVIORS > immprov		
generated_table_list_label_1130	The last time you took [name] to a health facility for immunization, did the immunization provider	
reserved_name_for_field_list_labels_1131		1 Yes<br>2 No<br>3 Don't Know
immimport *(required)*	Explain the importance of immunization?	1 Yes<br>2 No<br>3 Don't Know
immrecord *(required)*	Fill out the child health card (immunization record card)?	1 Yes<br>2 No<br>3 Don't Know

Field	Question	Answer
immnext *(required)*	Explain when to return for next immunization?	1 Yes<br>2 No<br>3 Don't Know
immeffect *(required)*	Explain common side effects of the vaccine?	1 Yes<br>2 No<br>3 Don't Know
immeffectact *(required)*	Explain what to do about common side effects?	1 Yes<br>2 No<br>3 Don't Know
Woman of the Household (1) > SECTION 7. VITAMIN A – CHILDREN 6 MONTHS AND OLDER<br>*Group relevant when: ${numbirths} >0*		
evervitA *(required)*	Has [name] ever received a vitamin A capsule (supplement) like this one?<br>*SHOW CAPSULES: 100,000 IU FOR THOSE 6-11 MONTHS OLD, (BLUE) 200,000 IU FOR THOSE 12-59 MONTHS OLD. (RED).*	1 Yes<br>2 No<br>9 Don't know<br>8 Not applicable
lastdosemo *(required)*	How many months ago did [name] take the last dose?<br>*Question relevant when: ${evervitA} =1*	
lastdoseplace *(required)*	Where did [name] get this last dose?<br>*Question relevant when: ${evervitA} =1*	1 On routine visit to health facility/CHPS<br>2 Sick child visit to health facility<br>3 National Immunization Day campaign<br>4 Child health week<br>5 Outreach clinics<br>8 NA<br>9 DK<br>other Other
lastdoseplace_other	Specify other.<br>*Question relevant when: selected(${lastdoseplace}, 'other')*	
freq12mo *(required)*	How many times did [name] receive capsule(s) in the last 12 months?<br>*Question relevant when: ${evervitA} =1*	
Woman of the Household (1) > SECTION 8: BREASTFEEDING<br>*Group relevant when: ${numbirths} >0*		
generated_note_name_1150	Now I would like to ask you a few questions about breastfeeding for this child.	
everbf *(required)*	Has [name] ever been breastfed?	1 Yes<br>2 No
stillbf *(required)*	Is [name] still being breastfed?<br>*Question relevant when: ${everbf} =1 and ${namestatus} =1*	1 Yes<br>2 No
monthsbf	For how many months did you breastfeed [name]?<br>*Question relevant when: ${everbf} =1 and ${stillbf} !=9*	
bfyesterday	Was [name] breastfed yesterday?<br>*Question relevant when: ${everbf} =1 and ${stillbf} =1*	1 Yes<br>2 No
Woman of the Household (1) > SECTION 8: BREASTFEEDING > feeding		
generated_table_list_label_1155	Since this time yesterday, did he/she receive any of the following:	
reserved_name_for_field_list_labels_1156		1 Yes<br>2 No<br>9 Don't know<br>8 Not applicable
vitA *(required)*	Vitamin, mineral supplements (Abidec, Minadex, etc)?	1 Yes<br>2 No<br>9 Don't know<br>8 Not applicable
water *(required)*	Plain water?	1 Yes<br>2 No<br>9 Don't know<br>8 Not applicable
juice *(required)*	Sweetened, flavoured water or fruit juice or tea or infusion?	1 Yes<br>2 No<br>9 Don't know<br>8 Not applicable

Field	Question	Answer
ors *(required)*	ORS?	1 Yes<br>2 No<br>9 Don't know<br>8 Not applicable
formula *(required)*	Infant formula (e.g. SMA, Lactogen)?	1 Yes<br>2 No<br>9 Don't know<br>8 Not applicable
milk *(required)*	Tinned, powdered or fresh milk?	1 Yes<br>2 No<br>9 Don't know<br>8 Not applicable
otherliq *(required)*	Any other liquids (e.g. coconut water, flour water, or other locally made liquid)?	1 Yes<br>2 No<br>9 Don't know<br>8 Not applicable
food *(required)*	Solid or semi-solid (mushy) food?	1 Yes<br>2 No<br>9 Don't know<br>8 Not applicable
semisolid *(required)*	Since this time yesterday, how many times did [name] eat solid, semisolid, or soft foods other than liquids? IF 7 OR MORE TIMES, RECORD '7'.<br>*DK = 9*<br>*Question relevant when: ${food} =1*<br>*Response constrained to: .<8 or .=9*	
Woman of the Household (1) > SECTION 9: CARE OF ILLNESS<br>*Group relevant when: ${numbirths} >0*		
generated_note_name_1170	Now I would like to ask you a few questions about the health of [name]	
diarrhoea *(required)*	Has [name] had diarrhoea in the last two weeks?<br>*DIARRHOEA IS DETERMINED AS PERCEIVED BY MOTHER OR CARETAKER, OR AS THREE OR MORE LOOSE OR WATERY STOOLS PER DAY, OR BLOOD IN STOOL.*	1 Yes<br>2 No<br>3 NA
Woman of the Household (1) > SECTION 9: CARE OF ILLNESS > solution<br>*Group relevant when: ${diarrhoea} =1*		
generated_table_list_label_1173	During this last episode of diarrhoea, did [name] drink any of the following:<br>*READ EACH ITEM ALOUD AND RECORD RESPONSE BEFORE PROCEEDING TO THE NEXT ITEM.*	
reserved_name_for_field_list_labels_1174		1 Yes<br>2 No<br>3 Don't Know
ORS *(required)*	Fluid from ORS<br>*Question relevant when: ${diarrhoea} =1*	1 Yes<br>2 No<br>3 Don't Know
fluid *(required)*	Homemade fluid<br>*Question relevant when: ${diarrhoea} =1*	1 Yes<br>2 No<br>3 Don't Know
ORSplace *(required)*	Where did you get the (ORS PACKET)?<br>*Question relevant when: ${diarrhoea} =1 and ${ORS} =1*	1 Govt. hospital/polyclinic<br>2 Govt. health centre<br>3 CHPS compound<br>4 Village health worker<br>5 Mobile/outreach clinic<br>6 Other public<br>7 Private hospital/clinic<br>8 Private physician<br>9 Private pharmacy<br>10 Mobile clinic<br>11 Other private medical<br>12 Relative or friend<br>13 Shop<br>14 Traditional practitioner<br>88 NA<br>99 DK<br>other Other

Field	Question	Answer
ORSplace_other	Specify other.<br>*Question relevant when: selected(${ORSplace}, 'other')*	
ORSpayment *(required)*	How did you pay for (ORS PACKET)?<br>*Question relevant when: ${diarrhoea} =1 and ${ORS} =1*	1 Ghanaian currency<br>2 NHIS Beneficiary<br>3 Free<br>4 NA<br>9 DK
Woman of the Household (1) > SECTION 9: CARE OF ILLNESS > How much?<br>*Group relevant when: ${ORSpayment} =1 and ${diarrhoea} =1 and ${ORS} =1*		
ORScedi	Cedis<br>*Question relevant when: ${ORSpayment} =1 and ${diarrhoea} =1 and ${ORS} =1*	
ORSgp	Pesawas<br>*Question relevant when: ${ORSpayment} =1 and ${diarrhoea} =1 and ${ORS} =2*<br>*Response constrained to: .<100*	
volume *(required)*	During [name]'s illness, did he/she drink much less, about the same, or more than usual?<br>*Question relevant when: ${diarrhoea} =1*	1 Much less or none<br>2 About the same (or somewhat less)<br>3 More<br>8 NA<br>9 DK
foodamt *(required)*	During [name]'s illness, did he/she eat less, about the same, or more food than usual?<br>*IF "LESS", PROBE: much less or a little less?*<br>*Question relevant when: ${diarrhoea} =1*	1 None<br>2 Much less<br>3 Somewhat less<br>4 About the same<br>5 More<br>8 NA<br>9 DK
cough *(required)*	Has [name] been ill with a cough at any time in the last two weeks?<br>*IF [name] DIED MORE THAN TWO WEEKS AGO, DO NOT ASK THIS QUESTION, SELECT NO AND MOVE TO THE NEXT QUESTION*	1 Yes<br>2 No<br>3 NA
rapidbreath *(required)*	When [name] had an illness with a cough, did he/she breathe faster than usual with short, quick breaths or have difficulty breathing?<br>*Question relevant when: ${cough} =1*	1 Yes<br>2 No<br>3 Don't Know
symptomsdue *(required)*	Were the symptoms due to a problem in the chest or a blocked nose?<br>*Question relevant when: ${rapidbreath} =1 and ${cough} =1*	1 Problem in chest<br>2 Blocked nose<br>3 Both<br>4 Other<br>8 NA<br>9 DK
treatment *(required)*	Did you seek advice or treatment for the illness outside the home?<br>*Question relevant when: ${cough} =1 and ${symptomsdue} !=4*	1 Yes<br>2 No
care *(required)*	From where did you seek care? Anywhere else?<br>*CIRCLE ALL PROVIDERS MENTIONED, BUT DO NOT PROMPT WITH ANY SUGGESTIONS. IF SOURCE IS HOSPITAL, HEALTH CENTER, OR CLINIC, WRITE THE NAME OF THE PLACE BELOW. PROBE TO IDENTIFY THE TYPE OF SOURCE AND CIRCLE THE APPROPRIATE CODE.*<br>*Question relevant when: ${cough} =1 and ${symptomsdue} !=4 and ${treatment} =1*	1 Govt. hospital/polyclinic<br>2 Govt. health centre<br>3 CHPS compound<br>4 Village health worker<br>5 Mobile/outreach clinic<br>6 Other public<br>7 Private hospital/clinic<br>8 Private physician<br>9 Private pharmacy<br>10 Mobile clinic<br>11 Other private medical<br>12 Relative or friend<br>13 Shop<br>14 Traditional practitioner<br>88 NA<br>99 DK<br>other Other
care_other	Specify other.<br>*Question relevant when: selected(${care}, 'other')*	
meds *(required)*	Was [name] given medicine to treat this illness?<br>*Question relevant when: ${cough} =1 and ${symptomsdue} !=4*	1 Yes<br>2 No

Field	Question	Answer
whichmeds *(required)*	What medicine was [name] given?<br>*INCLUDE ALL MEDICINES GIVEN.*<br>*Question relevant when: ${cough} =1 and ${symptomsdue} !=4 and ${meds} =1*	1 Antibiotic<br>2 Paracetamol/Panadol /Acetaminophen<br>3 Aspirin<br>4 Ibuprofen<br>8 NA<br>9 DK<br>other Other
whichmeds_other	Specify other.<br>*Question relevant when: selected(${whichmeds}, 'other')*	
antiplace *(required)*	Where did you get the antibiotic?<br>*Question relevant when: ${cough} =1 and ${symptomsdue} !=4 and ${whichmeds} =1*	1 Govt. hospital/polyclinic<br>2 Govt. health centre<br>3 CHPS compound<br>4 Village health worker<br>5 Mobile/outreach clinic<br>6 Other public<br>7 Private hospital/clinic<br>8 Private physician<br>9 Private pharmacy<br>10 Mobile clinic<br>11 Other private medical<br>12 Relative or friend<br>13 Shop<br>14 Traditional practitioner<br>88 NA<br>99 DK<br>other Other
antiplace_other	Specify other.<br>*Question relevant when: selected(${antiplace}, 'other')*	
antipayment *(required)*	How much did you pay for the antibiotic?<br>*Question relevant when: ${cough} =1 and ${symptomsdue} !=4 and ${whichmeds} =1*	1 Ghanaian currency<br>2 NHIS Beneficiary<br>3 Free<br>4 NA<br>9 DK
Woman of the Household (1) > SECTION 9: CARE OF ILLNESS > How much?<br>*Group relevant when: ${cough} =1 and ${antipayment} =1 and ${whichmeds} =1 and ${symptomsdue} !=4*		
antibioticcedi	Cedis	
antibioticGp	Pesawas<br>*Response constrained to: .<100*	
paid_consult	Did you pay for consultation?<br>*Question relevant when: ${cough} =1 and ${treatment} =1*	1 Yes<br>2 No<br>3 Don't Know
consultpayment	How did you have to pay for consultation?<br>*Question relevant when: ${cough} =1 and ${symptomsdue} !=4 and ${whichmeds} =1*	1 Ghanaian currency<br>2 NHIS Beneficiary<br>3 Free<br>4 NA<br>9 DK
Woman of the Household (1) > SECTION 9: CARE OF ILLNESS > How much did you pay for the antibiotic?<br>*Group relevant when: ${cough} =1 and ${consultpayment} =1 and ${symptomsdue} !=4 and ${whichmeds} =1*		
consultcedi	Cedis	
consultGp	Pesawas<br>*Response constrained to: .<100*	
transportpayment	How much did you pay for transportation to and from the facility for you and other person with you?<br>*Question relevant when: ${cough} =1 and ${symptomsdue} !=4 and ${treatment} =1*	1 Ghanaian currency<br>2 NHIS Beneficiary<br>3 Free<br>4 NA<br>9 DK
Woman of the Household (1) > SECTION 9: CARE OF ILLNESS > How much did you pay for transportation to and from the facility for you and other person with you?<br>*Group relevant when: ${cough} =1 and ${transportpayment} =1 and ${treatment} =1 and ${symptomsdue} !=4*		
transportcedi	Cedis	

Field	Question	Answer
foodpayment	How did you pay for food and drinks for you and other persons with you?<br>*Question relevant when: ${cough} =1 and ${symptomsdue} !=4 and ${treatment} =1*	1 Ghanaian currency<br>2 NHIS Beneficiary<br>3 Free<br>4 NA<br>9 DK
Woman of the Household (1) > SECTION 9: CARE OF ILLNESS > How much did you pay for food and drinks for you and other person with you?		
foodcedi	Cedis<br>*Question relevant when: ${cough} =1 and ${foodpayment} =1 and ${treatment} =1 and ${symptomsdue} !=4*	
traveltime *(required)*	How long did you have to travel to and from the facility?<br>*Question relevant when: ${cough} =1 and ${symptomsdue} !=4 and ${treatment} =1*	1 Hours<br>2 Minutes<br>8 NA<br>9 DK
travel_hr	Hours<br>*Question relevant when: ${cough} =1 and ${traveltime} =1 and ${treatment} =1 and ${symptomsdue} !=4*	
travel_min	Minutes<br>*Question relevant when: ${cough} =1 and ${traveltime} =2 and ${treatment} =1 and ${symptomsdue} !=4*<br>*Response constrained to: .<60*	
waittime *(required)*	How long did you have to wait at the facility before you were seen by health personnel?<br>*Question relevant when: ${cough} =1 and ${symptomsdue} !=4 and ${treatment} =1*	1 Hours<br>2 Minutes<br>8 NA<br>9 DK
wait_hr	Hours<br>*Question relevant when: ${cough} =1 and ${waittime} =1 and ${treatment} =1 and ${symptomsdue} !=4*	
wait_min	Minutes<br>*Question relevant when: ${cough} =1 and ${waittime} =2 and ${treatment} =1 and ${symptomsdue} !=4*<br>*Response constrained to: .<60*	
undonework *(required)*	What work did you have to leave undone to attend services at the facility?<br>*Question relevant when: ${cough} =1 and ${symptomsdue} !=4 and ${treatment} =1*	1 Housework<br>2 Farming<br>3 Trading/selling<br>4 Hairdressing<br>5 Dressmaking<br>6 Construction work<br>7 Civil/public servant<br>8 Student<br>88 NA<br>other Other
undonework_other	Specify other.<br>*Question relevant when: selected(${undonework}, 'other')*	
worker *(required)*	Did anyone have to do this work in your absence?<br>*Question relevant when: ${cough} =1 and ${symptomsdue} !=4 and ${treatment} =1 and ${undonework} !=8*	1 Yes<br>2 No<br>9 Don't know<br>8 Not applicable
whoworked *(required)*	Who did your work whilst you were attending services at the facility?<br>*MULTIPLE ANSWERS ALLOWED*<br>*Question relevant when: ${cough} =1 and ${symptomsdue} !=4 and ${treatment} =1 and ${undonework} !=8 and ${worker} =1*	1 Daughter<br>2 Sister<br>3 Friend<br>4 Mother<br>5 Husband<br>6 Grandmother<br>7 Working partner<br>8 NA<br>other Other
whoworked_other	Specify other.<br>*Question relevant when: selected(${whoworked}, 'other')*	
avgcomp *(required)*	On average how much do you make in a day for this work you left undone?<br>*Question relevant when: ${cough} =1 and ${symptomsdue} !=4 and ${undonework} !=8*	1 Ghanaian currency<br>2 Nothing/Free<br>3 NHIS beneficiary<br>4 NA<br>5 DK<br>other Other
avgcomp_other	Specify other.<br>*Question relevant when: selected(${avgcomp}, 'other')*	

Field	Question	Answer
Woman of the Household (1) > SECTION 9: CARE OF ILLNESS > Amount<br>*Group relevant when: ${avgcomp} =1*		
compcedi	Cedis	
compGp	Pesawas	
agecheck	Is/Was [name] under three years old?	1 Yes<br>2 No
stooldispose *(required)*	The last time [name] passed stools, what was done to dispose of the stools?<br>*ASK THE FOLLOWING QUESTION ONLY ONCE FOR EACH MOTHER/CARETAKER.*<br>*Question relevant when: ${agecheck} =1*	1 Child used toilet/latrine<br>2 Put/rinsed into toilet or latrine<br>3 Put/rinsed into drain or ditch<br>4 Thrown into garbage (solid waste)<br>5 Buried<br>6 Left in the open<br>9 DK<br>other Other
stooldispose_other	Specify other.<br>*Question relevant when: selected(${stooldispose}, 'other')*	
childsymptoms *(required)*	Sometimes children have severe illnesses and should be taken immediately to a health facility. What types of symptoms would cause you to take your child to a health facility right away?<br>*KEEP ASKING FOR MORE SIGNS OR SYMPTOMS UNTIL THE MOTHER/CARETAKER CANNOT RECALL ANY ADDITIONAL SYMPTOMS . INCLUDE ALL SYMPTOMS MENTIONED, BUT DO NOT PROMPT WITH ANY SUGGESTIONS.*	1 Child not able to drink or breastfeed<br>2 Child becomes sicker<br>3 Child develops a fever<br>4 Child has fast breathing<br>5 Child has difficulty breathing<br>6 Child has blood in stool<br>7 Child is drinking poorly<br>other Other
childsymptoms_other	Specify other.<br>*Question relevant when: selected(${childsymptoms}, 'other')*	
Woman of the Household (1) > SECTION 10: UNDER FIVE MALARIA AND ITN USE<br>*Group relevant when: ${numbirths} >0*		
generated_note_name_1237	Now I would like to ask you questions about any malaria [name] may have had recently	
malaria *(required)*	In the last two weeks, has [name] been ill with a fever (malaria)?	1 Yes<br>2 No<br>9 Don't know<br>8 Not applicable
malariafac *(required)*	Was [name] seen at a health facility during this illness?<br>*Question relevant when: ${malaria} =1*	1 Yes<br>2 No<br>9 Don't know<br>8 Not applicable
malariamed *(required)*	Did [name] take a medicine for fever (malaria) that was provided or prescribed at the health facility?<br>*Question relevant when: ${malaria} =1 and ${malariafac} =1*	1 Yes<br>2 No<br>9 Don't know<br>8 Not applicable
prescribed *(required)*	What medicine did [name] take that was provided or prescribed at the health facility?<br>*Question relevant when: ${malaria} =1 and ${malariafac} =1 and ${malariamed} =1*	1 SP/Fansidar<br>2 Chloroquine<br>3 Amodiaquine/camoquine<br>4 Quinine<br>5 Artemisinin-based combinations<br>6 Paracetamol/Panadol /Acetaminophen<br>7 Aspirin<br>8 Ibuprofen<br>88 NA<br>99 DK<br>other Other
prescribed_other	Specify other.<br>*Question relevant when: selected(${prescribed}, 'other')*	

Field	Question	Answer
givenmed *(required)*	Was [name] given medicine for the fever (malaria) before being taken to the health facility?<br>*Question relevant when: ${malaria} =1*	1 Yes<br>2 No<br>9 Don't know<br>8 Not applicable
givenmed2 *(required)*	Was [name] given medicine for fever (malaria) during this illness?<br>*Question relevant when: ${malaria} =1 and ${givenmed} =8*	1 Yes<br>2 No
prescribed2 *(required)*	What medicine was [name] given?<br>*CIRCLE ALL MEDICINES GIVEN. ASK TO SEE THE MEDICATION IF TYPE IS NOT KNOWN. IF TYPE OF MEDICATION IS STILL NOT DETERMINED, SHOW TYPICAL ANTI-MALARIALS TO RESPONDENT.*<br>*Question relevant when: ${malaria} =1 and ${givenmed2} =1*	1 SP/Fansidar<br>2 Chloroquine<br>3 Amodiaquine/camoquine<br>4 Quinine<br>5 Artemisinin-based combinations<br>6 Paracetamol/Panadol /Acetaminophen<br>7 Aspirin<br>8 Ibuprofen<br>88 NA<br>99 DK<br>other Other
prescribed2_other	Specify other.<br>*Question relevant when: selected(${prescribed2}, 'other')*	
timetomed *(required)*	How long after the fever (malaria) started did [name] first take (NAME OF ANTI-MALARIAL FROM 1104 or 1107)?<br>*Question relevant when: ${malaria} =1 and ${prescribed} <9 or ${prescribed2} <9*	1 Same day<br>2 Next day<br>3 2 days after the fever<br>4 3 days after the fever<br>5 4 or more days after the fever<br>8 NA<br>9 DK
malaria2place *(required)*	Where did you get the (NAME OF ANTIMALARIAL)?<br>*If more than one anti-malarial is mentioned, refer to the first anti-malarial given*<br>*Question relevant when: ${prescribed} <9 or ${prescribed2} <9*	1 Govt. hospital/polyclinic<br>2 Govt. health centre<br>3 CHPS compound<br>4 Village health worker<br>5 Mobile/outreach clinic<br>6 Other public<br>7 Private hospital/clinic<br>8 Private physician<br>9 Private pharmacy<br>10 Mobile clinic<br>11 Other private medical<br>12 Relative or friend<br>13 Shop<br>14 Traditional practitioner<br>88 NA<br>99 DK
malconsultpay *(required)*	Did you have to pay for consultation?<br>*Question relevant when: ${malaria} =1*	1 Yes<br>2 No<br>3 Don't Know
Woman of the Household (1) > SECTION 10: UNDER FIVE MALARIA AND ITN USE > How much?<br>*Group relevant when: ${malaria} =1 and ${malconsultpay} =1*		
malconsultcedi	Cedis	
malconsultGp	Pesawas<br>*Response constrained to: .<100*	
maltranspay	How much did you have to pay for transportation to and from the facility for you and other person with you?<br>*Question relevant when: ${malaria} =1*	1 Ghanaian currency<br>2 Nothing/Free<br>3 NHIS beneficiary<br>4 NA<br>5 DK<br>other Other
maltranspay_other	Specify other.<br>*Question relevant when: selected(${maltranspay}, 'other')*	
Woman of the Household (1) > SECTION 10: UNDER FIVE MALARIA AND ITN USE > How much?<br>*Group relevant when: ${malaria} =1 and ${maltranspay} =1*		

Field	Question	Answer
maltranscedi	Cedis	
maltransGp	Pesawas<br>*Response constrained to: .<100*	
malfoodpay	How did you pay for food for you and other persons with you?<br>*Question relevant when: ${malaria} =1*	1 Ghanaian currency<br>2 Nothing/Free<br>3 NHIS beneficiary<br>4 NA<br>5 DK<br>other Other
malfoodpay_other	Specify other.<br>*Question relevant when: selected(${malfoodpay}, 'other')*	
Woman of the Household (1) > SECTION 10: UNDER FIVE MALARIA AND ITN USE > How much?<br>*Group relevant when: ${malaria} =1 and ${malfoodpay} =1*		
malfoodcedi	Cedis	
malfoodGp	Pesawas<br>*Response constrained to: .<100*	
maltravfac *(required)*	How long did you have to travel to and from the facility?<br>*Question relevant when: ${malaria} =1*	1 Hours<br>2 Minutes<br>8 NA<br>9 DK
maltravhr	Hours<br>*Question relevant when: ${malaria} =1 and ${maltravfac} =1*	
maltravmin	Minutes<br>*Question relevant when: ${malaria} =1 and ${maltravfac} =2*<br>*Response constrained to: .<60*	
malfacwait *(required)*	How long did you have to wait at the facility before you were seen by health personnel?<br>*Question relevant when: ${malaria} =1*	1 Hours<br>2 Minutes<br>8 NA<br>9 DK
malfachr	Hours<br>*Question relevant when: ${malaria} =1 and ${malfacwait} =1*	
malfacmin	Minutes<br>*Question relevant when: ${malaria} =1 and ${malfacwait} =2*<br>*Response constrained to: .<60*	
malwork *(required)*	What work did you have to leave undone to attend services at the facility?<br>*Question relevant when: ${malaria} =1*	1 Housework<br>2 Farming<br>3 Trading/selling<br>4 Hairdressing<br>5 Dressmaking<br>6 Construction work<br>7 Civil/public servant<br>8 Student<br>88 NA<br>other Other
malwork_other	Specify other.<br>*Question relevant when: selected(${malwork}, 'other')*	
worker2 *(required)*	Did anyone have to do this work in your absence?<br>*Question relevant when: ${malaria} =1 and ${malwork} !=8*	1 Yes<br>2 No<br>9 Don't know<br>8 Not applicable
whoworked2 *(required)*	Who did your work whilst you were attending services at the facility?<br>*Question relevant when: ${malaria} =1 and ${malwork} !=8 and ${worker2} !=2*	1 Daughter<br>2 Sister<br>3 Friend<br>4 Mother<br>5 Husband<br>6 Grandmother<br>7 Working partner<br>8 NA<br>other Other
whoworked2_other	Specify other.<br>*Question relevant when: selected(${whoworked2}, 'other')*	

Field	Question	Answer
avgcomp2 *(required)*	On average how much do you make in a day for this work you left undone?<br>*Question relevant when: ${malaria} =1 and ${malwork} !=8*	1 Ghanaian currency<br>2 Nothing/Free<br>3 NHIS beneficiary<br>4 NA<br>5 DK<br>other Other
avgcomp2_other	Specify other.<br>*Question relevant when: selected(${avgcomp2}, 'other')*	
Woman of the Household (1) > SECTION 10: UNDER FIVE MALARIA AND ITN USE > Amount<br>*Group relevant when: ${malaria} =1 and ${malwork} !=8 and ${avgcomp2} =1*		
compcedi2	Cedis	
compGp2	Pesawas<br>*Response constrained to: .<100*	
ITN *(required)*	Does your household have any mosquito net that can be used while sleeping?	1 Yes<br>2 No<br>3 Don't Know
ITNnum *(required)*	How many mosquito nets does your household have?<br>*IF 7 OR MORE NETS , RECORD '7'. 8=NA*<br>*Question relevant when: ${ITN} =1*<br>*Response constrained to: .<9*	
ITNplace *(required)*	Where did you get your last mosquito net from?<br>*Question relevant when: ${ITN} =1*	1 Govt. hospital/polyclinic<br>2 Govt. health centre<br>3 CHPS compound<br>4 Village health worker<br>5 Mobile/outreach clinic<br>6 Other public<br>7 Private hospital/clinic<br>8 Private physician<br>9 Private pharmacy<br>10 Mobile clinic<br>11 Other private medical<br>12 Relative or friend<br>13 Shop<br>14 Traditional practitioner<br>88 NA<br>99 DK<br>other Other
ITNplace_other	Specify other.<br>*Question relevant when: selected(${ITNplace}, 'other')*	
netpay	Did you pay for this mosquito net?<br>*Question relevant when: ${ITN} =1*	1 Yes<br>2 No
bednetpaid *(required)*	How did you pay for this last mosquito net?<br>*Question relevant when: ${ITN} =1 and ${netpay} =1*	1 Ghanaian currency<br>2 Nothing/Free<br>3 NHIS beneficiary<br>4 NA<br>5 DK
Woman of the Household (1) > SECTION 10: UNDER FIVE MALARIA AND ITN USE > How much?<br>*Group relevant when: ${bednetpaid} =1*		
bednetcedi	Cedis<br>*Question relevant when: ${ITN} =1 and ${bednetpaid} =1*	
bednetGp	Pesawas<br>*Response constrained to: .<100*	
childnet *(required)*	Did [name] sleep under a mosquito net last night?<br>*Question relevant when: ${ITN} =1*	1 Yes<br>2 No<br>3 NA
mothernet *(required)*	Did you (mother of youngest child) sleep under a mosquito net last night?<br>*Question relevant when: ${ITN} =1*	1 Yes<br>2 No<br>3 NA
obtainnet *(required)*	How long ago did your household obtain the mosquito net?<br>*Question relevant when: ${ITN} =1*	1 Months<br>8 NA<br>9 DK

Field	Question	Answer
obtainnetmo	Months<br>*IF LESS THAN 1 MONTH, RECORD '0'. IF ANSWER IS "12 MONTHS" OR "1 YEAR", PROBE TO DETERMINE IF NET WAS TREATED EXACTLY 12 MONTHS AGO, EARLIER OR LATER.*<br>*Question relevant when: ${ITN} =1 and ${obtainnet} =1*	
treated *(required)*	When you got that net, was it already treated with an insecticide to kill or repel mosquitoes?<br>*Question relevant when: ${ITN} =1*	1 Yes<br>2 No<br>3 Don't Know
treatedsince *(required)*	Since you got the mosquito net, was it ever soaked or dipped in a liquid to kill/repel mosquitoes or bugs?<br>*Question relevant when: ${ITN} =1*	1 Yes<br>2 No<br>3 Don't Know
dipnet *(required)*	How long ago was the net last soaked or dipped?<br>*Question relevant when: ${ITN} =1 and ${treatedsince} =1*	1 Months<br>8 NA<br>9 DK
dipnetmo	Months<br>*IF LESS THAN 1 MONTH, RECORD '0'. IF ANSWER IS "12 MONTHS" OR "1 YEAR", PROBE TO DETERMINE IF NET WAS TREATED EXACTLY 12 MONTHS AGO, EARLIER OR LATER.*<br>*Question relevant when: ${ITN} =1 and ${dipnet} =1*	
Woman of the Household (1) > GENERAL COMMENTS ABOUT THE INTERVIEW		
reliability	In your opinion, how reliable were the responses?	1 VERY RELIABLE<br>2 RELIABLE<br>3 UNRELIABLE
onrespondent	Comments about respondent:	
onquestions	Comments on specific questions:	
onother	Other comments:	
supcomm	SUPERVISOR'S COMMENTS:	
supname *(required)*	Name of Supervisor	
